# Systems genomics evaluation of the SH-SY5Y neuroblastoma cell line as a model for Parkinson’s disease

**DOI:** 10.1186/1471-2164-15-1154

**Published:** 2014-12-20

**Authors:** Abhimanyu Krishna, Maria Biryukov, Christophe Trefois, Paul MA Antony, Rene Hussong, Jake Lin, Merja Heinäniemi, Gustavo Glusman, Sandra Köglsberger, Olga Boyd, Bart HJ van den Berg, Dennis Linke, David Huang, Kai Wang, Leroy Hood, Andreas Tholey, Reinhard Schneider, David J Galas, Rudi Balling, Patrick May

**Affiliations:** Luxembourg Centre for Systems Biomedicine, University of Luxembourg, Campus Belval, 7, avenue des Hauts-Fourneaux, L-4362 Esch-sur-Alzette, Luxembourg; Institute of Biomedicine, School of Medicine, University of Eastern Finland, Kuopio, Finland; Institute for Systems Biology, Seattle, Washington USA; Systematic Proteomics, Institute for Experimental Medicine, University Kiel, Kiel, Germany; Pacific Northwest Diabetes Research, Seattle, Washington USA

**Keywords:** SH-SY5Y, Cell line, Whole genome sequencing, RNA-seq, Proteomics, Cell line suitability evaluation, Parkinson’s disease

## Abstract

**Background:**

The human neuroblastoma cell line, SH-SY5Y, is a commonly used cell line in studies related to neurotoxicity, oxidative stress, and neurodegenerative diseases. Although this cell line is often used as a cellular model for Parkinson’s disease, the relevance of this cellular model in the context of Parkinson’s disease (PD) and other neurodegenerative diseases has not yet been systematically evaluated.

**Results:**

We have used a systems genomics approach to characterize the SH-SY5Y cell line using whole-genome sequencing to determine the genetic content of the cell line and used transcriptomics and proteomics data to determine molecular correlations. Further, we integrated genomic variants using a network analysis approach to evaluate the suitability of the SH-SY5Y cell line for perturbation experiments in the context of neurodegenerative diseases, including PD.

**Conclusions:**

The systems genomics approach showed consistency across different biological levels (DNA, RNA and protein concentrations). Most of the genes belonging to the major Parkinson’s disease pathways and modules were intact in the SH-SY5Y genome. Specifically, each analysed gene related to PD has at least one intact copy in SH-SY5Y. The disease-specific network analysis approach ranked the genetic integrity of SH-SY5Y as higher for PD than for Alzheimer’s disease but lower than for Huntington’s disease and Amyotrophic Lateral Sclerosis for loss of function perturbation experiments.

**Electronic supplementary material:**

The online version of this article (doi:10.1186/1471-2164-15-1154) contains supplementary material, which is available to authorized users.

## Background

Cell lines are widely used for perturbation experiments that aim to understand disease mechanisms at a cellular level. Cells used in such experiments are only rarely an inherent biological model for the disease of interest. Most commonly, genetic or environmental perturbations are required to create cellular responses, which can then serve as an experimental disease model. It is known that many cell lines carry major genetic variations, which would be lethal for humans at the stage of prenatal development. The main advantage of highly proliferative cell lines relative to primary cells and induced pluripotent stem cells is a significantly greater capacity for experiments that require large amounts of clonal cells with identical genetic background, such as needed in state-of-the-art high-throughput screening, especially in proteomics or metabolomics. The availability of clonal cells allows the possibility for comparative perturbation experiments aiming to compare phenotypic outputs derived from a set of single node perturbations [1]. In addition, using cell lines avoids the ethical concerns arising out of human primary neuronal cell culture. The human neuroblastoma cell line SH-SY5Y first described in [2], is a commonly used cell line in studies related to neuroblastoma and neurodegenerative disease. The cell line is a sub-clone of the parent cell line SK-N-SH which was originally established from a bone marrow biopsy of a neuroblastoma patient [3]. The three human diseases most frequently mentioned in literature for SH-SY5Y were neuroblastoma, Alzheimer’s disease (AD), and Parkinson’s disease (PD) (Additional file 1). A complete genomic characterization of SH-SY5Y would thus elucidate the applicability and possible limits for modelling these disease-specific processes in the context of this cell line.

Undifferentiated SH-SY5Y cells have been extensively used as an *in vitro* model for research in neuroscience [4]. The cell line shows biochemical properties of immature catecholaminergic neurons [2]. Studies have found that undifferentiated SH-SY5Y express only immature neuronal markers and lack mature neuronal markers [2, 5]. Thus, undifferentiated SH-SY5Y cell line might not represent an appropriate model for diseases such as PD, which primarily affect differentiated dopaminergic neurons [6]. Further, Xie et al. [4] reviewed 60 articles on SH-SY5Y cell as *in vitro* model for PD research and found that differentiation of SH-SY5Y under a certain treatment results in a more dopaminergic neuronal phenotype, which could be extremely useful for modelling selective dopaminergic cell death in PD. However, they point out that an optimally differentiated SH-SY5Y dopaminergic cell model requires further research. A systems genomics analysis could thoroughly assess the genetic mutations in all neuronal markers and therefore place limits on how closely differentiation can model a dopaminergic cell.

Furthermore, treatment with differentiation-inducing agents enable SH-SY5Y cells to become morphologically similar to mature primary neurons [7], and the different treatment agents (e.g. retinoic acid, phorbol esters, dibutryl cyclic adenosine monophosphate) result in a variety of neuronal phenotypes (e.g. cholinergic, dopaminergic, noradrenergic) [8]. Genomic mutations may, however, impose limitations on the possible phenotypes to which SH-SY5Y can differentiate. Therefore, a complete genomic characterisation of the SH-SY5Y cell line can inform the limitations on the possible phenotypes imposed by the genome.

**Figure 1:**
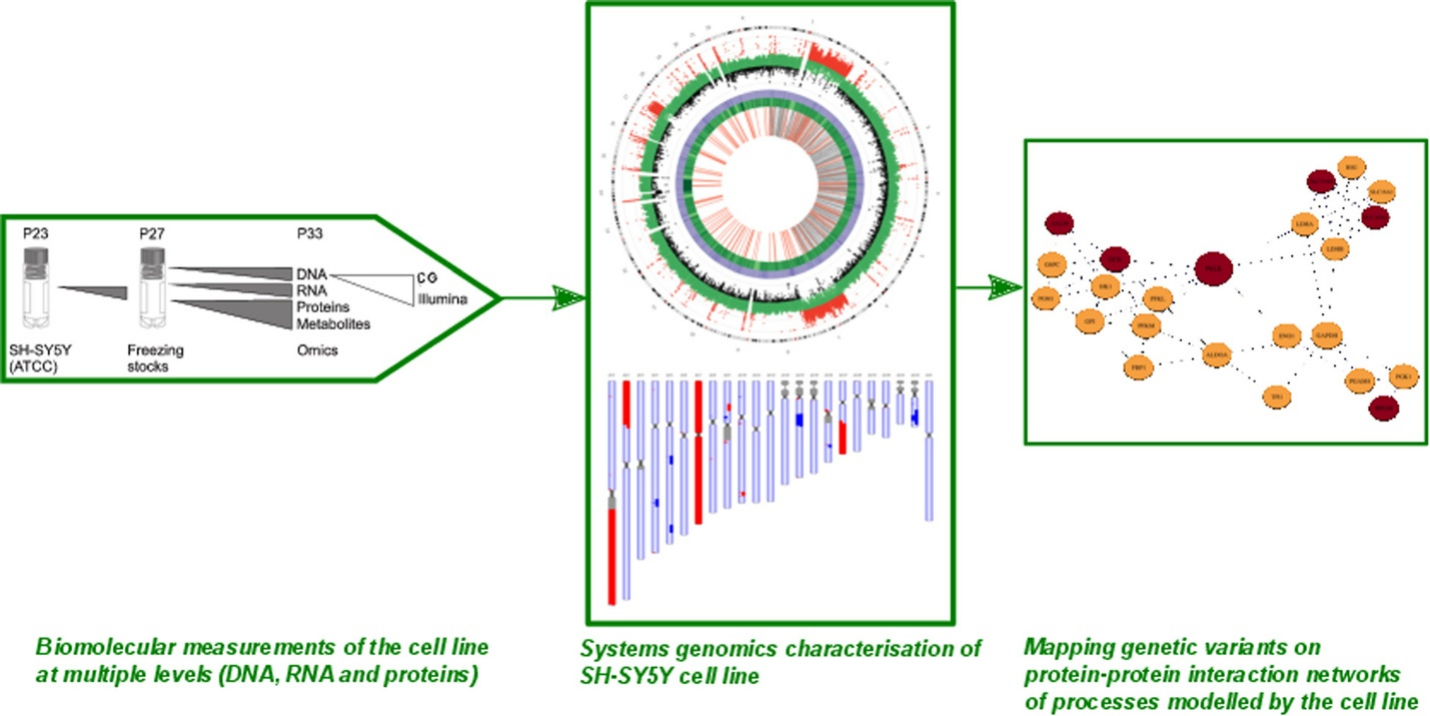
**Systems genomics approach to assess SH-SY5Y as a disease model.**

Systems genomics aims to integrate genomic variation, copy number and structural variation with high-throughput gene expression, metabolomics and proteomic data to explore the genetic architecture of complex traits and multi-factorial diseases. For characterizing the SH-SY5Y cell line we integrated information from whole genome sequencing, transcriptomics, and proteomics experiments (Figure 1). Firstly, whole-genome sequencing is used to determine the genetic background of the cell line. Secondly, transcriptomics and proteomics data is used to study correlation across biological levels. Finally, we integrate genomic variants using a network analysis approach to evaluate the suitability of the SH-SY5Y cell line as an *in vitro* model to study various neurodegenerative diseases, including PD.

## Results

### Whole genome sequencing

The whole genome of SH-SY5Y cell line was re-sequenced using two different sequencing platforms, those from Complete Genomics (CG) and Illumina (IL) HiSeq2000, to generate a high-quality list of variants. An earlier study has shown the utility of combining information from both platforms and although they show high concordance, each platform alone failed to detect a significant number of exonic variants spread over 1,676 genes [9].

For the SH-SY5Y cell line, DNA sequencing by CG [10] and IL [11] produced a genome-wide coverage of 57× and 49× respectively. (Additional file 2: Table S2) More than 90% of the genome was covered by a minimum read depth of 20 (Additional file 2: Figure S1 and S2). Further, more than 95% of the exome was called with minimum required confidence set by each of the proprietary genotype-calling procedures of the two respective sequencing platforms. The two genome-sequencing platforms – CG and IL – produced a total union of 3,896,055 single nucleotide variations (SNVs) with 84% concordance between both platforms. Filtering them based on criteria defined in Reumers et al. [12] resulted in 2,314,627 SNVs with 99% concordance between both platforms. Out of these high quality variants 96% were previously reported by dbSNP build 137 [13] and 98.4% by 1000 Genomes Project [14, 15]. 4,336 SNVs and small indels were earlier reported by the Catalogue of Somatic Mutations in Cancer (COSMIC) [16] version 64. The total number of SNVs and the percentage of SNVs found in 1000 Genomes Project are similar to controls (mean number of SNVs = 4,178,701 and 87.7% of them were found in 1000 Genomes Project, see Additional file 2: Table S7) in another study [17] taken from the Human Genome Diversity Project [18]. Among the high confidence set of mutations in SH-SY5Y, 23 were confirmed as somatic in the COSMIC database and were also rare in the general population (see Table 1).

**Table 1:** **High-confidence mutations in SH-SY5Y that were rare in the population and also confirmed as somatic in the COSMIC database**

Chromosome:begin-end	COSMIC ID (tissue type)	Genes (or adjacent genes)
2:174118525-174118526	140009 (skin)	MLK7-AS1
3:3965330-3965331	146267 (haematopoietic_and_lymphoid_tissue)	LRRN1(dist = 75944), SETMAR(dist = 379657)
3:97631173-97631174	166940 (large_intestine)	ARL6(dist = 113801), MINA(dist = 29487)
3:97680355-97680356	166941 (large_intestine)	MINA
3:195017896-195017897	212624 (breast)	ACAP2
4:62000660-62000661	200267 (large_intestine)	LOC255130 (dist = 3929195), LPHN3(dist = 362178)
5:40086690-40086691	145684 (haematopoietic_and_lymphoid_tissue)	DAB2(dist = 661356), PTGER4(dist = 593341)
6:35837057-35837058	167752 (large_intestine)	SRPK1
6:152632032-152632033	167911 (large_intestine)	SYNE1
6:168431497-168431498	85018 (pancreas)	KIF25
7:127075991-127075992	200565 (large_intestine)	ZNF800(dist = 43225), GCC1(dist = 144690)
8:27913552-27913553	1098826, 1098827 (endometrium)	C8orf80
8:38006195-38006196	187133 (large_intestine)	STAR
9:6254465-6254466	1109518 (endometrium)	IL33
11:57734912-57734913	146001 (haematopoietic_and_lymphoid_tissue, large_intestine)	TMX2-CTNND1(dist = 148261), OR9Q1(dist = 56440)
12:7585976-7585977	179792 (large_intestine)	CD163L1
12:11905442-11905443	180918 (large_intestine)	ETV6
12:88344608-88344609	433706 (breast)	MKRN9P(dist = 166121), C12orf50(dist = 29207)
14:72128130-72128131	195414 (large_intestine)	SIPA1L1
16:12798881-12798882	1202185 (large_intestine)	CPPED1
19:11134250-11134251	1161250, 1161251 (haematopoietic_and_lymphoid_tissue)	SMARCA4
X:47039372-47039373	1121715 (endometrium)	RBM10
X:104440586-104440587	487453 (kidney)	IL1RAPL2

Rare mutations are SNVs and small indels that were found in less than 5% of the samples in 1000 Genomes Project, Exome Sequencing Project and Complete Genomics baseline genomes. In the genes column, when distances are given, the mutations are found in intergenic regions and the first gene precedes the mutation whereas the second gene succeeds the mutation.

The number of private protein-altering (PPA) SNVs and indels, i.e. protein-altering variants not found in 1000 Genome Project or the Exome Sequencing Project [19] were 75 and 34 respectively (1,598 SNVs and 305 indels before filtering), whereas the corresponding numbers for controls were 390.9 and 17.5. The set of control genomes refers to 11 genomes used in another study [17] taken from the Human Genome Diversity Project [18]. The reason for the discrepancy between the number of PPA SNVs between SH-SY5Y and the control genomes is mainly due to our strict filtering strategy as well as the different sequencing platforms that were used (See Additional file 2 – section S6 for further details). The overlap between PPA variants and the variants in the Catalogue of Somatic Mutations in Cancer (COSMIC) was 36 and 8.7 for SH-SY5Y and control genomes respectively. The overlap between genes containing PPA SNVs and indels and the Sanger Cancer Gene Census (SCGC) [16] was 7, similar to controls (mean *n* = 5).

We also performed a Gene Ontology (GO) enrichment analysis on the 121 genes containing filtered PPA SNVs and indels and also on the 1,365 genes using unfiltered PPA SNVs and indels. However, no GO term was significantly enriched (see Additional file 3).

We judged the overall quality of the variant calls and also compared them between the two genome sequencing platforms - CG and IL. Then, we integrated variant calls from both these platforms by using the filtering criteria provided by an earlier study [12] that compared these two platforms (See section S2 for an overview of the comparison between CG and IL sequencing platforms).

### Validation of SNVs and small indels

Validation was performed using the Illumina Omni-1 Quad microarray, which assays loci from HapMap Phase 1–3 [20] and 1000 Genomes Project. Out of 248,538 heterogeneous SNVs that were queried by genotyping, 99.0% were concordant, which increased to 99.5% for the filtered variants (Additional file 2: Table S11).

### Functional prediction of SNVs and small indels

The functional effect of SNVs and small indels were predicted using ANNOVAR [21] by annotation with labels for genomic regions (intergenic, exonic, intronic, un-translated regions, upstream and downstream close to a gene) and coding effects (SNVs - synonymous, missense, stop-gain, stop-loss - and indels - frameshift and non-frameshift). The annotation was performed by taking a consensus across four databases (RefSeq refgene release 55 [22], UCSC knowngene [23], Ensembl ensgene v65 [24] and GENCODE V4 [25]) based on choosing the most damaging effect predicted in order to aim for sensitivity (Additional file 2: Figures S4–S9). In general, the association of SNVs to genomic regions for platform-specific and concordant SNVs did not show any significant differences. The association of SNVs with exonic, intronic and intergenic regions was 1%, 26–36% and 46–50% respectively, similar to the results in Lam et al. [9] (Additional file 2: Figure S4 and S5). The functional prediction found 366 genes with rare non-synonymous SNVs, indels or substitutions (<5% frequency in 1000 Genome Project, 6500 Exome Sequencing Project and CG Baseline Genome Dataset).

### Neuroblastoma-relevant genes

The genome sequencing of SH-SY5Y found 27 genes with rare non-synonymous SNVs, indels or substitutions that overlapped with the list of 586 genes containing somatic mutations in the complete genome sequence of 87 untreated primary neuroblastoma tumours [26] (See Additional file 2: Table S12). Using the hyper-geometric test, genes with rare non-synonymous SNVs and indels were found to be significantly enriched among the genes with somatic mutations in primary neuroblastoma tumours (p-value = 6.80 × 10^-13^). Out of these 27 genes, only mutations in 15 genes were predicted as damaging by Sorting Intolerant From Tolerant (SIFT) [27].

Furthermore, only 3 genes with rare non-synonymous SNVs, indels and substitutions overlapped with the 118 genes associated with neuroblastoma in the literature (see Methods section) and therefore were not significantly enriched (p-value = 0.072). However, genes with non-synonymous SNVs, indels and substitutions, copy number variations, and structural variations (*n* = 3611) were significantly enriched among the 118 genes extracted for neuroblastoma from literature with an overlap of 28 genes (p-value = 1.59 × 10^-7^).

We also compared genomic mutations in SH-SY5Y to other genomic sequencing studies of primary neuroblastoma from patients. Among the seven genes mutated at a significant frequency in a study of 240 matched tumour -normal samples [28], none of the genes carried a rare amino-acid changing mutation. Among the rare germ-line variants predisposing to neuroblastoma identified by the TARGET study, *PALB2* contained two rare non-synonymous mutations in SH-SY5Y. Among the 5,291 coding somatic mutations found in an aggregate of 240 matched tumour/normal samples, we found 26 overlapping variants (Additional file 3) in SH-SY5Y inside 24 genes among which 9 were rare amino-acid changing mutations inside 7 genes (*ALK*, *FOXD4L1*, *HLA-DRB1*, *NBPF10*, *NBPF14*, *PABPC3*, *TEKT4*).

The Pediatric Cancer Genome Project (PCGP) conducted a study of 40 patients with metastatic neuroblastoma and found mutations in *ATRX* and *ALK* in 22% and 14% of the patients correspondingly [29] *.* However, SH-SY5Y contained no rare amino-acid-changing mutations in these genes.

### Structural Variations (SV) and Mobile Element Insertions (MEI)

Structural variation events consist of large deletions (>200 base-pairs), duplications (distal or tandem), inversions, translocations and complex variations - other combinations of such chromosomal rearrangements. The majority of SV events in the SH-SY5Y cell line found by CG were deletions (Additional file 2: Table S12 and S13).

Around 2–3% of cancers show chromothripsis [30], where tens to hundreds of genomic rearrangements occur in a cellular crisis event. In a certain region, a high density of genomic rearrangements (or breakpoints) combined with frequent oscillations between two copy number states [30] and frequent occurrences of runs of homozygosity constitute the hallmark of chromothripsis. Cytogenetic methods confirm a high density of genomic breakpoints in single cells and indicate that these breakpoints are not a result of parallel rearrangements in different sub-clones [30].

However, for SH-SY5Y, DNA sequencing found little to no evidence of chromothripsis. Most notably, no oscillations between copy number states of 100-kb binned read depth were observed except for some on chromosomes 9, 10, 16 and X (Additional file 4). In all of these four chromosomes, the regions with oscillating copy number were near the centromeres (9, 10, 16) or the telomere (16, X). Even though all of these regions have at least six junctions, these regions consist of highly repetitive DNA [31] and are therefore prone to mapping errors.

We filtered the SVs based on their frequency in the CG baseline genome set [32] and extracted high-confidence calls with a frequency of less than 10% in the baseline. The CG baseline set comprises of 52 genomes of healthy, disease-free individuals, which could be used to filter technical artifacts and variations common in the population. After filtering, the high-confidence deletions overlapped with 26 genes including the following cancer-related genes - *PTEN* (phosphatase and tensin homolog), which is a key tumour suppressor gene [33], *CTNNA3* (catenin alpha 3), which is a cell contact inhibition gene whose mutation can promote cancer development and formation, *MCC* (mutated in colorectal cancers) and *MTUS1* (microtubule associated tumour suppressor 1).

Mobile element insertions (MEI) refer to insertion of sequences that can change their position within the genome. 3,271 MEI events were detected in the CG data (Additional file 2: Table S14) and the majority of those were Alu events (*n* = 2,057) and L1 retro-transposons (*n* = 2,057).

### Copy number variation (CNV)

CG identifies discrete coverage levels corresponding to ploidy levels using the distribution of observed normalized coverage values and then uses a hidden Markov model to assign copy number levels (Complete Genomics Data File Formats Standard Pipeline 2.4 [34]). Since CG essentially uses the relative coverage as a proxy for copy number, it cannot directly infer the absolute copy number levels. However, M-FISH (multiplexed fluorescent in-situ hybridization) experiments have demonstrated that diploidy dominates the copy number level in the SH-SY5Y genome [35]. Therefore, we calculated the absolute copy number levels assuming that the most commonly found copy number level is 2.

**Figure 2:**
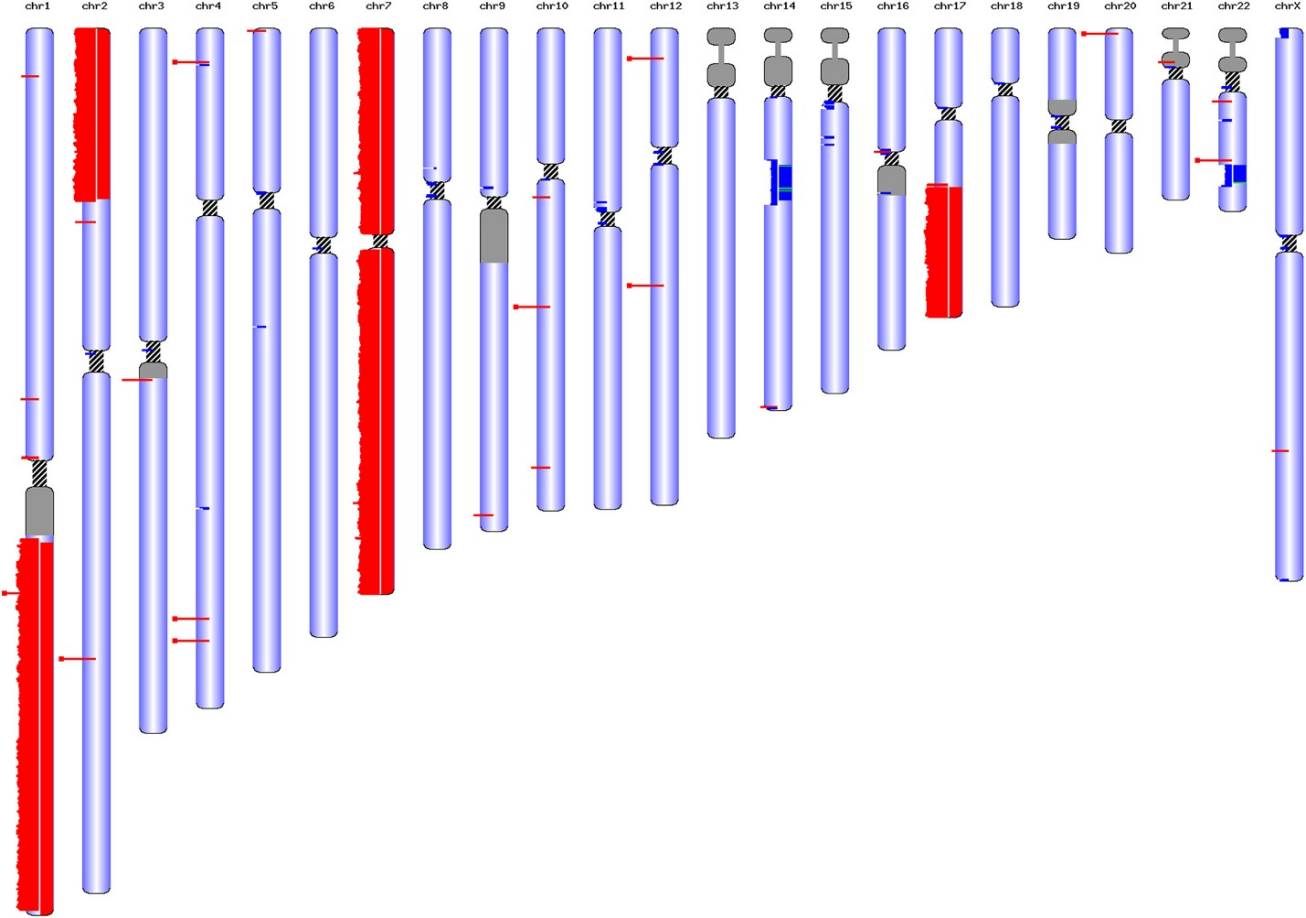
**CNVs detected by whole genome sequencing and array-based CGH by Do et al.** [36]**.** The results from whole genome sequencing were from Complete Genomics and are given in the left half of the chromosomes. The results from the array-based CGH are on the right half of the chromosomes. Regions are highlighted for copy number gain (red) and loss (blue). The major events partial trisomy of chromosome 1 and 2, complete trisomy of chromosome 7, gain in 17q and loss in 22q were confirmed. (Generated using http://db.systemsbiology.net/gestalt/cgi-pub/genomeMapBlocks.pl).

We compared the copy number levels found by CG with an earlier measurement using comparative genomic hybridization (CGH) arrays [36]. The CGH arrays used had a lower limit one million base pairs to their resolution, a drawback not found in second-generation sequencing technologies such as CG and IL. To achieve even higher resolutions and reduce false positive and false negative copy number determinations, we normalized the coverage levels to those observed in a reference set of 590 in-house genomes also sequenced by CG. A visual illustration of the copy number levels (Figure 2, Additional file 2: Figure S21) confirms key features of the cell line described previously such as partial trisomy of chromosome 1, gain of chromosome 7, 2p, 17q, and loss of 1p, 14q and 22q. Among the large-scale copy number features identified in the PCGP project, the gain of 17q (90%) was common with SH-SY5Y. The remaining CNVs that were reported with a lower frequency in PCGP - loss of 1p (43%) or loss of 11q (43%) were not found in SH-SY5Y.

Interestingly, the strongest clinical marker of neuroblastoma that also determines the aggressiveness of neuroblastoma [36] - gain of the gene *MYCN* (myelocytomatosis viral-related) – actually had a relative coverage of only 3. However, more recently, [35] confirmed the finding that the *MYCN* gene is not strongly amplified in SH-SY5Y. Also, among the 104 patients sequenced in the PCGP project, only 23% carried an amplification of *MYCN*, which was defined as >10 copies detect by quantitative-PCR.

### Transcriptome analysis

To characterize the SH-SY5Y cell line gene expression, we sequenced poly-adenylated RNA from undifferentiated SH-SY5Y cells using a stranded RNA-seq protocol. We used the GRCh37 human genome version together with the Ensembl release 66 [24] gene annotation. We generated about 100 million paired-end reads of lengths 50 nt. The raw reads are available in the European Nucleotide Archive (ENA) database [37] under the study accession PRJEB7313. In total, 94 million paired-end reads and 4.6 million singleton reads could be mapped after quality control (QC) filtering and trimming to either the genome or the transcriptome annotation. This yields in 95% mappable reads to either genome or cDNA sequences.

For RNA-seq, we called SNVs and small indels using both SAMTools [38] and the Genome Analysis Toolkit (GATK) [39]. We retained the intersection of the two outputs and those caller-specific variants supported with a read depth greater than or equal to 10. As a recent study has shown low concordance between multiple variant-calling pipelines [40], we decided to increase the level of confidence by using two variant-calling pipelines. Consequently for RNA-seq, out of 95,173 SNVs and small indels detected, 70.2% were concordant with unfiltered variants from at least one DNA sequencing platform, either CG or IL (Additional file 2: Table S17). The RNA-seq also showed high sensitivity as 95.1% of the 3,500 filtered exonic variants found through DNA sequencing in genes with FPKM > 5 were also detected by RNA-seq. FPKM refers to Fragments Per Kilobase of exon per Million fragments mapped, which is a measure of gene expression. This sensitivity of RNA-seq for variant detection was higher than that achieved by a recent approach for SNV calling in RNA-seq data [41] but the absolute number of detected exonic variants (*n* = 3,300) was slightly lower than theirs (*n* = 4,000), which might be due to our strict quality filtering of the DNA sequencing data. As for neuroblastoma-relevant genes from the 87 neuroblastoma primary tumours, out of the 30 rare, amino-acid changing mutations (in 27 genes), 10 of them were detected by RNA-seq with the same zygosity. The remaining 20 were likely not detected because of low gene expression as all mutations detected by RNA-seq were in genes with FPKM > 3.1 and all mutations not found by RNA-seq were in genes with FPKM < 0.57.

### Genetic copy number vs. gene expression

We compared genes that were always expressed in SH-SY5Y across 247 different conditions (Additional file 5) from the GEO (Gene Expression Omnibus) database [42] to those that were never expressed, whenever the corresponding probe was present. Here, the threshold used to determine whether genes were always expressed in the GEO dataset was that the expression values of the corresponding transcripts were always greater than the median for all the transcripts in each microarray experiment. Similarly, the threshold for genes never expressed in the GEO dataset was that the expression values were always less than the median for all the transcripts in each microarray experiment. The frequency of mutations per 100 kilo-base-pair region in genes that were never expressed was 165.36, which decreased to 131.31 for genes, which were always expressed. Similarly, the average copy number for genes always expressed was 2.18, which was lowered to 2.09 for genes never expressed (Additional file 2: Table S22). The distribution of expression of genes with copy number greater than 2 was significantly greater (Welch’s t-test p-value = 2.2 × 10^-16^) than expression of genes with copy number lower than or equal to 2 (Additional file 2 – section S27).

Further, we compared the genetic copy number and the RNA-Seq data of the same SH-SY5Y sample. The copy number and the logarithm of Fragments per kilo-base of transcripts per million mapped reads (FPKM) showed a positive correlation (Additional file 2: Figure S24).

### Proteome analysis

In order to confirm and correlate the biological expression of the identified genes, the analysis of genomic data was integrated with the analysis of proteomics data analysis of SH-SY5Y cell lysates (see Methods section for complete details). Proteins isolated from whole SH-SY5Y cell lysates were fractionated by SDS-PAGE, in-gel digested using trypsin, and the recovered peptides analysed by LC-ESI MS/MS. The spectra were analysed by MaxQuant against a combinatorial human protein database. This database contained the human genome reference hg19 protein entries combined with the sequence variants found from the DNA sequencing done here. Specifically, the coding sequences of Ensembl genes were modified using all the homozygous exonic variants (unfiltered).

In total, out of 334,065 acquired MS/MS spectra, 165,494 were matched to a peptide that could be mapped to a total of 1,410 protein identifiers (Additional file 2: Table S21). From these, 1,944 protein groups corresponding to 1,355 proteins were identified in the human genome reference hg19 protein annotation. Further, 45 proteins were identified using sequence variants found from the DNA sequencing done here. See Additional file 6 for the abundances of proteins detected.

To increase peptide spectrum matches, we extended the human genome reference hg19 protein database by inserting reference sequences containing the homozygous SNVs and indels in the exonic regions identified in this study. The peptides that were additionally mapped to the extended protein database were then used for validation of genetic variants (Additional file 2 – section S24). This approach validated 104 SNVs, 4 indels and 1 block substitution.

### Gene expression vs. protein expression

Gene expression levels from RNA-seq and the corresponding protein abundance were compared to detect correlation. We plotted the logarithm of FPKM against the logarithm of intensity–based absolute quantification (iBAQ) [43] score of 1,307 genes (Additional file 2: Figure S25 and Table S25), which were detected in both RNA-seq and proteomics, which equals 93% of 1,410 proteins detected by proteomics. As expected, the correlation was weakly positive (correlation coefficient = 0.2784, p = 10^-27^, see Additional file 2: Figure S26).

### Network-based analysis of suitability of SH-SY5Y cell line as *in vitro*model

**Figure 3:**
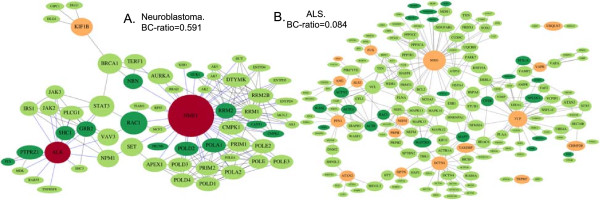
**Protein-protein interaction network for two diseases – neuroblastoma (A) and for ALS (B).** Red nodes refer to OMIM-derived genes mutated in the cell line. Orange nodes refer to the OMIM-derived genes which are intact in the cell line. Dark green nodes represent genes coming from the network expansion, which are mutated in the cell line. Light green nodes represent genes coming from the network expansion, which are intact in the cell line. Nodes are scaled to the magnitude of their betweenness centralities. Blue edges show connections between pairs of nodes in which at least one was damaged in the cell line. For neuroblastoma, mutations in the central genes – NME1 and ALK contribute to the high BC-ratio. For the ALS network, mutations occur in genes with lower betweenness centrality which results in the lower BC-ratio.

**Figure 4:**
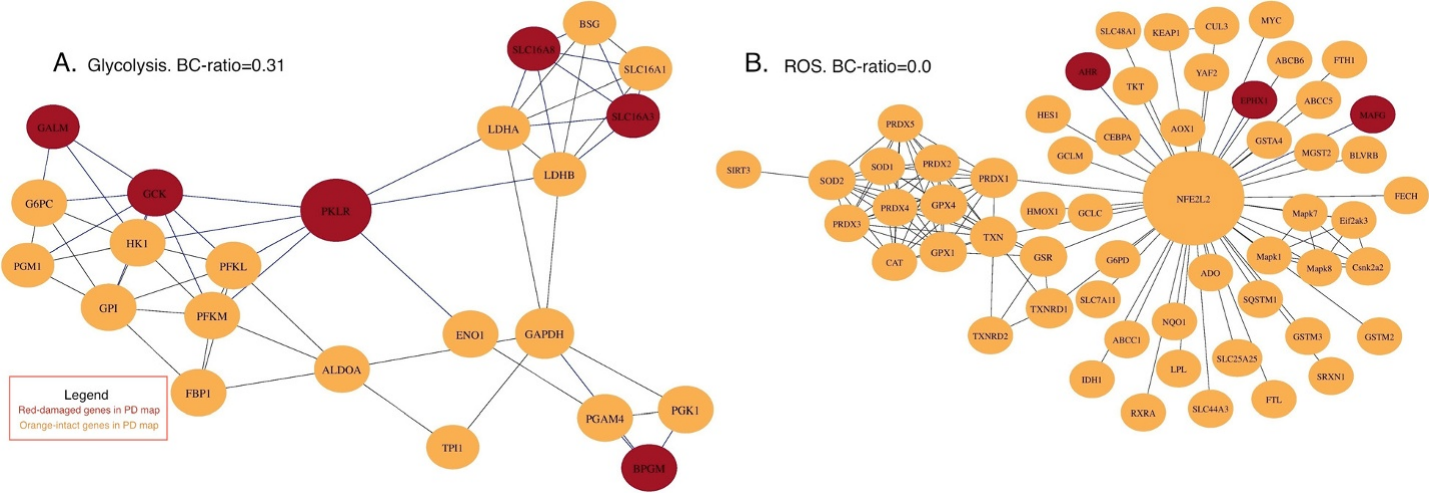
**Protein-protein interaction network for two PD map modules - glycolysis (A) and ROS metabolism (B).** Red nodes refer to genes mutated in the cell line. Orange nodes refer to genes which are intact in the cell line. Nodes are scaled to the magnitude of their betweenness centralities. Blue edges show connections between pairs of nodes in which at least one was damaged in the cell line. For glycolysis, mutations in the central genes lead to the high BC-ratio. Mutated genes lie on the periphery of the ROS metabolism network resulting in a low BC-ratio.

In order to evaluate the suitability of SH-SY5Y as *in vitro* model we adopted a framework described in [44] that integrated genetic sequence information, and topology analysis of either disease or process-specific networks. We applied betweenness-centrality ratio (BC-ratio), a metric that allowed us to quantitatively assess the impact of genes mutated in the cell line on the disease or process network. BC-ratio is normalized to lie between 0.0 and 1.0. It indicates how much the information flow in the network might be altered by changes in the cell line: the higher BC-ratio, the greater the impact of the mutations, the lower the genetic integrity (or suitability) of the cell line in the context of a specific network. (See Methods section for a detailed discussion of the approach). The BC-ratio metric applied to four neurodegenerative diseases (Table 2) ranked Alzheimer’s (AD) as highest BC-ratio = 0.246, p_BC_-value = 0.04), followed by Parkinson’s disease (BC-ratio = 0.186, p_BC_-value 0.168), Huntington disease (BC-ratio = 0.146, p_BC_ value = 0.472), and Amyothrophic Lateral Sclerosis (BC-ratio = 0.084, p_BC_-value = 0.922 ). To investigate the appropriateness of the cell line for perturbation experiments in the context of PD, we also scored the different processes corresponding to the hallmarks of PD (Table 3). See Figure 3 for a protein-protein interaction network resulting in the cell line suitability scores for neuroblastoma and ALS and Figure 4 for the network for glycolysis and reactive oxygen species (ROS) metabolism in PD. Additional file 7 contains network visualizations for each disease and each module in PD. The role of the visualization is to give an idea of how damaged genes are distributed in the network, to which genes they are connected and how they could alter processes of interest.

**Table 2:** **Network statistics and cell line scoring of the neurodegenrative diseases and neuroblastoma**

Disease name	Nodes			Edges	Network centralization (Betweenness)	Cell line suitability based on centrality metrics				p _BC_-value
	Total	Damaged	Intact			Degree	Closeness	BC-ratio	Flow BC	
NB	63	15	48	192	.650	.362	.248	.591	.430	.010
AD	248	40	208	929	.405	.208	.161	.246	.215*	.040
PD	358	55	33	1862	.246	.156	.153	.186	.176*	.168
HD	63	10	53	153	.624	.183	.163	.146	.162	.472
ALS	178	22	156	371	.399	.116	.171	.084	.126*	.922

Flow BC stands for flow betweenness centrality. The P_BC_-value refers to the network randomization test described in the Methods section. Flow Betwenness is defined for connected networks. If the entire network is not connected, Flow betweenness is computed for connected components. In the table the flow betweenness centrality of the largest connected component is given and marked by *. NB – Neuroblastoma; AD – Alzheimer’s disease; PD – Parkinson’s disease; HD – Huntington’s disease; ALS – Amyotrophic Lateral Sclerosis.

**Table 3:** **Network statistics and cell line scoring of the Parkinson’s disease modules**

Module name	Nodes			Edges	Network centra-lization (Bet-weeness)	Cell line suitability based on centrality metrics				p _BC_-value
	Total	Damaged	Intact			Degree	Closeness	BC-ratio	Flow BC	
Glycolysis	23	6	17	59	.321	.262	.256	.310	.253	.330
Mitochondria	259	32	227	4752	.127	.135	.137	.155	.128*	.297
Calcium signalling	125	17	108	561	.160	.163	.132	.149	.095*	.296
Apoptosis	122	15	107	250	.334	.080	.117	.146	.098*	.279
Dopamine	54	8	46	175	.030	.174	.179	.115	.224*	.448
Ubiquitin protease system	55	7	48	809	.070	.150	.153	.044	.133*	.340
ROS metabolism	58	3	55	121	.922	.012	.051	.000	.000	.375

Flow BC stands for flow betweenness centrality**.**The P_BC_-value refers to the network randomization test described in the Methods section. Flow Betwenness is defined for connected networks. If the entire network is not connected, Flow betweenness is computed for connected components. In the table the flow betweenness centrality value of the largest connected component is given and marked by *.

## Discussion

The complete system-wide omics analysis of the undifferentiated SH-SY5Y cell line provided many opportunities for studying concordance across biological levels (DNA, RNA, protein). Firstly, protein-altering variants, copy number variations and structural variations show consistency with the expression level of genes in the RNA-seq data. For instance, variant calling from RNA-seq data showed 95.1% sensitivity for the 3,500 filtered variants detected from DNA-seq in genes with an FPKM > 5. Secondly, sequencing both the DNA and the RNA showed a high degree of concordant SNVs and indels (95.1% for genes with FPKM > 5 were detected by RNA-seq) and served as further validation of DNA sequencing results and our filtering procedure for genomic variants. Finally, the knowledge of genomic variants enhanced protein identification in proteomics experiments where it increased the number of proteins detected from 1,365 to 1,410.

A comparison of the two whole genome sequencing platforms – Complete Genomics (CG) and Illumina (IL) – confirmed a significant number of variants that were discordant, either due to regions, which were not called by one of the platforms or due to platform-specific errors. Considering that 1% (n = 4,321) of these platform-specific variants residing in 2,348 genes were associated with exonic regions, using both platforms would increase the detection of potentially functionally important SNVs. Also only 75–80% of platform-specific variants were found in the known list of variants (in dbSNP, 1000 Genomes Project or 6500 Exome Sequencing Project), indicating that a significant number of mutations are somatic mutations or sequencing errors that need further investigation. Further, the larger platform-specific biases for small indels and structural variations also strengthened the argument for using both platforms for greater sequencing accuracy.

### Neuroblastoma-relevant genetic variations

Without the genome sequence of the healthy cells from the patient, the difficulty of identifying somatic mutations in SH-SY5Y precludes any direct comparison to the somatic mutations in primary neuroblastoma tumours. Thus, we compared rare mutations in SH-SY5Y with the somatic mutations found in 87 primary neuroblastoma tumours. The union of genes from 87 different patient samples resulted in a list of 586 genes, which we considered as relevant to neuroblastoma. The result that only 15 genes with rare amino-acid changing mutations predicted as damaging in SH-SY5Y overlapped with the list of 586 genes with somatic amino-acid changing mutations in primary neuroblastoma was expected as there was considerable heterogeneity among the different tumour samples themselves. Specifically, the sequencing of 87 neuroblastoma primary tumours found few recurrent genes with amino-acid changing mutations - only 24 genes contained amino-acid changing mutations in more than 1 tumour sample and only three genes in more than two tumour samples. In this context, an overlap of 15 genes with rare amino-acid changing mutations suggests that SH-SY5Y includes elements of the genetic architecture of a variety of neuroblastoma tumours. Therefore, it might offer opportunities to investigate traits arising from amino-acid changing mutations found in different kinds of neuroblastoma tumours.

As for structural variations, recurrently occurring structural alterations in the neuroblastoma primary tumours in *PTPRD*, *ODZ3* and *CSMD1* were not found among high-confidence rare structural alterations in SH-SY5Y. Finally, chromothripsis, which was earlier identified in 18% of primary neuroblastoma, was not detected in SH-SY5Y. Therefore, SH-SY5Y would not serve as an appropriate model for neuroblastoma tumours suffering from chromothripsis.

### Suitability of SH-SY5Y as an *in vitro*model for neurodegenerative diseases

The BC-ratio relies on the network betweennness centrality to quantify genetic changes in the cell line. It is important to consider that the BC-ratio does not aim to interpret the functional effects of these mutations. Indeed, mutations could cause a loss, gain, change or no change in the function. However, such interpretations require assumptions. In the case of neuroblastoma, we know that the SH-SY5Y cell line was derived from a neuroblastoma patient. Hence, it can be assumed that many genetic mutations, which were observed in the neuroblastoma network (BC-ratio = 0.591, p_BC_-value 0.01), are indeed typical neuroblastoma mutations. Specifically, copy number variations in the central neuroblastoma genes *NME1* and *ALK* (Figure 3A), both of which are found to be mutated in neuroblastoma as well support this reasoning. Briefly, it can be assumed that SH-SY5Y cells are a good model for neuroblastoma cells and a high BC-ratio (Table 2) was expected.

However, this case where a neuroblastoma cell line is expected to model neuroblastoma is a special case. This interpretation is similar to the approach of Domcke et al. [45] of choosing the ‘most suitable models’ for ovarian tumour among ovarian cancer cell lines based on genomic and mRNA expression profiles. Typically, in contrast, experimentalists use cell culture models to study processes lacking such ideal identity matching. Routinely, perturbation experiments are used to cause controllable genetic defects in networks, which are overall assumed to be intact [46]. However, potential genetic changes, which are ignored, can introduce errors into the interpretation of results. The main scope of the BC-ratio is to provide distances between networks of interest in a cell line and their ideal reference networks. A BC-ratio of 0.0 indicates an unchanged network representing an ideal reference for perturbation experiments. The p_BC_ value evaluates if a BC-ratio > 0.0 was due to chance or it resulted from strong correlation between the nodes with high betweenness centrality and genes mutated in the cell line. Note that a non-significant p_BC_-value does not exclude any functional effects of network changes and it is strongly recommended to collect additional information for evaluating potential functional effects. While our aim was not to predict functional effects related to RNA or protein abundance, we are convinced that the provided data will support case-specific interpretations of functional network integrity.

A central question of our investigation was whether context (i.e. disease or process)-specific networks are overall changed in SH-SY5Y. The absence of network changes was considered as ideal case for controllable perturbation experiments. In the following paragraphs, we first discuss the integrity of disease-specific networks in SH-SY5Y and secondly, focus on PD in more detail.

The cell line suitability (BC-ratio) metric applied to four neurodegenerative diseases ranked them from those with the largest to the smallest changes in the network. Alzheimer’s disease (AD) was ranked as the highest, followed by Parkinson’s disease (PD), Huntington’s disease (HD) and, finally, Amyotrophic Lateral Sclerosis (ALS). BC-ratios in combination with the p_BC_-values tell us that genes mutated in the cell line are more likely to be involved in the information flow control in AD (p_BC_ < 0.05) network than the same type of genes is in the networks of other diseases . Overall, except for the neuroblastoma, BC-ratios were rather low (max BC-ratio = 0.246), and p_BC_-values were not significant for all but neuroblastoma and Alzheimer’s disease. These results indicate that the networks related to PD, HD and ALS are largely intact in the SH-SY5Y cell line and the latter can be considered a suitable model for perturbation experiments targeting these diseases, while AD might require a special caution. It is also important to bear in mind that none of these disease-specific networks were perfectly intact. Indeed, the BC-ratio for each network was computed as greater than 0.0, which indicates that changes in these networks were detected. A prediction of potential functional effects of these network changes, including a classification in gain or loss of function goes beyond the scope of the BC-ratio but the provided data on RNA and protein abundances can support such interpretations.

Genes from which the networks for the neurodegenerative diseases and neuroblastoma have been grown, were derived from the OMIM databse where they have been classified as having disease causative effect (See Methods for the details). We also calculated the BC-ratios of the respective networks assuming that only these seed genes have had been damaged in the cell line. In all cases the number of mutated genes decreased while the BC-ratios of the cell lines increased for all but neuroblastoma (Table 4). Note that in the case of neuroblastoma, the main actors which contributed to the high BC-ratio computed on the real data, already were OMIM genes, damaged in the cell line. Therefore a lower BC-ratio for neuroblastoma was expected in the absence of any other damaged genes. The BC-ratio of the Huntington’s disease increased considerably. This is mostly due to the gene *HTT* followed by *PRNP*, which have the highest betweenness centrality in the HD network. Alzheimer’s disease scores next with *APP* gene making the main contribution to the magnitude of the BC-ratio, being the first on the list of all genes. It is immediately followed by 6 more OMIM-derived genes while their betweenness centrality is more than two times smaller. Similar effect can be observed in the case of Amyotrophic Lateral Sclerosis: *SOD1* has the highest betweenness centrality, followed by *VCP* and *DCTN*1 with almost twice as much lower betweenness. In this experiment, Parkinson’s disease had the lowest BC-ratio although the list was dominated by 5 OMIM-derived genes. The explanation might lie in the low betweenness centralization of the network (Table 2). Network centralization [47] reflects how much variation is there in the centrality scores (in our case, betweenness centrality) among the nodes. The value of 0.246 indicates that the scores are rather equally distributed among the nodes and there are no nodes with apparent brokering role. Therefore changing the node labels from intact to damaged in such network produces smaller effect than in the remaining diseases.

**Table 4:** **BC-ratios of the neurodegenerative diseases and neurobalstoma computed under assumption, that only OMIM-derived seed genes have been mutated**

Disease name	Nodes	Mutated (OMIM genes)	Intact	BC-ratio	p _BC_-value
Huntington’s disease	63	2	61	0.536	<0.001
Alzheimer’s disease	248	13	235	0.495	< 0.001
Neuroblastoma	63	3	60	0.480	0.002
Amyotrophic lateral sclerosis	178	17	161	0.409	< 0.001
Parkinsons’s disease	358	15	343	0.379	< 0.001

The increase in the BC-ratios of all neurodegenerative diseases with the causative OMIM genes being considered as damaged, suggests that the genes which indeed are mutated in the cell line have smaller positional advantages than OMIM genes in the corresponding networks and therefore might less be involved in the information flow control. We believe that this observation supports our conclusion about the overall SH-SY5Y cell line suitability for the experimental studies of the neurodegenerative diseases.

To further analyse the suitability of the cell line as an *in vitro* model for PD, we studied the integrity of disease related sub-networks including mitochondrial dysfunction, reactive oxygen species (ROS) accumulation and calcium homeostasis [48]. Fujita et al. [48] recently provided a comprehensive PD map, which integrates metabolic reactions, gene regulation and signalling processes in this complex network. The network consists of sub-networks (also referred to as modules), which correspond to pathways and processes. In these networks, nodes represent genes, proteins or small molecules, and edges represent molecular interactions.

We selected seven modules corresponding to hallmarks of PD [49] from the PD map and scored them according to the BC-ratio metric. The idea was to segregate PD-related modules as more suitable for studying gain or loss of function of a module. In descending order of BC-ratio, the modules were glycolysis, mitochondria, calcium signalling, apoptosis, dopamine metabolism, ubiquitin proteasome system and reactive oxygen species (ROS) metabolism (Table 3). The P_BC_ values indicated no significant changes in these networks. However, we found that the glycolysis module (Figure 4A) is the most impacted (BC-ratio = 0.31), which suggests that information flow through this module could be significantly altered by the genetic background of SH-SY5Y. One explanation for glycolysis being highly impacted could come from the fact that SH-SY5Y is a cancer cell line. It has been shown that glycolytic rates are higher in cancer cells than healthy ones [50, 51], known as the Warburg effect [51], which has been suggested to confer a proliferative advantage to tumour cells.Conversely, the ROS metabolism module (Figure 4B) was the least impacted (BC-ratio = 0.0), which would indicate that the genes, mutated in the cell line do not compromise the information flow in this network.

For the functional interpretation beyond the BC-ratio, it is important to consider that most of the genetic variations found in the PD modules do not carry mutations, but almost exclusively copy number gains. Indeed, this gain of copy numbers is expected because of the partial trisomy of chromosomes 1, 2, and 17 and the full trisomy of chromosome 3. However, variations that alter the protein sequence occur in very few genes. Among all the damaged genes found in the seven modules from the PD-map, only four contained non-synonymous SNVs. The Sorting Intolerant From Tolerant (SIFT) [52] tool predicted only 3 (*MYO6*, *HADH* and *GZMB*) as damaging (SIFT score < 0.05). The gene *MYO6*, found in the calcium signalling module and involved in intracellular vesicle transport, contains a heterozygous non-synonymous mutation with a SIFT score of 0.01. It also plays an important role in trafficking and activity-dependent recruitment of AMPA receptors to synapses [53]. The gene *HADH*, found in the mitochondria module and which catalyses several reactions in beta-oxidation, also contains a heterozygous non-synonymous mutation with a SIFT score of 0. The gene *GZMB,* found in the apoptosis module and which codes for a serine protease that is used by activated cytotoxic T lymphocytes to induce cell apoptosis [54], contains a rare non-synonymous SNV with a SIFT score of 0.01. However, in each of these three genes, only one copy of the gene contains such rare non-synonymous mutations. Further, four genes (*NDUFA6, TOMM22, ATP5L2)* in the mitochondria module and one gene (*XRCC6*) in the apoptosis module had a copy number of one, leaving only a single working copy. *NDUFA6* has been shown to have a high degree of nitration and be associated with the oxidative damage to mitochondrial complex I [55]. *TOMM22* plays an important role in mitochondrial clearance controlled by *PINK1* –*PARK2* pathways [56]. *ATP5L2* is involved in hydrogen ion trans-membrane transport activity [57]. *XRCC6* codes for the Ku70 protein, which repairs double bond breakage of DNA through the non-homologous end-joining pathway. Therefore, one should consider the damage to these genes when designing loss of function perturbation experiments. Indeed, it is important to test if the mutations per se cause a loss of function and to keep in mind that a function which is already lost cannot be lost a second time. Except for the 7 genes discussed above, all the genes in the modules of the PD map were free of translated mutations in SH-SY5Y.

### Genes related to dopamine metabolism in SH-SY5Y

**Figure 5:**
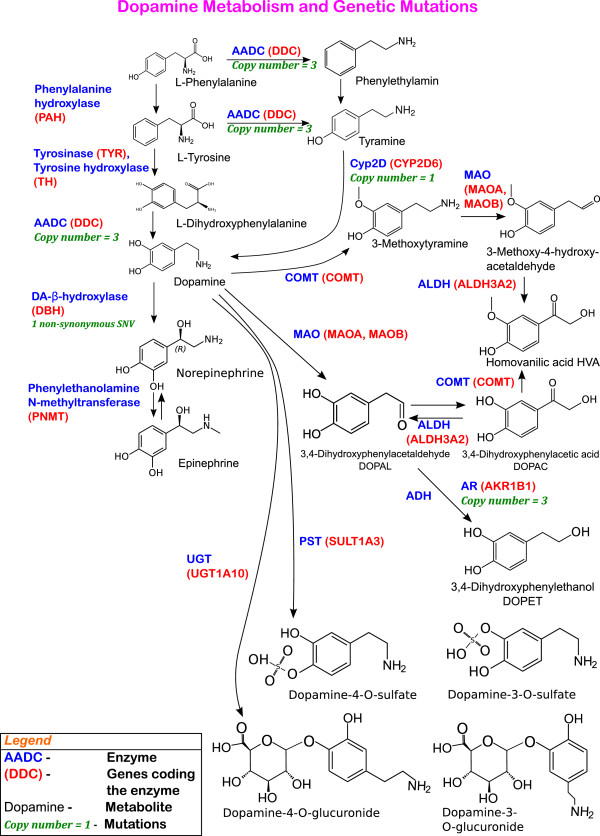
**Dopamine biosynthesis and degradation and the genes coding key enzymes.** This metabolic pathway is a modified version of a figure from Meiser et al. [60]. In addition to the enzymes involved, the genes coding for those enzymes have been added using the HumanCyc [61]. The mutations in SH-SY5Y affecting enzyme-encoding genes have been annotated only if they were rare protein-altering SNVs or indels, CNVs, or SVs.

In summary, all the genes involved in the dopamine metabolism pathway (Figure 5) except *CYP2D6*, *ADH1B* and *UGT1A10* were expressed with or without specific treatments. Further, *CYP2D6*, which contributes to dopamine biosynthesis through an alternative cytochrome P450-mediated pathway shown to exist in rats [58, 59], has a copy number of one, which may contribute to the lower expression. Among the genes in the dopamine metabolism pathway that were expressed with or without specific treatments, only *ADH1C* and *DBH* contained a rare protein-altering mutation and *DDC* and *AKR1B1* had a copy number of three. Only the mutation in *ADH1C* was predicted as damaging by SIFT. Therefore, the large majority of genes involved in dopamine metabolism contains no genetic damage and can be expressed under certain treatments.

## Conclusions

In summary, we provide here the first whole genome analysis of the SH-SY5Y cell line, which is widely used as a model for various neurological diseases. We characterise the different types of biological information of SH-SY5Y – genomics, transcriptomics and proteomics - and compare the relationships between them in terms of expression levels and variants. The data related to the genome sequencing, RNA sequencing and proteomics can be found at the following URL - http://systemsbiology.uni.lu/shsy5y. Additionally, we also compare the SH-SY5Y genome using two widely used whole genome sequencing platforms and show that using the two platform-specific coverages and sensitivities to different indel sizes supplements each other. Finally, our analysis based on context-specific network integrity ranked the integrity of SH-SY5Y for Parkinson’s disease as higher than for Alzheimer’s disease but lower than for Amyotrophic Lateral Sclerosis and Huntington’s disease. In the context of different PD related sub-systems, the same approach ranked ROS metabolism as the most intact followed by the ubiquitin proteasome system, dopamine metabolism, calcium signalling, mitochondria and glycolysis. Overall, most of the genes belonging to the major Parkinson’s disease pathways and modules were intact in the SH-SY5Y genome. Particularly, each analysed gene related to PD has at least one intact copy in SH-SY5Y. Therefore, somatic mutations do not significantly alter PD-related pathways in SH-SY5Y. Importantly, the systems genomics analysis was performed on the undifferentiated cell line although differentiated cell lines are considered better suited as *in vitro* models for PD. Our novel BC-ratio method for cell line scoring integrates a genomic characterisation of the cell line, core set of disease or process-related genes and a protein-protein interaction network. This method is not restricted to a specific cell line or disease and can be broadly applied. Furthermore, if multiple sequenced cell lines are available, the BC-ratio scores can also be used as a guide to select the cell line best suited for studying a particular disease.

## Methods

### SH-SY5Y cell culture

SH-SY5Y cells were cultured from a passage 23 (P23) vial, purchased directly from ATCC (CRL-2266) (Figure 3). In the first step the cells were amplified during 4 passages for preparing passage 27 (P27) freezing stocks. The cells were furthermore amplified during 5 passages for preparing passage 33 (P33) samples for omics analysis. DNA was extracted with the High Pure PCR Template Preparation Kit (Cat. #11796828001, Roche). The samples sequenced by Complete Genomics and Illumina platforms were of equal passage and derived from the same frozen cell stock. For RNA analysis the cells were lysed in Qiazol buffer and were frozen at -80°C. RNA extraction was performed using the miRNeasy Kit (Qiagen). For protein analysis the cells were washed two times with DPBS and for inhibition of proteases the cells were covered with complete (Roche) solution in ddH_2_O and were frozen at -80°C.

### DNA-Seq library preparation

Identical passage samples were derived from the same freezing stock and have been sequenced by Complete Genomics and Illumina platforms. DNA extraction was performed on P33 cells using the High Pure PCR Template Preparation Kit (Cat. #11796828001, Roche). 1 μg of input DNA was sheared using the Covaris to an insert size of 100–900 bp. Sheared DNA was end-repaired by adding End Repair Mix (Illumina) and incubated at 30°C for 30 min. Following end-repair, AMPure XP Beads (Agencourt) were diluted with water and mixed with the DNA samples. The samples were incubated at room temperature for 15 min then placed on a magnetic stand for 15 min. The supernatant was removed and discarded. The beads were washed with 80% ethanol twice, then allowed to air-dry for 15 min. The dried pellet was resuspended in Resuspension Buffer to elute the DNA. The 3′ ends were Adenylated by adding A-Tailing Mix (Illumina) and incubated at 37°C for 30 min. Ligation Mix and a barcoded DNA Adapter was added to each sample and incubated at 30°C for 10 min. The reaction was stopped by adding Stop Ligation Buffer. The ligated DNA was cleaned up twice using undiluted AMPure XP Beads and eluted in Resuspension Buffer. PCR Primer Cocktail and PCR Master Mix were added to the ligated DNA and PCR was performed with the following settings: 98°C for 30 s incubation followed by 10 cycles of 98°C for 10 s, 60°C for 30 s, 72°C for 30 s, final incubation at 72°C for 5 min, hold at 10°C. The PCR reaction was cleaned up with AMPure XP Beads and the library was resuspended in Resuspension Buffer.

The genomic DNA was sequenced by Illumina using the Illumina Fast Track Services (FTS) methodology:

1. gDNA Quantitation.

Genomic DNA is quantified prior to library construction using PicoGreen (Quant-iT™ PicoGreen dsDNA Reagent, Invitrogen, Catalog #: P11496). Quants are read with Spectromax Gemini XPS (Molecular Devices).

2. Library Construction—PCR-Free.

Paired-end libraries are manually generated from 500 ng–1ug of gDNA using the Illumina TruSeq DNA Sample Preparation Kit (Catalog #: FC-121-2001), based on the protocol in the TruSeq DNA PCR-Free Sample Preparation Guide. Pre-fragmentation gDNA cleanup is performed using paramagnetic sample purification beads (Agencourt AMPure XP reagents, Beckman Coulter). Samples are fragmented and libraries are size selected following fragmentation and end-repair using paramagnetic sample purification beads, targeting 300 bp inserts. Final libraries are quality controlled for size using a gel electrophoretic separation system and are quantified.

3. Clustering and Sequencing—v3 Chemistry.

Following library quantitation, DNA libraries are denatured, diluted, and clustered onto v3 flow cells using the Illumina cBot™ system. cBot runs are performed based on the cBot User Guide, using the reagents provided in Illumina TruSeq Cluster Kit v3. Clustered v3 flow cells are loaded onto HiSeq 2000 instruments and sequenced on 100 bp paired-end, non-indexed runs. All samples are sequenced on independent lanes. Sequencing runs are performed based on the HiSeq 2000 User Guide, using Illumina TruSeq SBS v3 Reagents. Illumina HiSeq Control Software (HCS) and Real-Time Analysis (RTA) used on HiSeq 2000 sequencing runs for real-time image analysis and base calling.

### RNA extraction

P33 cells were detached using Trypsin (Cat. #25300, Gibco) and after centrifugation, the cell pellet was lysed in QIAzol buffer and frozen at -80°C. The cell pellet in QIAzol was thawed. Chloroform was added and the sample was centrifuged at 12000 g for 15 min at 4°C. The upper aqueous layer was removed and 1.5 times the volume of the aqueous layer of ethanol was added. RNA in the aqueous layer was bound to an RNeasy Mini column by loading the aqueous layer + ethanol to the top of the column and spinning at 8000 g for 15 s at room temperature. The column was washed once with Buffer RWT, then twice with Buffer RPE. RNA was eluted from the column with 2 washes of 30 μL of water.

### Strand-specific RNA-Seq library preparation

PolyA RNA was isolated from 500 ng of input RNA using oligo(dT)25 Dynabeads (Invitrogen) by binding the RNA to the Dynabeads, collecting the beads with a magnetic rack, and removing the supernatant containing non-polyA RNA. The Dynabeads were washed in washing buffer then the mRNA was eluted in TE buffer. The binding and washing steps were repeated to remove remaining non-polyA RNA. After the binding and washing, the Dynabeads were resuspended in 2× Superscript III first-strand buffer with 10 mM DTT and incubated at 94°C for 6 min to fragment the mRNA then immediately cooled on ice. The Dynabeads were collected on a magnetic stand and the fragment mRNA was moved to a new tube.

Fragmented mRNA in 2× RT buffer, random hexamers, and RNasin Plus was heated at 50°C for 1 min to denature the RNA then placed immediately on ice. To this, water, Actinomycin D, DTT, dNTPs, and SuperScript III (Invitrogen) were added and reverse transcription was performed by incubating the mixture at 25°C for 10 min then at 50°C for 50 min. RNAClean XP beads (Agencourt) were added to the mixture and incubated on ice for 15 min. The beads were collected on a magnetic stand, washed twice with 75% ethanol then air-dried for 5 min. The RNA/cDNA hybrid was eluted in water.

A second strand reaction master mix including either 10× Blue Buffer or NEBuffer 2, dNTP (with dATP, dCTP, dGTP, and dUTP), RNase H, DNA polymerase I (Enzymatics), and water was added to each sample and incubated at 16°C for 2.5 h. Double-stranded DNA was purified using 1.8 volumes of AMPure XP beads (Agencourt) and eluted in water.

The DNA was dA-tailed by adding either 10× Blue Buffer or NEBuffer 2, dATP, water, and Klenow 3′-5′ exo (Enzymatics) and incubated at 37°C for 30 min. The dsDNA was purified using AMPure XP beads, and eluted in water. To each sample, a specific barcode adapter was added, along with Rapid Ligation Buffer and T4 DNA Ligase HC (Enzymatics). The Y-shape adapter ligation was performed by incubating at room temperature for 15 min.

DNA was purified and size selected by cleaning up with AMPure XP beads 3 consecutive times and eluting in TE buffer. The first cleanup was performed with 1 volume of AMPure XP beads, the second with 1.4 volumes, and the last with 1 volume incubated at room temperature for 5 min without washing to perform size selection. The supernatant was transferred to a tube with half of the new volume of AMPure XP beads and incubated at room temperature for 15 min. The beads were collected on a magnetic stand and washed twice with 75% ethanol. The DNA was eluted in water.

The second strand of DNA was digested with Uracil DNA Glycosylase (Enzymatics) at 37°C for 15 min. The PCR reaction was set up containing UDG-digested DNA, primer A and B, Phusion HF Buffer, dNTP, water, and Phusion II (New England Biolabs). The reaction was incubated at 94°C for 2 min, followed by 10–12 cycles of amplification (98°C for 10 s, 65°C for 30 s, 72°C for 30 s). The DNA libraries were purified with 1.4 volumes of AMPure XP beads and eluted in TE buffer.

### Proteomics library preparation

P33 cells were washed twice with DBPS and covered with a 1× complete protease inhibitor cocktail diluted in ddH_2_O and frozen at -80°C. Refer to Additional file 8 for complete details on the proteomic sample preparation and liquid chromatography – mass spectrometry (LC-MS/MS) analysis.

### Whole genome sequencing

SH-SY5Y paired-end whole genome sequencing was done at Illumina and Complete Genomics (CG), Inc (Mountain View, CA) (CG). Whole-genome sequencing was performed (CG) using their proprietary sequencing-by-ligation technology [10]. CG performed primary data analysis using CGAtools v2.0.2.10 including image analysis, base calling, alignment and variant calling. Illumina primary data analysis was performed using CASAVA pipeline v1.8. For both sequencings reads were mapped against the human reference genome (hg19, NCBI build 37).

For CG data, coverage statistics were derived from coverage and coverageRefScore files for each chromosome. Coverage at every base was assessed directly from these files. The depth of coverage roughly follows a normal distribution with significant low and high tails, and has very high sequence-specific local fluctuation. This fluctuation mainly relates to G + C content and is consistent among genomes analyzed with similar versions of CGAtools. We corrected the sequencing coverage observed in CG data by comparing to the coverage observed in 590 genomes available internally at the Institute for Systems Biology, produced with comparable versions of the same technology. To do this, we computed the average coverage level in each 1 kb bin in the 590 reference genomes, after scaling each genome to the geometric average of total autosomal coverage, stratified by G + C content. We then normalised the coverage levels in each 1 kb window of SH-SY5Y to the corresponding median value observed in the reference genome set. For Illumina, coverage information was extracted directly from BAM (binary sequence alignment format) files. To compare CNVs detected with results in literature, CNV measurements of SH-SY5Y using CGH arrays [36] were converted from hg17 to hg19 coordinates (Additional file 3) using the LiftOver tool [62].

For CG, SNVs were derived from the var file. For Illumina, SNVs were extracted from CASAVA output files. SNVs from both platforms were combined into CG testvariant format and compared using custom perl/python scripts. ANNOVAR [21] was used to annotate the SNVs with gene annotations downloaded from the UCSC browser [23] (http://www.genome.ucsc.edu/).

Small insertions and deletions were derived for CG from the VAR file. Indels for Illumina were obtained from CASAVA output files. Copy number variation (CNV) events were taken from cnvSegmentsNondiploidBeta-* file and high-confidence structural variation (SV) events were taken from /highConfidenceSvEventsBeta-* file. The mobile element insertion (MEI) regions were found from mobileElementInsertionsBeta-* file.

### Transcriptome analysis

RNA-Seq FASTQ files were quality trimmed using the novoalign (http://www.novocraft.com) tool with the –a parameter. Sequencing reads were aligned to the UCSC Homo sapiens reference genome hg19 using TopHat v2.0.8 [63, 64], which is integrated with Bowtie v2.0.5 [65] as mapping tool. TopHat removes a small number of reads based on read quality and then maps the reads to a provided reference genome sequence. The pre-built UCSC H. sapiens hg19 bowtie2 index as well as the Ensembl GRCg37 were downloaded from the TopHat Illumina iGenomes site (http://ccb.jhu.edu/software/tophat/igenomes.shtml). TopHat were run with default settings: maximal 40 alignments per read were allowed, with up to 2 mismatches per alignment. Additionally, the flags ‘no-novel-juncs’ with library-type ‘fr-firststrand’ were used to suppress the prediction of new junction sites and to ensure stranded alignments, respectively. The resulting aligned reads in BAM format were analysed further by Cufflinks v2.0.2 [64] in several ways. Cufflinks assembled the aligned reads into transcripts using the Ensembl gene annotation and reported the expression of those transcripts in Fragments Per Kilobase of exon per Million fragments mapped (FPKM). FPKM is an expression of the relative abundance of transcripts. Small variants (SNPs and indels) were called using SAMtools [38] and BEDtools [66]. Using the BAM file generated with TopHat samtools mpileup (-u –q10), bcftools (view –g) and vcfutils.pl (varFilter) from the SAMtools software suite as well as awk (‘($6 > = 50)’) is used to produce a VCF (Variant Call Format) file filtered for minimum quality score of 50. The resulting VCF file is further filtered for falsely called SNPs near splice sites using the BEDtools package to filter out SNPs within a 5 nt window around known splice site junctions.

### Proteomics analysis

A FASTA protein sequence database was created by combining two FASTA databases – (i) protein sequences from the human genome reference hg19 and (ii) protein sequences modified by genomic variants - SNVs, short indels and block substitutions. To merge the two FASTA files, both have been concatenated and redundance has been reduced by cd-hit (parameters: -c 1 –I 0 –G 1 –p 1 –I 6 –d 200 –S 0 –M 16000). If a protein sequence occurred repeatedly, their names were grouped and assigned with a “#” symbol as separator. This approach allowed to track if an identified protein by MaxQuant [67] is solely based on the additional genomic information.

Secondly, the acquired LC-ESI MS/MS data were searched with MaxQuant (v. 1.3.0.5) against the combined FASTA protein database. Default parameters on MaxQuant for unlabelled data were used. Oxidation (M) and N-acetylating have been considered as variable modifications as well as carbamidomethylation on cysteine as a fixed modification. Two missed cleavage sites (Trypsin/P) were allowed and a mass tolerance of 20 ppm of HCD spectra as well as 0.5 Da for CID spectra has been allowed. MS1 precursor mass tolerance was also left at 20 ppm for the first search and 6 ppm for the main search.

The iBAQ [43] method has been used to roughly estimate the absolute quantities of the identified proteins. Since we did not use any spike-in, iBAQ was run without the “log fit” option and the resulting values will just reflect the ordering of the amounts of (and the relative difference between two different proteins in the same run) rather than the absolute quantities in an analytical meaning.

### Diseases related to SH-SY5Y using text mining

The goal of literature analysis was to identify diseases that have been studied using the cell line and or its derivatives. For the purpose of corpus construction we first searched the PubMed collection of abstracts from MEDLINE (http://www.ncbi.nlm.nih.gov/pubmed), PubMed Central (PMC), which is a free full-text archive of biomedical and life sciences journals (http://www.ncbi.nlm.nih.gov/pmc/), and Elsevier repository for articles that mentioned SH-SY5Y cell line (case and dash insensitive) or its spelling variations (e.g., “sh-sy5y cells”) Next, we selected the publications that mentioned the cell line in the title, abstract, list of keywords, section headings, or table/figure captions. These are cues that help to identify principle aspects of article’s content [68, 69]. We executed the above searches with Biopython tools [70] which allowed accessing and querying of PubMed repository, and PMC online facilities to search through the PMC collection. In addition, we implemented a full text parser of the Elsevier articles using the lxml.etree library [71].

After removal of duplicates, items without either title or authors, and items with less than six full sentences (an approximate length of title and abstract), we obtained a collection of 5,353 abstracts and full text articles that dealt with SH-SY5Y cell line. We used Reflect annotation software [72] to identify disease names in the text. Applying heuristic rules described above, we labelled articles with the corresponding disease name, and ranked diseases by their collection frequency.

### Generation of disease networks

Our approach to the cell line evaluation as *in vitro* model relies on the understanding of diseases as genetic network perturbations [73, 74], which requires considering not only single genes of interest but also their role in a complex pathogenic process. For this reason, we represent diseases and process of interest as networks in which nodes are genes (or proteins) and edges are protein-protein interactions. Note that when we scored SH-SY5Y with regard to the Parkinson’s disease modules, we extracted relevant sub-networks directly from the PD map. Alternatively, disease-related networks were built using Online Mendelian Inheritance in Man (OMIM) [75]– a catalogue of human genes and genetic disorders; and STRING – a state-of-the-art database of known and predicted protein-protein interactions [76] version 9.0, from which we extracted human-related undirected network with 18600 nodes and 1640707 edges. In the experiments described here we considered interactions of all types, provided their confidence score was > = 0.7, classified as “high” by the database authors. (See [44] for more details about interactions in STRING.) This reduced the network size to 14688 nodes and 170570 edges. The procedure of disease-related network construction consists of selecting genes described in OMIM as having mutations with causative effect on the disorder; mapping them on the human network, derived from STRING; forming the final network by expanding the core genes with their neighbours at distance one. The choice of the expansion radius is explained by the high average node degree (23.22) of our background STRING–derived network. See Additional file 9 for the list of genes in the network constructed for each disease and module and the list of genes taken as damaged in SH-SY5Y.

### Description of the cell line suitability scoring: BC-ratio

Perturbations applied to the networks affected by the genes mutated in the cell line may produce different phenotypic outputs than the same perturbations applied to the intact networks. Therefore we evaluated the adequacy of the cell line by estimating the impact of the mutated genes on the network that represented the process under study. This analysis aims to first evaluate the node’s importance in the network, and next extend the node-wise information to the entire cell line.

Node’s importance is related to positional advantage it has in the network, and is expressed in terms of “centrality”. Different centrality types reflect different positional properties of a node. Node degree is associated with the node’s visibility and its ability to directly communicate with the other nodes [47]. It does not consider indirect connections of a node and therefore can be considered as the measure of its local importance. Closeness is the reciprocal of mean shortest-path distance between a node and all other nodes that can be reached from it. It is interpreted in terms of how fast a node can communicate with the others [77]. Betweenness centrality measures the extent to which a vertex lies on the paths between the others and, as a consequence, the extent to which the node influences the information flow in the network [78]. In yet another view, node’s centrality is a function of centrality of its neighbours [79] so called power centrality. It is close to eigenvector centrality, which is seen as an attempt to identify important nodes with regard to the overall or global structure of the network. Among this wide variety of centrality metrics we choose betweenness centrality for its emphasis of the nodes ability to alter the information flow in a network.

Betweenness centrality of a node expresses how much information flows through that node. Betweenness centrality *g* of node *v* in the network *G* is given by Equation 1,
1

where *σ*_*st*_ is the total number of shortest paths between the nodes *s*, *t* ∈ *G*, and *σ*_*st*_(*v*) is the number of shortest path that go through *v*.

In order to quantitatively assess the cell line suitability, we extended the metric from individual node characteristic to the characteristic of node types. First of all we mapped the genes mutated in the cell line onto the network under study and labelled all the nodes as “*Damaged*” or “*Intact*”. A gene was labelled as “damaged” if it contained a rare non-synonymous or splice-site SNV, indel or block substitution or if it was found inside a region affected by a CNV or a rare SV. A rare SNV, indel or block substitution occurred with less than 5% frequency in 1000 Genomes Project, 6500 Exome Sequencing Project and Complete Genomics 69 Baseline Genomes dataset. A rare SV was one that was never found in the Complete Genomics Baseline Genome dataset. Next we tried to assess the impact of the damaged nodes on the network. We achieved this by comparing the overall betweenness centrality scores of the damaged and intact nodes. We called this metric *BC-ratio μ*, which was formally defined as follows:
2

The value of *μ* is in range [0:1]. The higher is the BC-ratio value the more is the impact of nodes corresponding to the genes mutated in the cell line on the information flow in the network. The P_BC_ values indicate the probability that the calculated BC-ratio resulted from a random distribution of mutated genes in the network.

To test the metric we generated, for each disease and process, 1000 alternative networks for which we preserved the original topology and counts of damaged/intact nodes but randomized label assignments. We scored each randomized network and calculated p_BC_-value – the probability of obtaining higher BC-ratio with the randomized networks than the one we have obtained with the actual data. P_BC_-value > 0.05 indicates that nodes with high betweenness-centrality in the disease/process network do not significantly correlate with the genes mutated in the cell line –null hypothesis. On the contrary, p_BC_-value < 0.05 indicates that nodes with high betweenness-centrality in the disease network do significantly correlate with the genes mutated in the cell line – alternative hypothesis. Validity of the null hypothesis gives us more ground to assume that from the genetic perspective the cell line is an acceptable candidate for modelling of the disease or process. Based on the p_BC_-values for various diseases and processes (Table 2, Table 3), we cannot confidently reject the null hypotheses for all but two diseases, which are neuroblastoma and Alzheimer’s disease.

In addition to the BC-ratio which is based on the betweenness centrality of the nodes, we calculated the SH-SY5Y integrity with the diseases and process in question using degree, closeness and flow (also known as “random walk”) betweenness centralities [78, 80]. The difference between the latter and the betweenness centrality we have been using so far is that it considers contributions from all paths in the network, not only the shortest, although the latter still counts for more. It has been shown that protein-protein interaction networks can efficiently be modelled with the random walk betweenness centrality [81] so we were interested to compare the two measures in our experimental setting. Alternative cell line scoring implied substitution of the shortest path betweenness centrality of the nodes with one of the selected centrality types. Otherwise the procedure remained the same as specified in Equation 2.

Results of the cell line suitability computations using various centrality metrics are given in Tables 2 and 3. Instead of comparing the cell line scoring values directly we compare centrality values of the individual nodes given by the various metrics. We believe that this information lets us get more insight into the agreement between the centrality types. Table 5 shows correlation between the centrality metrics averaged across diseases and processes. Betweenness only moderately correlated with the degree (0.593) and less so with closeness centrality (0.463) while flow betweenness showed relatively high agreement with the degree (0.7). Degree and closeness showed higher intercorrelation than with the betweenness (0.622). Among all metrics, betweenness and flow betweenness centralities showed the highest correlation (0.955). It suggests that in our experiments either metric could have been used for the cell line suitability evaluation. However the absolute values of the cell line suitability would have not been identical (see Tables 2 and 3, columns “BC-ratio” and “Flow BC”) due to the ability of the flow betweenness centrality to highlight nodes which, although not lying on the shortest paths or occurring on more than one of them (hence, having shortest path betweenness centrality equal or close to 0.0), still contribute to the information flow. Table 6 shows gene ranking in the Mitochondria module of the Parkinson’s disease map in various centrality measures. It can be seen that gene’s rank in both betweenness metrics are very close or even identical while much less so with respect to the degree and closeness centrality. Ranking of *PARK2* and *PINK1* tells us that nodes which do not have high degree or closeness may still play an important role in information flow in the network. Alternatively, positions of *PDHA1* and to some less extent that of *DLD* suggest that having high degree and closeness does not necessarily mean to be a broker. Yet, in some other cases all centrality metrics may agree on the node’s importance as can be seen on *SNCA* and *CYCS*. Overall we would conclude that various metrics should not be regarded as mutually exchangeable. Rather one could choose the metric taking into account the positional advantage it emphasizes. Our approach to the cell line scoring was motivated by the assumption that changes in one gene may affect the entire disease or process network. To model this phenomenon, we were looking for a measure that would reflect node’s role in information transmission over the network. This is the reason why we choose betweenness centrality.

**Table 5:** **Correlation between various centrality metrics**

	Centrality			
	Degree	Closeness	Betweenness	Flow betweenness
Degree		0.622	0.593	0.700
Closeness			0.463	0.592
Betweenness				0.969
Flow betweenness				

The correlation between the different measures of node centrality was calculated for each network. The correlation scores were then averaged over all the networks corresponding to the different diseases and PD modules.

**Table 6:** **Gene scoring agreement across various centrality metrics**

Gene name	Rank according to a centrality metric			
	Degree	Closeness	Betweenness	Flow betweenness
PARK2	24	17	1	1
SNCA	4	4	2	3
PINK1	25	32	3	2
CYCS	3	3	4	4
DLD	1	1	5	5
VDAC1	32	12	6	6
PDHA1	2	2	7	8
SLC25A4	18	5	8	7
BCL2	43	50	9	9
BECN1	42	64	10	13

We show top ten genes from the Mitochondria module of the Parkinson’s.

Disease map, ordered according to the Betweenness centrality rank.

## Electronic supplementary material

Additional file 1: **Co-occurrences of disease terms with the SH-SY5Y cell line in literature.** The first column contains the Human Disease Ontology (HDO) code of the disease, the second column has the disease name and the third column has the number of articles where the disease was mentioned along with the SH-SY5Y cell line. (XLSX 33 KB)

Additional file 2: **Supplementary information.** (DOCX 1 MB)

Additional file 3: **Gene ontology enrichment analysis for private protein-altering SNVs and indels using Biocompendium** [82]**; somatic coding mutations in the TARGET study** [28] **overlapping with mutations in SH-SY5Y.** (XLSX 239 KB)

Additional file 4: **Circos plots for each chromosome of the SH-SY5Y genome.** For each chromosome, tracks represent (from outside to inside) karyotype for each chromosome, copy number variation (red > 2, green = 2, black < 2), density of small variants (bin size = 1 Mb), homozygous small variant percentage (bin size = 1 Mb). Arcs represent chromosomal breakpoints (red = rare breakpoints not found in Complete Genomics Baseline dataset [32]). (PDF 9 MB)

Additional file 5: **Description of 247 different conditions of SH-SY5Y from the GEO database** [42] **for gene expression measurements.** (XLSX 67 KB)

Additional file 6: **Abundance of proteins found in the undifferentiated SH-SY5Y cell line.** The first column contains the protein identifiers (both UCSC and Ensembl formats) identified by MaxQuant using the modified reference database and the second column, the iBAQ score, gives protein abundance as a mean of three different replicates. (XLSX 165 KB)

Additional file 7: **Network visualization of mutated genes in pathways related to Parkinson’s disease.** Each node is a gene and each edge indicates that there is an interaction between them (gene regulatory, protein-protein, metabolic, and signal transduction interactions). Genes annotated with a dark red colour have an exonic mutation, copy number variation or structural variation whereas genes annotated with a light red colour do not have such mutations. (PDF 18 MB)

Additional file 8: **Materials and methods for proteome analysis.** (DOCX 694 KB)

Additional file 9:: **List of genes with rare non-synonymous SNVs, indels, substitutions, copy number variations or structural variations in SH-SY5Y and lists of genes used to construct the networks for cell line suitability scoring.** (XLSX 258 KB)

## Abbreviations

AD: Alzheimer’s disease
ALS: Amyotrophic Lateral Sclerosis
ATCC: American Type Culture Collection
BAM: Binary SAM (Sequence Alignment Format) file
CG: Complete Genomics
CGH: Comparative genomic hybridization
CNS: central nervous system
COSMIC: Catalogue of Somatic Mutations in Cancer
ChIP-Seq: chromatin immune-precipitation sequencing
cDNA: complementary DNA
dbSNP: Single Nucleotide Polymorphism database
DBPS: Dulbecco’s Phosphate Buffered Saline
DMEM: Dulbecco’s Modified Eagle Medium
DNA: deoxyribonucleic acid
ENA: European Nucleotide Archive
FBS: Fetal Bovine Serum
FPKM: Fragments per kilo-base of transcripts per million mapped reads
GATK: Genome Analysis Toolkit
GC/MS: Gas chromatography / mass spectrometry
GEO: Gene Expression Omnibus
HD: Huntington’s disease
HMM: Hidden Markov Model
iBAQ: intensity-based absolute quantification
IL: Illumina
Indel: insertion or deletion
KEGG: Kyoto Encyclopedia of Genes and Genomes
LC-MS/MS: Liquid chromatography – mass spectrometry
M-FISH: Multiplexed fluorescent in-situ hybridization
MEDLINE: Medical Literature Analysis and Retrieval System
OMIM: Online Mendelian Inheritance in Man
PCGP: Pediatric Cancer Genome Project
PD: Parkinson’s disease
PMC: PubMed Central
PPA: Private protein-altering
QC: Quality control
RNA: Ribonucleic acid
RNAseq: RNA sequencing
ROS: Reactive oxygen species
smallRNA: Small ribonucleic acid
SCGC: Sanger Cancer Gene Census
SIFT: Sorting Intolerant From Tolerant
SNV: Single nucleotide variation
Ti-Tv: Transition-transversion
UCSC: University of California, Santa Cruz
VCF: Variant Call Format.

## References

[1] Goncalves J, Warnick S, Iglesias PA, Ingalis BP (2009). System Theoretic Approaches to Network Reconstruction. Control Theory and Systems Biology.

[2] Biedler JL, Roffler-tarlov S, Schachner M, Freedman LS (1978). Multiple neurotransmitter synthesis by human neuroblastoma cell lines and clones. Cancer Res.

[3] Biedler JL, Helson L, Spengler BA (1973). Morphology and growth, tumorigenicity, and cytogenetics of human neuroblastoma cells in continuous culture. Cancer Res.

[4] Xie HR, Hu LS, Li GY (2010). SH-SY5Y human neuroblastoma cell line: in vitro cell model of dopaminergic neurons in Parkinson’s disease. Chin Med J (Engl).

[5] Gilany K, Van Elzen R, Mous K, Coen E, Van Dongen W, Vandamme S, Gevaert K, Timmerman E, Vandekerckhove J, Dewilde S, Van Ostade X, Moens L (2008). The proteome of the human neuroblastoma cell line SH-SY5Y: an enlarged proteome. Biochim Biophys Acta.

[6] Schapira AH (2008). Mitochondrial dysfunction in neurodegenerative diseases. Neurochem Res.

[7] Pahlman S, Ruusala AI, Abrahamsson L, Mattsson ME, Esscher T (1984). Retinoic acid-induced differentiation of cultured human neuroblastoma cells: a comparison with phorbolester-induced differentiation. Cell Differ.

[8] Kovalevich J, Langford D (2013). Considerations for the use of SH-SY5Y neuroblastoma cells in neurobiology. Methods Mol Biol.

[9] Lam HY, Clark MJ, Chen R, Chen R, Natsoulis G, O’Huallachain M, Dewey FE, Habegger L, Ashley EA, Gerstein MB, Butte AJ, Ji HP, Snyder M (2012). Performance comparison of whole-genome sequencing platforms. Nat Biotechnol.

[10] Drmanac R, Sparks AB, Callow MJ, Halpern AL, Burns NL, Kermani BG, Carnevali P, Nazarenko I, Nilsen GB, Yeung G, Dahl F, Fernandez A, Staker B, Pant KP, Baccash J, Borcherding AP, Brownley A, Cedeno R, Chen L, Chernikoff D, Cheung A, Chirita R, Curson B, Ebert JC, Hacker CR, Hartlage R, Hauser B, Huang S, Jiang Y, Karpinchyk V (2010). Human genome sequencing using unchained base reads on self-assembling DNA nanoarrays. Science (New York, NY).

[11] Bentley DR, Balasubramanian S, Swerdlow HP, Smith GP, Milton J, Brown CG, Hall KP, Evers DJ, Barnes CL, Bignell HR, Boutell JM, Bryant J, Carter RJ, Keira Cheetham R, Cox AJ, Ellis DJ, Flatbush MR, Gormley NA, Humphray SJ, Irving LJ, Karbelashvili MS, Kirk SM, Li H, Liu X, Maisinger KS, Murray LJ, Obradovic B, Ost T, Parkinson ML, Pratt MR (2008). Accurate whole human genome sequencing using reversible terminator chemistry. Nature.

[12] Reumers J, De Rijk P, Zhao H, Liekens A, Smeets D, Cleary J, Van Loo P, Van Den Bossche M, Catthoor K, Sabbe B, Despierre E, Vergote I, Hilbush B, Lambrechts D, Del-Favero J (2012). Optimized filtering reduces the error rate in detecting genomic variants by short-read sequencing. Nat Biotechnol.

[13] Sherry ST, Ward MH, Kholodov M, Baker J, Phan L, Smigielski EM, Sirotkin K (2001). dbSNP: the NCBI database of genetic variation. Nucleic Acids Res.

[14] Khurana E, Fu Y, Colonna V, Mu XJ, Kang HM, Lappalainen T, Sboner A, Lochovsky L, Chen J, Harmanci A, Das J, Abyzov A, Balasubramanian S, Beal K, Chakravarty D, Challis D, Chen Y, Clarke D, Clarke L, Cunningham F, Evani US, Flicek P, Fragoza R, Garrison E, Gibbs R, Gümüs ZH, Herrero J, Kitabayashi N, Kong Y, Lage K (2013). Integrative annotation of variants from 1092 humans: application to cancer genomics. Science.

[15] Abecasis GR, Altshuler D, Auton A, Brooks LD, Durbin RM, Gibbs RA, Hurles ME, McVean GA (2010). A map of human genome variation from population-scale sequencing. Nature.

[16] Forbes SA, Bindal N, Bamford S, Cole C, Kok CY, Beare D, Jia M, Shepherd R, Leung K, Menzies A, Teague JW, Campbell PJ, Stratton MR, Futreal PA (2010). COSMIC: mining complete cancer genomes in the Catalogue of Somatic Mutations in Cancer. Nucleic Acids Res.

[17] Adey A, Burton JN, Kitzman JO, Hiatt JB, Lewis AP, Martin BK, Qiu R, Lee C, Shendure J (2013). The haplotype-resolved genome and epigenome of the aneuploid HeLa cancer cell line. Nature.

[18] Meyer M, Kircher M, Gansauge MT, Li H, Racimo F, Mallick S, Schraiber JG, Jay F, Prufer K, de Filippo C, Sudmant PH, Alkan C, Fu Q, Do R, Rohland N, Tandon A, Siebauer M, Green RE, Bryc K, Briggs AW, Stenzel U, Dabney J, Shendure J, Kitzman J, Hammer MF, Shunkov MV, Derevianko AP, Patterson N, Andrés AM, Eichler EE (2012). A high-coverage genome sequence from an archaic Denisovan individual. Science.

[19] **Exome Variant Server** [http://evs.gs.washington.edu/EVS/]

[20] Altshuler DM, Gibbs RA, Peltonen L, Altshuler DM, Gibbs RA, Peltonen L, Dermitzakis E, Schaffner SF, Yu F, Peltonen L, Dermitzakis E, Bonnen PE, Altshuler DM, Gibbs RA, de Bakker PI, Deloukas P, Gabriel SB, Gwilliam R, Hunt S, Inouye M, Jia X, Palotie A, Parkin M, Whittaker P, Yu F, Chang K, Hawes A, Lewis LR, Ren Y, Wheeler D (2010). Integrating common and rare genetic variation in diverse human populations. Nature.

[21] Wang K, Li M, Hakonarson H (2010). ANNOVAR: functional annotation of genetic variants from high-throughput sequencing data. Nucleic Acids Res.

[22] Pruitt KD, Tatusova T, Klimke W, Maglott DR (2009). NCBI reference sequences: current status, policy and new initiatives. Nucleic Acids Res.

[23] Fujita P, Rhead B, Zweig AS, Hinrichs AS, Karolchik D, Cline MS, Goldman M, Barber GP, Clawson H, Coelho A, Diekhans M, Dreszer TR, Giardine BM, Harte RA, Hillman-Jackson J, Hsu F, Kirkup V, Kuhn RM, Learned K, Li CH, Meyer LR, Pohl A, Raney BJ, Rosenbloom KR, Smith KE, Haussler D, Kent WJ (2011). The UCSC genome browser database: update 2011. Nucleic Acids Res.

[24] Flicek P, Ahmed I, Amode MR, Barrell D, Beal K, Brent S, Carvalho-Silva D, Clapham P, Coates G, Fairley S, Fitzgerald S, Gil L, García-Girón C, Gordon L, Hourlier T, Hunt S, Juettemann T, Kähäri AK, Keenan S, Komorowska M, Kulesha E, Longden I, Maurel T, McLaren WM, Muffato M, Nag R, Overduin B, Pignatelli M, Pritchard B, Pritchard E (2013). Ensembl 2013. Nucleic Acids Res.

[25] Harrow J, Frankish A, Gonzalez JM, Tapanari E, Diekhans M, Kokocinski F, Aken BL, Barrell D, Zadissa A, Searle S, Barnes I, Bignell A, Boychenko V, Hunt T, Kay M, Mukherjee G, Rajan J, Despacio-Reyes G, Saunders G, Steward C, Harte R, Lin M, Howald C, Tanzer A, Derrien T, Chrast J, Walters N, Balasubramanian S, Pei B, Tress M (2012). GENCODE: the reference human genome annotation for The ENCODE Project. Genome Res.

[26] Molenaar JJ, Koster J, Zwijnenburg DA, van Sluis P, Valentijn LJ, van der Ploeg I, Hamdi M, van Nes J, Westerman BA, van Arkel J, Ebus ME, Haneveld F, Lakeman A, Schild L, Molenaar P, Stroeken P, van Noesel MM, Ora I, Santo EE, Caron HN, Westerhout EM, Versteeg R (2012). Sequencing of neuroblastoma identifies chromothripsis and defects in neuritogenesis genes. Nature.

[27] Sim NL, Kumar P, Hu J, Henikoff S, Schneider G, Ng PC (2012). SIFT web server: predicting effects of amino acid substitutions on proteins. Nucleic Acids Res.

[28] Pugh TJ, Morozova O, Attiyeh EF, Asgharzadeh S, Wei JS, Auclair D, Carter SL, Cibulskis K, Hanna M, Kiezun A, Kim J, Lawrence MS, Lichenstein L, McKenna A, Pedamallu CS, Ramos AH, Shefler E, Sivachenko A, Sougnez C, Stewart C, Ally A, Birol I, Chiu R, Corbett RD, Hirst M, Jackman SD, Kamoh B, Khodabakshi AH, Krzywinski M, Lo A (2013). The genetic landscape of high-risk neuroblastoma. Nat Genet.

[29] Cheung NK, Zhang J, Lu C, Parker M, Bahrami A, Tickoo SK, Heguy A, Pappo AS, Federico S, Dalton J, Cheung IY, Ding L, Fulton R, Wang J, Chen X, Becksfort J, Wu J, Billups CA, Ellison D, Mardis ER, Wilson RK, Downing JR, Dyer MA (2012). Association of age at diagnosis and genetic mutations in patients with neuroblastoma. JAMA.

[30] Stephens PJ, Greenman CD, Fu B, Yang F, Bignell GR, Mudie LJ, Pleasance ED, Lau KW, Beare D, Stebbings LA, McLaren S, Lin ML, McBride DJ, Varela I, Nik-Zainal S, Leroy C, Jia M, Menzies A, Butler AP, Teague JW, Quail MA, Burton J, Swerdlow H, Carter NP, Morsberger LA, Iacobuzio-Donahue C, Follows GA, Green AR, Flanagan AM, Stratton MR (2011). Massive genomic rearrangement acquired in a single catastrophic event during cancer development. Cell.

[31] Grady DL, Ratliff RL, Robinson DL, McCanlies EC, Meyne J, Moyzis RK (1992). Highly conserved repetitive DNA sequences are present at human centromeres. Proc Natl Acad Sci U S A.

[32] **Complete Genomics Baseline Genome Set** [ftp2.completegenomics.com/Baseline_Genome_Set/CNVBaseline/]

[33] Chu EC, Tarnawski AS (2004). PTEN regulatory functions in tumor suppression and cell biology. Med Sci Monit.

[34] **Complete Genomics Data File Formats** [http://media.completegenomics.com/documents/DataFileFormats_Standard_Pipeline_2.5.pdf]

[35] Yusuf M, Leung K, Morris KJ, Volpi EV (2013). Comprehensive cytogenomic profile of the in vitro neuronal model SH-SY5Y. Neurogenetics.

[36] Do JH, Kim IS, Park T-k, Choi D-k (2007). Molecules and genome-wide examination of chromosomal aberrations in neuroblastoma SH-SY5Y cells by array-based comparative genomic hybridization. Mol Cells.

[37] Leinonen R, Akhtar R, Birney E, Bower L, Cerdeno-Tarraga A, Cheng Y, Cleland I, Faruque N, Goodgame N, Gibson R, Hoad G, Jang M, Pakseresht N, Plaister S, Radhakrishnan R, Reddy K, Sobhany S, Ten Hoopen P, Vaughan R, Zalunin V, Cochrane G (2011). The European nucleotide archive. Nucleic Acids Res.

[38] Li H, Handsaker B, Wysoker A, Fennell T, Ruan J, Homer N, Marth G, Abecasis G, Durbin R (2009). The sequence alignment/Map format and SAMtools. Bioinformatics (Oxford, England).

[39] DePristo MA, Banks E, Poplin RE, Garimella KV, Maguire JR, Hartl C, Philippakis AA, del Angel G, Rivas MA, Hanna M, McKenna A, Fennell TJ, Kernytsky AM, Sivachenko AY, Cibulskis K, Gabriel SB, Altshuler D, Daly MJ (2011). A framework for variation discovery and genotyping using next-generation DNA sequencing data. Nat ldots.

[40] O’Rawe J, Guangqing S, Wang W, Hu J, Bodily P, Tian L, Hakonarson H, Johnson E, Wei Z, Jiang T, Wei Z, Wang K, Lyon GJ (2013). Low concordance of multiple variant-calling pipelines: practical implications for exome and genome sequencing. Genome Med.

[41] Piskol R, Ramaswami G, Li JB (2013). Reliable identification of genomic variants from RNA-seq data. Am J Hum Genet.

[42] Barrett T, Wilhite SE, Ledoux P, Evangelista C, Kim IF, Tomashevsky M, Marshall KA, Phillippy KH, Sherman PM, Holko M, Yefanov A, Lee H, Zhang N, Robertson CL, Serova N, Davis S, Soboleva A (2013). NCBI GEO: archive for functional genomics data sets–update. Nucleic Acids Res.

[43] Schwanhausser B, Busse D, Li N, Dittmar G, Schuchhardt J, Wolf J, Chen W, Selbach M (2011). Global quantification of mammalian gene expression control. Nature.

[44] Biryukov M, Antony PM, Krishna A, Trefois C, May P (2014). Evaluation for Cell Line Suitability for Disease Specific Perturbation Experiments. Data Science, Learning by Latent Structures, and Knowledge: 2014.

[45] Domcke S, Sinha R, Levine DA, Sander C, Schultz N (2013). Evaluating cell lines as tumour models by comparison of genomic profiles. Nat Commun.

[46] Lotharius J, Barg S, Wiekop P, Lundberg C, Raymon HK, Brundin P (2002). Effect of mutant alpha-synuclein on dopamine homeostasis in a new human mesencephalic cell line. J Biol Chem.

[47] Freeman LC (1979). Centrality in social networks conceptual clarification. Social Networks.

[48] Fujita KA, Ostaszewski M, Matsuoka Y, Ghosh S, Glaab E, Trefois C, Crespo I, Perumal TM, Jurkowski W, Antony PM, Diederich N, Buttini M, Kodama A, Satagopam VP, Eifes S, Del Sol A, Schneider R, Kitano H, Balling R (2013). Integrating pathways of Parkinson’s disease in a molecular interaction Map. Mol Neurobiol.

[49] Antony PM, Diederich NJ, Kruger R, Balling R (2013). The hallmarks of Parkinson’s disease. FEBS J.

[50] Gatenby RA, Gillies RJ (2004). Why do cancers have high aerobic glycolysis?. Nat Rev Cancer.

[51] Vander Heiden MG, Cantley LC, Thompson CB (2009). Understanding the Warburg effect: the metabolic requirements of cell proliferation. Science.

[52] Kumar P, Henikoff S, Ng PC (2009). Predicting the effects of coding non-synonymous variants on protein function using the SIFT algorithm. Nat Protoc.

[53] Nash JE, Appleby VJ, Correa SA, Wu H, Fitzjohn SM, Garner CC, Collingridge GL, Molnar E (2010). Disruption of the interaction between myosin VI and SAP97 is associated with a reduction in the number of AMPARs at hippocampal synapses. J Neurochem.

[54] Thomas DA, Scorrano L, Putcha GV, Korsmeyer SJ, Ley TJ (2001). Granzyme B can cause mitochondrial depolarization and cell death in the absence of BID, BAX, and BAK. Proc Natl Acad Sci U S A.

[55] Murray J, Taylor SW, Zhang B, Ghosh SS, Capaldi RA (2003). Oxidative damage to mitochondrial complex I due to peroxynitrite: identification of reactive tyrosines by mass spectrometry. J Biol Chem.

[56] Bertolin G, Ferrando-Miguel R, Jacoupy M, Traver S, Grenier K, Greene AW, Dauphin A, Waharte F, Bayot A, Salamero J, Lombès A, Bulteau AL, Fon EA, Brice A, Corti O (2013). The TOMM machinery is a molecular switch in PINK1 and PARK2/PARKIN-dependent mitochondrial clearance. Autophagy.

[57] Maglott D, Ostell J, Pruitt KD, Tatusova T (2011). Entrez Gene: gene-centered information at NCBI. Nucleic Acids Res.

[58] Hiroi T, Imaoka S, Funae Y (1998). Dopamine formation from tyramine by CYP2D6. Biochem Biophys Res Commun.

[59] Bromek E, Haduch A, Golembiowska K, Daniel WA (2011). Cytochrome P450 mediates dopamine formation in the brain in vivo. J Neurochem.

[60] Meiser J, Weindl D, Hiller K (2013). Complexity of dopamine metabolism. Cell Commun Signal.

[61] Romero P, Wagg J, Green ML, Kaiser D, Krummenacker M, Karp PD (2005). Computational prediction of human metabolic pathways from the complete human genome. Genome Biol.

[62] Hinrichs AS, Karolchik D, Baertsch R, Barber GP, Bejerano G, Clawson H, Diekhans M, Furey TS, Harte RA, Hsu F, Hillman-Jackson J, Kuhn RM, Pedersen JS, Pohl A, Raney BJ, Rosenbloom KR, Siepel A, Smith KE, Sugnet CW, Sultan-Qurraie A, Thomas DJ, Trumbower H, Weber RJ, Weirauch M, Zweig AS, Haussler D, Kent WJ (2006). The UCSC genome browser database: update 2006. Nucleic Acids Res.

[63] Trapnell C, Pachter L, Salzberg SL (2009). TopHat: discovering splice junctions with RNA-Seq. Bioinformatics (Oxford, England).

[64] Trapnell C, Roberts A, Goff L, Pertea G, Kim D, Kelley DR, Pimentel H, Salzberg SL, Rinn JL, Pachter L (2012). Differential gene and transcript expression analysis of RNA-seq experiments with TopHat and Cufflinks. Nat Protoc.

[65] Langmead B, Salzberg SL (2012). Fast gapped-read alignment with Bowtie 2. Nat Methods.

[66] Quinlan AR, Hall IM (2010). BEDTools: a flexible suite of utilities for comparing genomic features. Bioinformatics (Oxford, England).

[67] Cox J, Mann M (2008). MaxQuant enables high peptide identification rates, individualized p.p.b.-range mass accuracies and proteome-wide protein quantification. Nat Biotechnol.

[68] Cohen KB, Johnson HL, Verspoor K, Roeder C, Hunter LE (2010). The structural and content aspects of abstracts versus bodies of full text journal articles are different. BMC Bioinformatics.

[69] McDonald D, Chen H (2002). Using Sentence-Selection Heuristics to Rank Text Segments in TXTRACTOR. Management Information Systems.

[70] **Biopython** [http://biopython.org/wiki/Main_Page]

[71] **The lxml.etree Tutorial** [http://lxml.de/tutorial.html]

[72] Pafilis E, O’Donoghue SI, Jensen LJ, Horn H, Kuhn M, Brown NP, Schneider R (2009). Reflect: augmented browsing for the life scientist. Nat Biotechnol.

[73] Zhu X, Gerstein M, Snyder M (2007). Getting connected: analysis and principles of biological networks. Genes Dev.

[74] del Sol A, Balling R, Hood L, Galas D (2010). Diseases as network perturbations. Curr Opin Biotechnol.

[75] McKusick VA (2007). Mendelian inheritance in Man and its online version, OMIM. Am J Hum Genet.

[76] Franceschini A, Szklarczyk D, Frankild S, Kuhn M, Simonovic M, Roth A, Lin J, Minguez P, Bork P, von Mering C, Jensen LJ (2013). STRING v9.1: protein-protein interaction networks, with increased coverage and integration. Nucleic Acids Res.

[77] Wasserman S, Faust K (1994). Social Network Analysis: Methods and Applications.

[78] Newman MEJ (2005). A measure of betweenness centrality based on random walks. Social Networks.

[79] Bonacich P (1987). Power and centrality: a family of measures. Am J Sociol.

[80] Brandes U, Fleischer D (2005). Centrality Measures Based on Current Flow. STACS 2005, Lecture Notes in Computer Science.

[81] Missiuro PV, Liu K, Zou L, Ross BC, Zhao G, Liu JS, Ge H (2009). Information flow analysis of interactome networks. PLoS Comput Biol.

[82] **bioCompendium** [http://biocompendium.embl.de/]

