# Supplementary material for: Systems genomics evaluation of the SH-SY5Y neuroblastoma cell line as a model for Parkinson’s disease
Source: BMC Genomics. 2014 Dec 20;15(1):1154. doi: 10.1186/1471-2164-15-1154 (PMC4367834; doi:10.1186/1471-2164-15-1154)
Supplement: Supplementary file 2 — Additional file 2: Supplementary information. (DOCX 1 MB) [file 12864_2014_6889_MOESM2_ESM.docx]

Supplementary Information

1. S1 | Definitions of concordant and discordant variants 1
2. S2 | Comparison between Complete Genomics and Illumina 2
3. S3 | Coverage of the genome and percentage of genome covered 3
4. S4 | Size distribution of small indels 5
5. S5 | Gene annotation 6
6. S6 | Comparison of SNVs and indels with the HeLa cell line (Adey et al.) 11
7. S7 | Annotation by Catalogue of Somatic Mutations in Cancer (COSMIC) 12
8. S8 | Genes mutated in neuroblastoma 12
9. S9 | Annotation by miRBase 13
10. S10 | SNP detection and intersection 13
11. S11 | Indel detection and intersection 15
12. S12 | Platform-specific calls and non-variant calls in the other platform 17
13. S13 | Mapping of CG substitutions to SNVs and indels 19
14. S14 | Rationale for filtering of variants 20
15. S15 | Structural variations using Complete Genomics 21
16. S16 | Structural variations using Illumina 22
17. S17 | Copy Number Variations (CNV) 24
18. S18 | A reference genome 24
19. S19 | RNA-Seq variant calling 25
20. S20 | SmallRNA variant calling 26
21. S21 | Concordance between RNA-Seq and DNA-Seq 27
22. S22 | Concordance between DNA-Seq and smallRNA sequencing 27
23. S23 | Illumina genotyping 28
24. S24 | Protein abundance 29
25. S25 | Validation of SNVs and short indels using proteomics 29
26. S26 | Genomic mutations and gene expression of SH-SY5Y across 247 conditions 29
27. S27 | Genetic copy number and gene expression across different conditions 30
28. S28 | Comparison of genetic copy number and RNA-Seq expression 30
29. S29 | Comparison of RNA-seq expression and protein abundance of a gene 31
30. S30 | Suitability of the SH-SY5Y cell line for perturbation experiments in the context of modules in the Parkinson’s’ Disease map 33

# Definitions of concordant and discordant variants

In brief, we define concordant variants as those which are found in both platforms with the same genotype, partially concordant as those which are found in both platforms with a different genotype, and discordant as those found in only one platform.

**Details:**

We extend the definition used by Reumers et al. (2011) for shared and discordant SNVs to other variants (indels and substitutions) – discordant variants are those identified in only one of the genomes or when variants exhibited discordant genotypes between the two genomes. Similarly, shared variants are those with the same bi-allelic genotype at a given position.

We introduce another classification, namely partially concordant variants – those identified in one genome as fully called (10, 11 or 01), and in the other genome as a partially called variant (1N but not 0N as there is no evidence of a variant being called).

| **Genotype in platform 1** | | **Genome in platform 2** | | **Concordance class** |
| --- | --- | --- | --- | --- |
| Allele 1 | Allele 2 | Allele 1 | Allele 2 |  |
| 0 | 1 | 0 | 1 | Concordant |
| 0 | 1 | 1 | 0 | Concordant |
| 1 | 1 | 1 | 1 | Concordant |
| 0 | 1 | 0 | 0 | Discordant |
| 0 | 1 | 0 | N | Discordant |
| 1 | 1 | 0 | 0 | Discordant |
| 1 | 1 | 0 | N | Discordant |
| 1 | N | 0 | 0 | Discordant |
| 1 | N | 0 | N | Discordant |
| 0 | 1 | 1 | 1 | Partially concordant |
| 0 | 1 | 1 | N | Partially concordant |
| 1 | 1 | 0 | 1 | Partially concordant |
| 1 | 1 | 1 | 0 | Partially concordant |
| 1 | 1 | 1 | N | Partially concordant |
| 1 | N | 0 | 1 | Partially concordant |
| 1 | N | 1 | 0 | Partially concordant |
| 1 | N | 1 | 1 | Partially concordant |
| 1 | N | 1 | N | Partially concordant |

**Table 1: Definition of concordant, partially concordant and discordant variants.** 0 and 1 refer to the absence and presence of the variant respectively. N means no confident measurement was made at that position.

# Discussion of the comparison between Complete Genomics and Illumina

We judged the overall quality of the variant calls using three metrics - the transition/transversion (ti-tv) ratio, heterozygous/homozygous (het-hom) ratio, and novelty of variants. Transitions are mutations from a purine (adenine or guanine) to another purine or from a pyrimidine (cytosine or thymine) to pyrimidine, whereas transversions are mutations from a purine to a pyrimidine or vice-versa. Human mutations contain roughly twice as many transitions than transversions. Therefore, the ti-tv ratio was expected to be 2.0-2.1 in the cell line, as it is equal to that found in human genomes in the 1000 Genomes Project (Abecasis et al.). Even though the overall ti-tv ratios for both platforms were in the range of 2.0-2.1 (2.09 for CG and 2.03 for IL), the ti-tv ratio for platform-specific variants was lower for both platforms - 1.49 for CG and 1.46 for IL.

The heterozygous-homozygous ratio is the ratio of the number of heterozygous SNVs to homozygous SNVs, which is expected to equal 1.5 from previous sequencing of human genomes (Abecasis et al.). Although the heterozygous/homozygous ratios for both platforms were close to the expected value of 1.5, the ratio for platform specific variants was much higher - 8.637 for CG and 3.285 for IL (Table 9). The percentage of variants found in dbSNP (build 137), 1000 Genomes Project or Exome Sequencing Project was 95.6 %. However, a smaller percentage of platform-specific variants were found in these databases – 50.8 % of CG-specific and 82.2 % of IL-specific variants – indicating that a significant number of them are either genuine, novel somatic mutations or sequencing errors.

Out of a total of 747,234 indels, 101,035 indels were specific to the CG platform and 233,536 were specific to the IL platform (Table 10). Therefore, there was a greater degree of indel discordance compared to SNVs, perhaps because of the sensitivity of indel calling to read-mapping errors. The larger platform-bias related to indels when compared to SNVs was also found in an earlier study comparing CG and IL sequencing platforms (Lam et al.). Also, CG shows a greater enrichment towards 1-bp insertions compared to IL (Additional File 2 - Figure 3). The distribution of IL indels is more skewed towards larger indels compared to CG (Table 4), perhaps reflecting the larger read size used by IL (101 base-pairs) compared to CG (36 base-pairs).

Further, 81 % of IL-specific SNVs were no-call regions for CG and 48 % of CG-specific calls were no-call regions for IL (Figure 14,15). These results indicate that CG is more likely to have not called a region where IL-specific SNVs exist than vice versa. Further around 66 % (*n* = 269,950) of these IL-specific SNVs are in repeat regions. Considering that mapping in these repeat regions is error-prone, CG appears to give fewer false positives. It is notable that only 8 % (*n* = 32,752) of IL-specific SNVs were called as reference by CG, whereas 88 % (*n* = 118,454) of CG-specific SNVs were called as reference by IL.

As for platform-specific indels, 69 % of those specific to IL are inside no-call regions for CG (Additional File 2 - Figure 16). However, only 2.7 % of CG-specific indels were in IL no-call regions and the remaining 97.2 % were called as reference (Figure 17). Therefore, the disagreement in platform-specific indels suggests a bias towards no-calls by CG, but a reference bias in IL.

A third class of small variants – block substitutions – were only called by CG, which could be mapped to SNVs and indels from IL with the loss of haplotype information. Only 9 % of 88,874 block substitutions overlapped with IL no-call regions. 39 % of the block substitutions completely mapped to IL variants, 31 % were called as reference by IL and 21 % were discordant (Figure 18).

All these metrics support the case for a large number of false positives in platform-specific calls. Our approach therefore placed a high confidence on variants shared by both platforms and used their coverage and quality values to drive the variant filtering process (section S14). To ensure a minimal number of false positive SNVs and small indels, we followed the technique and parameters described by Reumers et al. Consequently, we used the concordance between two sequencing platforms and filtered based on (i) coverage in both platforms, (ii) variant quality scores in the two platforms, (iii) regions problematic for read-mapping (segmental duplication, microsatellites, simple repeats, self-chained regions, homo-polymers, proximity to homo-polymer region and proximity of SNVs to indels) and (iv) clustering of SNVs (low likelihood of high density of SNVs). Consequently, the variant filters increased the SNV concordance rate from 84.0 % to 99.3 %, but removed 90.0 % of CG-specific and 99.6 % of IL-specific variant calls (Additional File 2 - Figure 10, 11). Similarly, the filters also increased the indel concordance rate from 47.0 % to 85.5 %, but removed 96.0 % and 94.6 % of CG and IL-specific indels respectively (Additional File 2 - Figure 12, 13).

# Coverage of the genome and percentage of genome covered

Figure 1: Distribution of read depths

More than 90% of genome has >20x coverage

Figure 2: Cumulative distribution over the whole genome

For CG, the genome coverage was calculated by dividing the Gross Mapping Yield in the summary-*.tsv file by the length of the hg19 genome. Here the Gross Mapping Yield is defined as the count of called bases within DNB (DNA-nanoball) arms with at least one initial mapping to the reference genome, excluding reads marked as overflow. (See CG DataFileFormats Standard Pipeline 2.4 for details.) Rest of the values were directly taken from the same file. For Illumina, the values were taken from the build stats.txt.

|  | **Complete Genomics** | **Illumina** |
| --- | --- | --- |
| Genome coverage | 56.69 | 49.20 |
| Gross mapping yield (Gb) | 177.8 | 138.563 |
| Exome coverage | 45.08 | 57.53 |
| Fraction of genome fully called | 0.971 | 0.9823 |
| Fraction of exome fully called | 0.955 | 0.9738 |
| Heterozygous-homozygous ratio | 1.549 | 1.574 |
| Transition-to-transversion ratio (ti/tv) | 2.11 | 2.01 |

**Table 2: Alignment statistics for quality control**

A testvariant file (CG format: 00, 01, 11, 1N, 0N) was generated to compare variants from CG and IL. The alleles for CG were computed using cgatools^[[1]](#endnote-1)^ listvariants and testvariants using the CG var file as input. The values for IL were taken from the variants file from Illumina CASAVA^[[2]](#endnote-2)^ pipeline. Homozygous and heterozygous event counts were done on the sites that were fully called, whereas the transition/transversion ratio was calculated using partially and fully called sites.

|  | **Complete Genomics** | **Illumina** |
| --- | --- | --- |
| Heterozygous-homozygous ratio – SNP | 1.275 | 1.283 |
| Heterozygous-homozygous ratio – INS | 1.400 | 1.247 |
| Heterozygous-homozygous ratio – DEL | 1.489 | 1.494 |
| Heterozygous-homozygous ratio – SUB | 1.819 | No block substitutions |
| Transition/transversion ratio – SNP | 2.097 | 2.032 |

**Table 3: Comparison of metrics for small variants**

# Size distribution of small indels

CG has a greater enrichment towards 1-bp insertions

*Insertions*

*Deletions*

Figure 3: Distribution of sizes of small indels

|  | **CG** | **Illumina** |
| --- | --- | --- |
| **Number of insertions** | 260445 | 340350 |
| **Largest insertion size** | 67 | 300 |
| **Upper quartile** | 2 | 4 |
| **Median** | 1 | 1 |
| **Lower quartile** | 1 | 1 |
| **Smallest insertion size** | 1 | 1 |
| **Mean insertion size** | 2.3 | 5.9 |
|  |  |  |
| **Number of deletions** | 267955 | 340811 |
| **Largest deletion size** | 190 | 300 |
| **Upper quartile** | 3 | 4 |
| **Median** | 1 | 2 |
| **Lower quartile** | 1 | 1 |
| **Smallest deletion size** | 1 | 1 |
| **Mean deletion size** | 2.9 | 5.0 |

**Table 4: Number and size distribution of small indels**

# Gene annotation

Gene annotation was performed on variants using a consensus of four databases - RefSeq refgene (release 55), UCSC knowngene, Ensembl ensgene v65 and GENCODE V4. Each of the different gene annotation databases may have conflicting results for a variant. So, we decided to use the gene labels in the following order of priority (modified from ANNOVAR website - http://www.openbioinformatics.org/annovar/annovar gene.html#output1)

1. exonic

2. splicing

3. ncrna

- ncrna exonic

- ncrna splicing

- ncrna UTR5

- ncrna UTR3

- ncrna intronic

4. UTR5

5. UTR3

6. intronic

7. upstream

8. downstream

9. intergenic

So if a variant is labelled as exonic by refgene and splicing by all other databases, we counted it as exonic. In this way, our method was sensitive to deleterious harmful variants.

Similarly for coding effect annotation, we will use the following priority to integrate the annotations –

1. stop-gain
2. stop-loss
3. missense
4. synonymous
5. frameshift
6. non-frameshift

Figure 4: Percentage association of SNVs over UTR5, UTR3, exonic and intronic regions

Figure 5: Percentage association of SNVs over splicing, ncRNA, upstream and downstream regions

Figure 6: Percentage association of indels and substitutions over UTR5, UTR3, exonic and intronic regions

Figure 7: Percentage association of indels and substitutions over splicing, ncRNA, upstream and downstream regions

Figure 8: Percentage association of SNVs over coding types: synonymous, missense and nonsense

Figure 9: Percentage association of indels and substitutions over coding types: frameshift, non-frameshift, stop-gain and stop-loss

|  | **CG-specific** | | **IL-specific** | | **Partially concordant** | | **Fully concordant** | |
| --- | --- | --- | --- | --- | --- | --- | --- | --- |
|  | SNV | Indel | SNV | Indel | SNV | Indel | SNV | Indel |
| **UTR5** | 824 | 174 | 1399 | 516 | 506 | 120 | 7999 | 686 |
| **UTR3** | 1029 | 978 | 1854 | 1689 | 553 | 539 | 27282 | 3901 |
| **Exonic** | 2052 | 265 | 2814 | 359 | 884 | 57 | 21324 | 436 |
| **Intronic** | 39986 | 41489 | 106029 | 81332 | 24440 | 24207 | 1178675 | 133358 |
| **Intergenic** | 64382 | 42369 | 204351 | 108439 | 38268 | 26958 | 1513508 | 156325 |
| **Splicing** | 27 | 8 | 30 | 20 | 10 | 8 | 238 | 32 |
| **ncRNA** | 22702 | 13428 | 80038 | 35290 | 12838 | 8084 | 457240 | 48701 |
| **Upstream** | 2166 | 1092 | 7220 | 2973 | 1299 | 673 | 31690 | 3508 |
| **Downstream** | 1835 | 1232 | 5672 | 2918 | 961 | 788 | 33930 | 4282 |

**Table 5: Genome annotation of variants in SH-SY5Y cell line**

|  | **CG-specific** | | **IL-specific** | | **Partially concordant** | | **Fully concordant** | |
| --- | --- | --- | --- | --- | --- | --- | --- | --- |
|  | SNV | Indel | SNV | Indel | SNV | Indel | SNV | Indel |
| **UTR5** | 23 | 4 | 3 | 26 | 2 | 2 | 4249 | 264 |
| **UTR3** | 69 | 50 | 14 | 125 | 11 | 6 | 17301 | 1562 |
| **Exonic** | 38 | 4 | 4 | 11 | 2 | 2 | 10603 | 170 |
| **Intronic** | 4400 | 1503 | 629 | 4540 | 380 | 343 | 824232 | 51297 |
| **Intergenic** | 6821 | 1862 | 858 | 5850 | 658 | 431 | 1086811 | 60495 |
| **Splicing** | 3 | 0 | 0 | 0 | 0 | 2 | 153 | 16 |
| **ncRNA** | 1829 | 547 | 262 | 1730 | 156 | 121 | 314131 | 18836 |
| **Upstream** | 145 | 39 | 14 | 107 | 12 | 7 | 19282 | 1169 |
| **Downstream** | 156 | 55 | 33 | 152 | 11 | 17 | 21332 | 1496 |

**Table 6: Genome annotation of filtered variants in SH-SY5Y cell line**

# Comparison of SNVs and indels with the HeLa cell line (Adey et al.)

|  | **SH-SY5Y filtered** | **SH-SY5Y** | **HeLa CCL-2** | **Reich 11^#^** |
| --- | --- | --- | --- | --- |
| Number of SNVs | 2314627 | 3896055 | 4068395 | 4178701 |
| Number of indels | 152841 | 747234 | 417471 | 334885 |
| Number of 1kG SNVs | 2277521 | 3417555 | 3670543 | 3663541 |
| Number of non-1kG SNVs | 37106 | 478500 | 397852 | 515159 |
| Number of 1kG indels | 65217 | 129502 | 195613 | 193522 |
| Number of non-1kG indels | 87624 | 617732 | 221858 | 141363 |
| % SNVs that are homozygous*** | 38.43 | 37.47 | 43.99 | 40.42 |
| Ti/Tv for SNVs in 1 kG | 2.17 | 2.12 | 2.14 | 2.15 |
| Ti/Tv for SNVs not in 1 kG | 1.56 | 1.40 | 1.55 | 1.65 |
| Private SNVs |  | 422441 |  |  |
| Private Protein-Altering (PPA) SNVs* | 88(75) | 2286 (1598)** | 269 | 390.9 |
| PPA SNVs in COSMIC | 4(2) | 123 (79) | 1 | 2.6 |
| PPA SNVs in Cancer Genes | 4(4) | 66 (58) | 4 | 8.7 |
| Private indels |  | 609834 |  |  |
| PPA indels | 79 (34) | 571 (305) | 35 | 17.5 |
| PPA indels in COSMIC | 2(1) | 21 (15) | 0 | 0 |
| PPA indels in Cancer Genes | 5(3) | 16 (10) | 1 | 0.1 |

* Private variants are those variants that are not found in 1000 Genomes Project (1kG) or the Exome Sequencing Project 6500 call set, and found outside regions annotated for excessive sequence depth (HiSeq top 5%ile coverage track from the UCSC genome browser). They are protein altering if they are annotated as protein-sequence altering using gene annotation based on integrating annotation from Ensembl, Refseq, UCSC and Gencode. All of these variants were also called with a minimum coverage of 8X by at least one of the platforms (CG or IL).

** Values inside brackets correspond to “protein-altering” variants as annotated by NCBI Refseq and CCDS gene annotation databases, which are used by SeattleSeq Annotation Server.

*** SNVs that were called homozygous by one of the platforms (CG or IL) and the genotype called by the other platform was neither homozygous reference or heterozygous.

^#^Reich 11 refers to the average values for 11 Human Genome Diversity Project (HGDP) control individuals from Meyer et al.

**Table 7: Number of variants and annotations compared to HeLa cell line**

The 4 filtered PPA SNVs that are present in COSMIC are in the genes KAT6A, MED12, MPL and SMARCA4. Mutations in SMARCA4 can cause rhabdoid tumour predisposition syndrome type 2 (OMIM).

| Number of genes in Sanger Cancer Gene Census (SCGC) | 506 |
| --- | --- |
| Number of those genes with corresponding Refseq IDs | 495 |
| Number of Refseq IDs mapped | 1417 |
| Number of RefSeq IDs that have a genomic location | 1225 |

**Table 8: Number of RefSeq gene ids mapped from Sanger Cancer Gene Census**

# Annotation by Catalogue of Somatic Mutations in Cancer (COSMIC)

COSMIC version 64 contains 696,932 mutations, each with an ID of the form COSMdddddd (where d is a digit), and the primary tissue type where the mutation was found. 4,336 allele-specific mutations in SH-SY5Y were found in COSMIC.

# Genes mutated in neuroblastoma

The genome sequencing of SH-SY5Y found 27 genes with rare (< 5% frequency in 1000 Genomes Project, 6500 Exome Sequencing Project and Complete Genomics 69 Baseline Genomes dataset) non-synonymous SNVs, indels or substitutions and 2 genes with splice-site mutations that overlapped with the list of 586 genes containing somatic mutations in the complete genome sequence of 87 untreated primary neuroblastoma tumours. The list of those genes is given below. The genes that contained mutations predicted by SIFT as damaging (score <0.05) are written in bold.

| CARS2* | **GPRC6A** | **ODZ4*** | **UGT2B7** |
| --- | --- | --- | --- |
| **DNAH3** | HMCN1 | **OSGIN1** | **VPS37A*** |
| **DNAH9** | **MKI67*** | PCDHA5 | **ZFP57** |
| **DNAI1** | **MMP12** | PCSK5 |  |
| **ENPP1** | MS4A14 | **PGK2** |  |
| FRAS1* | MYH13 | **TAGAP** |  |
| **GABRR3** | **NBN*** | THSD7A* |  |
| GAK* | **NSMAF*** | TIAM1* |  |

*Genes with mutations that were also found in RNA-seq data. The remaining mutations that were not detected by RNA-seq resided in genes with FPKM < 1.

# Annotation by miRBase

miRBase Release 19 contains predicted hairpin portions of 3828 human miRNAs. 179 SNVs and short indels were inside the predicted hairpin loop portion of miRNAs.

# SNP detection and intersection

|  | Number of SNPs*** | Ti/tv ratio* | Het/hom ratio** | Found in dbSNP (build 132) and COSMIC | Found in dbSNP (build 137), 1kGP or ESP 6500 |
| --- | --- | --- | --- | --- | --- |
| **CG:** |  |  |  |  |  |
| Total | 3486648 | 2.09 | 1.548 | 3262988 (93.6 %) | 3386499 (97.1 %) |
| Specific | 135003 | 1.49 | 8.637 | 64086 (47.5 %) | 68544 (50.8 %) |
| **Illumina:** |  |  |  |  |  |
| Total | 3761052 | 2.03 | 1.482 | 3198902 (85.05%) | 3654572 (97.2 %) |
| Specific | 409407 | 1.46 | 3.285 | 0 (0 %) | 336617 (82.2 %) |
| **Overall:** |  |  |  |  |  |
| Total | 3896055 | 2.01 | 1.623 | 3262988 (83.8 %) | 3723116 (95.6 %) |
| Fully concordant | 3271886 | 2.13**** | 1.482 | 3128923 (95.6 %) | 3239422 (99.0 %) |
| Partially concordant | 79759 | 1.65 | 0.988 (CG)  0.508 (Illumina) | 69979 (87.7 %) | 78533 (98.5 %) |

**Table 9: SNP call-set metrics for concordant and discordant SNPs**

*The **ti/tv ratio** was calculated by counting the positions where there is a transition or a transversion called. I have not counted 1 transition per allele (but 1 transition per position) as otherwise, the partially concordant class would further have a different ti/tv ratio for each platform.

**The **het/hom ratio** was calculated by counting all positions where an SNV is called, and the platform is fully called at that position. If 2 variants at the same position are such that both are heterozygous, the position is counted only once.

The overall het/hom ratio is calculated by using the number of unique heterozygous (or homozygous) events in the union of variants from both platforms. So,

Total number of heterozygous events = het(CG-specific)+het(IL-specific) + het(fully concordant) + het(CG-partially concordant) + het(IL-concordant)

where het(.) is the number of heterozygous variants in a concordance class.

***Note: The platform-specific and concordant SNP positions will not add up to, but will be fewer than the total number of SNPs as the they do not include positions at which one of the platforms is not fully called, whereas the other identifies a SNV (we have not included this among discordant SNVs)

The total number of SNVs overall = CG-specific + IL-specific + fully concordant + partially concordant

****The overall total transition (or transversion) was calculated as the sum of transitions (or transversions) of CG-specific, IL-specific, fully concordant and partially concordant SNVs.

Platform specific SNVs are those where both platforms have fully-called both alleles and the bi-allelic genotype call for both platforms is different (e.g. homozygous vs. heterozygous).

Concordant SNVs are those where neither of the alleles is a no-call and the bi-allelic genotype call for both the platforms is the same.

135003

3351645 (3271886+79759)

***CG***

***Illumina***

409407

***CG+IL***

**Figure 10: Intersection of SNPs between Complete Genomics and Illumina.** The intersection includes both partially and fully concordant SNPs.

13484

2299326 (2298094 + 1232)

***CG***

***Illumina***

1817

***CG+IL***

**Figure 11: Intersection of filtered SNPs between Complete Genomics and Illumina.** The intersection includes both partially and fully concordant SNPs.

# Indel detection and intersection

|  | Number of indels*** | Found in dbSNP, 1kGP or ESP 6500 |
| --- | --- | --- |
| **CG:** |  |  |
| Total | 513698 | 372963 (72.6 %) |
| Specific | 101035 | 31427 (31.1 %) |
| **Illumina:** |  |  |
| Total | 646199 | 459587 (71.1 %) |
| Specific | 233536 | 118051 (50.5 %) |
| **Overall:** |  |  |
| Total | 747234* | 491014 (65.7 %) |
| Fully concordant | 351229 | 295119 (84.0 %) |
| Partially concordant | 61434 | 46417 (75.6 %) |

**Table 10: Number of indel calls distributed by concordance**

*Overall total = 351229+61434+233536+101035 = 747234

101035

412663

(351229+61434)

***CG***

***Illumina***

233536

***CG+IL***

**Figure 12: Intersection of small indels between Complete Genomics and Illumina.** The intersection includes both partially and fully concordant indels.

4064

136236

(135305+931)

***CG***

***Illumina***

12541

***CG+IL***

**Figure 13: Intersection of filtered small indels between Complete Genomics and Illumina.** The intersection includes both partially and fully concordant indels.

# Platform-specific calls and non-variant calls in the other platform

**Figure 14: Distribution of CG-specific SNPs over IL calls**

**Figure 15: Distribution of IL-specific SNVs over CG calls.** Most of the IL-specific SNPs are no-calls by CG. Around two-thirds of these CG no-calls are in repeat regions (UCSC hg19 RepeatMasker)

For CG, a position was considered a no-call if both alleles were not called in the variations file. It was considered a reference call when the position was homozygous reference. For Illumina, a position was considered a no-call if the consensus FASTA sequences contained an “N” (no-call) at the position of the SNV. It was considered reference if the position in the consensus sequence was equal to the reference.

**Figure 16: Distribution of CG-specific SNPs over IL calls**

**Figure 17: Distribution of IL-specific SNPs over CG calls.** Most of the IL-specific SNPs are no-calls by CG.

As far as indels are concerned, a position was considered a no-call (or reference) for CG, if the end points of the indels were homozygous no-calls (or homozygous reference) in the variations file. For Illumina, they were considered no-call (or reference), if the end points of the indels were homozygous no-calls (or homozygous reference) in the consensus sequence.

# Mapping of CG substitutions to SNVs and indels

For each block substitution called by CG, we found the overlapping variants from IL and combined them along with information about their zygosity to create all possible haplotypes. If there was a possible haplotype generated by IL that was the same as the block substitution called by CG, the CG block substitution was labelled as concordant to IL.

The remaining CG block substitutions were either in IL no-call regions, overlapped with IL variants with no matching haplotype, or were reference in IL.

**Figure 18: Distribution of CG- block substitutions over IL calls.** Most of the IL-specific SNPs are no-calls by CG.

# Rationale for filtering of variants

For each of the distributions below, an ROC curve will also be generated.

**Figure 19: Distribution of CG read depths of platform-specific SNVs and SNVs shared by both platforms (left). True positive rate and false positive rate for various filter thresholds. (right)**

The distribution of CG read depths of shared SNVs shows a greater proportion of SNVs at higher CG read depths compared to platform-specific SNVs. So for example, choosing a read depth threshold of 20 would remove a larger proportion of platform-specific SNVs than shared SNVs, which we assume is the ground-truth for filtering are assigned a higher confidence.

In a similar manner, we filter SNVs, indels and substitutions using various filter parameters – uncertain calls, read depth coverage (by CG and IL), variant score (by CG and IL), proximity to indels, presence in microsatellites, simple repeats, segmental duplications, self-chained regions and homo-polymer runs.

We used the filter thresholds for CG and IL platforms calculated by Reumers et al. as show below -

| **Parameter** | **Values or value ranges** |
| --- | --- |
| Uncertain calls | Both genotypes (CG and IL) are fully called |
| Read depth coverage (CG) | 20-100 |
| Read depth coverage (IL) | 20-70 |
| Variant score (CG) | > 60 |
| Variant score (IL) | > 100 |
| Distance to indels | > 5 |
| Presence in microsatellites, Simple repeats, Segmental duplications, Self-chained regions | Variant must lie outside these regions (downloaded from UCSC) |

# Structural variations using Complete Genomics

| Structural variation events | Number of events | With baseline frequency < 0.1 |
| --- | --- | --- |
| Complex | 154 | 124 |
| Distal duplication | 87 | 19 |
| Deletion | 1929 | 140 |
| Distal duplication by mobile element | 2 | 1 |
| Inter-chromosomal | 112 | 35 |
| Inversion | 12 | 11 |
| Probable inversion | 84 | 25 |
| Tandem duplication | 170 | 20 |
| Artifact | 1094 | 124 |

Table 11: All structural variation events by type. Baseline frequency corresponds to the frequency of that variation in the Complete Genomics 69 genomes dataset

| Structural variation events | Number of events | With baseline frequency < 0.1 |
| --- | --- | --- |
| Complex | 101 | 13 |
| Distal duplication | 52 | 11 |
| Deletion | 1258 | 67 |
| Distal duplication by mobile element | 52 | 11 |
| Inter-chromosomal | 1 | 1 |
| Inversion | 8 | 7 |
| Probable inversion | 27 | 4 |
| Tandem duplication | 74 | 9 |
| Artifact | 0 | 0 |

Table 12: High confidence structural variation events by type Baseline frequency corresponds to the frequency of that variation in the Complete Genomics 69 genomes dataset

Additionally, Complete Genomics also provides mobile insertion elements (MEIs), which are produced by junctions where one pair was mapped and the other mapped to a ubiquitous sequence.

| **MEI element types** | **Counts** |
| --- | --- |
| Alu | 2057 |
| LINE-1 | 1072 |
| LTR | 26 |
| MER11 | 5 |
| PolyA | 1 |
| SVA | 109 |
| HERV-K | 1 |
| **Total** | **3271** |

Table 13: Number MEI events by element type. LINE-1 stands for Long interspersed elements, LTR for Long terminal repeats, MER11 is a retroviral LTR proliferated by the virus HERV-K11, PolyA is the poly-A tail, SVA refers to SINE/VNTR/Alu, and HERV-K is an endogenous retrovirus.

| Small deletions | 319 |
| --- | --- |
| Small insertions | 356 |
| SNPs | 3802 |
| Block substitutions | 955 |
| **Total** | 5432 |

Table 14: Number of small variants overlapping with MEI insert region

# Structural variations using Illumina

In order to call structural variation for alignments from Illumina, we used 2 algorithms – BreakDancerMax (version 1.1r11) and Pindel – and a pipeline merging the 2 algorithms, called SVMerge (version 1.1). Both structural variation callers (BreakDancer and Pindel) as well as the one used by CG, depend on identifying SVs by detecting anomalies in the separation lengths or orientation of aligned read pairs. Breakdancer adds a confidence score to the structural variations identified in regions with anomalous read pairs, based on a Poisson model using the number of anomalous read pairs, size of the region and coverage of the genome. It identifies indels of sizes 10 base pairs (bp) to 1 mega base pair (Mbp). Pindel detects breakpoints of indels. Using the anchor point of a mapped read on the reference genome, and the direction of the unmapped read, it breaks the unmapped read into 2 (deletion) or 3 (short insertion) fragments and map them separately. It identifies breakpoints of insertions of size 1bp – 20 bp, and deletions of size 1 bp – 10 kbp.

|  | **Breakdancer** | **Pindel** | **Breakdancer+Pindel** |
| --- | --- | --- | --- |
| Number of deletions | 387 | 4006 | 4239 |
| Largest deletion size | 426192 | 8787 | 426192 |
| Upper quartile | 904.5 | 1751 | 1666 |
| Median | 479 | 358 | 371 |
| Lower quartile | 216 | 296 | 282 |
| Smallest deletion size | 91 | 99 | 91 |
| Mean deletion size | 6544.1 | 1457 | 1889 |

**Table 15: Large deletion size statistics for sequences from Illumina**

The deletion events from Complete Genomics and Illumina were combined by first creating a list of potential deletion events taken from both platforms and then, testing if the two platforms contain an overlapping deletion region.

538

3298

***CG***

***Illumina***

2332

***CG+IL***

**Figure 20: Venn diagram showing overlap between large deletions called by Complete Genomics and Illumina.** Deletions are overlapping if there is a >=50 % overlap.

# Copy Number Variations (CNV)

Copy number variations were measured only for data from Complete Genomics from the file cnvDetailsNondiploidBeta-*.tsv.bz2, which shows the relative coverage levels of every 100 kbp along the genome.

From prior CGH array based studies, the MYCN gene is amplified in SHSY5Y genome. (Do et al.) More recently, Yusuf et al., found however that MYCN gene is not amplified in SH-SY5Y, an observation that we confirmed. Our results confirm that there were no CNV segments found in IL in the region of the MYCN gene (chr2: 16080686-16087129).

Using the knowledge from cytogenomic studies (Yusuf et al.) that the dominant ploidy is 2, the relative coverage levels given by Complete Genomics were converted to absolute coverage levels. These are visualized below along with the results from Yusuf et al.


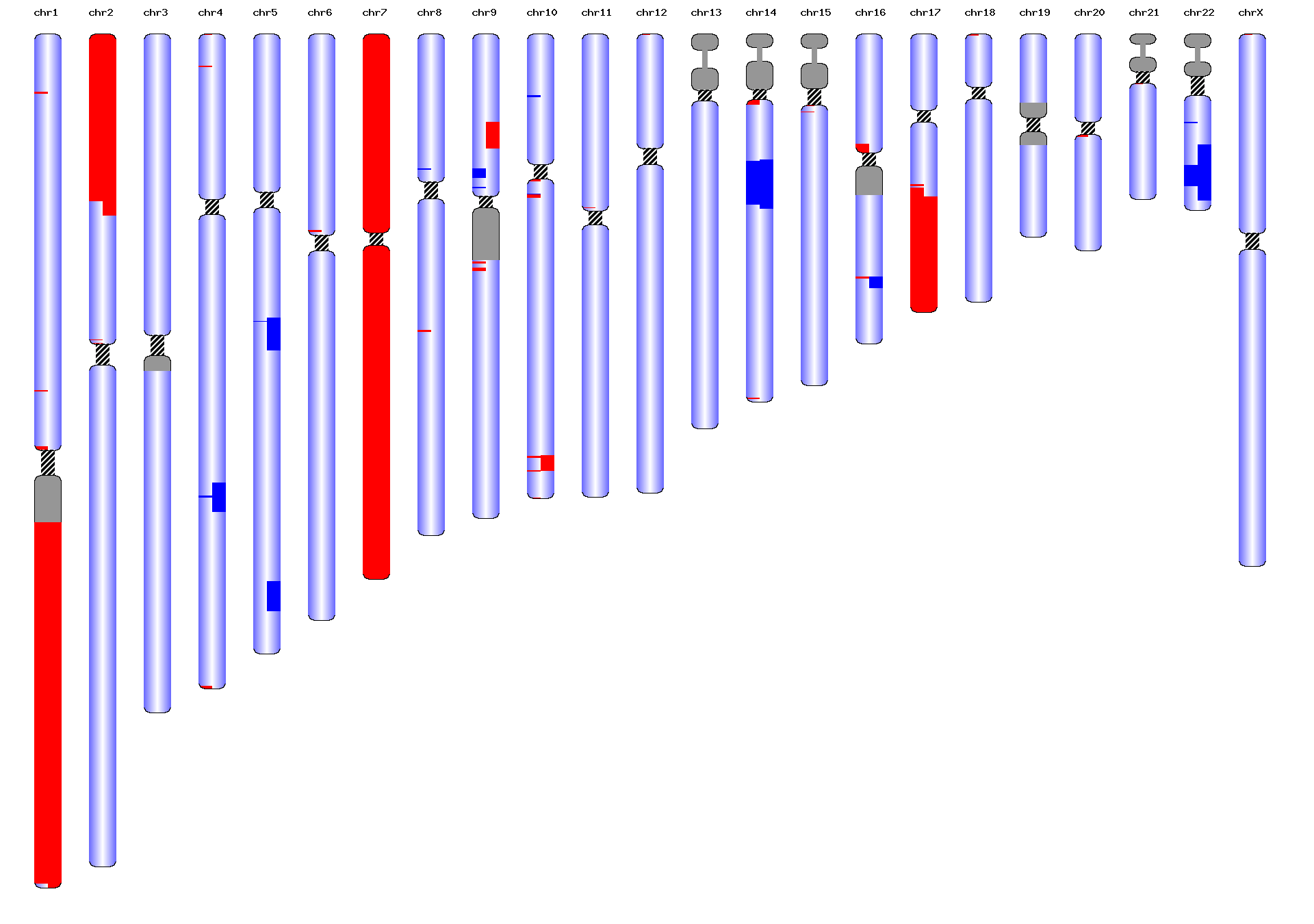


Figure 21: Copy number variation events detected by CG (left half of chromosomes) and microarray analysis by Yusuf et al. (right half). Regions are highlighted for copy number gain (red) and loss (blue). The major events partial trisomy of chromosome 1 and 2, complete trisomy of chromosome 7, gain in 17q and loss in 22q were confirmed.

(Generated using <http://db.systemsbiology.net/gestalt/cgi-pub/genomeMapBlocks.pl>)

# A reference genome

The reference genome of SH-SY5Y was generated by incorporating filtered short homozygous variants (SNPs, indels and subs that were shorter than 200 bp) and can be downloaded from http://systemsbiology.uni.lu/shsy5y/. The zygosity was required to be called homozygous by both platforms. The structural variants were not incorporated as the zygosity was not known reported by Complete Genomics. Similarly, MEIs were also not incorporated.

# RNA-Seq variant calling

Two different variant callers were used to call the variants in RNASeq – SAMTools and GATK. Additionally, the GATK IndelRealigner was also used before the variant calling procedure in order to remove errors due to misalignment around indels.

The concordance statistics of both variant calls are given in Figure 22.

|  | SAMTools | GATK |
| --- | --- | --- |
| Total | 66,730 | 219,138 |
| Specific | 4,318 | 156,726 |
| Concordant | 62,412 | |
| Union | 223,456 | |

**Table 16: Concordance of positions of variant calls from SAMTools and GATK**

4318

62412

***Samtools***

***GATK***

156726

***Samtools + GATK***

62412

***Samtools***

***GATK***

44218

***Samtools + GATK***

Figure 22: Concordance of positions of RNA-Seq variant calls from SAMTools and GATK before filtering (top) and after filtering (bottom). Variants were filtered out if they were caller specific and the read-depth was less than 10.

# SmallRNA variant calling

Two different variant callers were used to call the variants in smallRNA – SAMTools and GATK. Additionally, the GATK IndelRealigner was also used before the variant calling procedure in order to remove errors due to misalignment around indels.

The concordance statistics of both variant calls before and after filtering are given below.

66

541

***Samtools***

***GATK***

3407

***Samtools + GATK***

541

***Samtools***

***GATK***

827

***Samtools + GATK***

Figure 23: Concordance of positions of smallRNA-Seq variant calls from SAMTools and GATK before filtering (top) and after filtering (bottom). Variants were filtered out if they were caller specific and the read-depth was less than 10.

# Concordance between RNA-Seq and DNA-Seq

The discordant variants in the RNA-seq – that is, those not found by DNA sequencing - were checked if they were called by CG and IL.

| Total number of SNVs and small indels in RNA-Seq sequencing* | 106630 |
| --- | --- |
| Total number of SNVs and small indels in RNA-Seq sequencing excluding chrM and chrY | 95173 |
| Number of matching positions to DNA-Seq | 69808 |
| Number of positions with matching alleles to DNA-Seq | 66774 |
| Number of positions with matching alleles found by CG | 61748 |
| Number of positions with matching alleles found by IL | 66229 |
| Number of positions with matching alleles found by CG and IL | 61203 |

**Table 17: Comparison of SNVs found in RNA-seq and DNA sequencing**

# Concordance between DNA-Seq and smallRNA sequencing

The discordant variants in the small RNA – that is, those not found by DNA sequencing - were checked if they were called by CG and IL.

| Total number of SNVs and small indels in smallRNA sequencing | 1368 |
| --- | --- |
| Total number of SNVs and small indels in smallRNA sequencing excluding chrM and chrY | 1349 |
| Number of matching positions to DNA-Seq | 219 |
| Number of positions with matching alleles to DNA-Seq | 130 |
| Number of positions with matching alleles found by CG | 118 |
| Number of positions with matching alleles found by IL | 96 |
| Number of positions with matching alleles found by CG and IL | 84 |
| Number of positions where both DNA-Seq platforms had no-call | 1130 |

**Table 18: Comparison of SNVs found in smallRNA sequencing and DNA sequencing**

The small RNA variants not found in the DNA, do not show a very different distribution of read depths or quality values, compared to those small RNA variants found in the DNA (See Table 11) Notable, none of the 65 indels were found in the DNA sequence.

|  | Found in DNA | Not found in DNA | All |
| --- | --- | --- | --- |
| **Read depth** |  |  |  |
| Minimum | 3 | 2 | 2 |
| 1^st^ quartile | 9 | 8 | 8 |
| Median | 23.5 | 22 | 23 |
| Mean | 226.2 | 184 | 195.3 |
| 3^rd^ quartile | 98.5 | 80 | 82 |
| Maximum | 8010 | 7939 | 8010 |
|  |  |  |  |
| **Quality values** |  |  |  |
| Minimum | 51.30 | 50.00 | 50.00 |
| 1^st^ quartile | 70.75 | 67.00 | 67.50 |
| Median | 101.00 | 91.70 | 93.00 |
| Mean | 118.59 | 110.70 | 112.80 |
| 3^rd^ quartile | 162.25 | 139.00 | 150.00 |
| Maximum | 222.00 | 222.00 | 222.00 |

**Table 19: Five number summary and mean of the read depths and quality values of smallRNA variants**

# Illumina genotyping

SNV calls from Illumina high throughput sequencing were checked using genotyping on the Infinium platform. In a few cases, two different SNV genotypes were called for the same position and the genotype with the higher GC score was chosen for validation, as prescribed by the sequencing provider.

|  | Raw | Filtered |
| --- | --- | --- |
| Total number of het. SNVs and small indels in DNA genotyping excluding chrM and chrY | 248538 | 187928 |
| Number of matching positions to DNA-Seq | 247715 | 187928 |
| Number of positions with matching alleles to DNA-Seq | 246048 | 186993 |
| Percentage of positions with matching alleles to DNA-Seq (%) | 99.0 | 99.5 |
| Number of positions with matching alleles found by CG | 243546 | 186938 |
| Number of positions with matching alleles found by IL | 245547 | 186675 |
| Number of positions with matching alleles found by CG and IL | 243045 | 186620 |

*The number of variants is equal to the number of positions here, as reported by vcf-stats by VCFTools. So multiple variants at the same loci are counted as one.

**Table 20: Comparison of SNVs found in DNA genotyping and DNA sequencing by CG and IL**

# Protein abundance

In addition to the spectra generated by the peptides found in the reference genome, the proteomic database also included the variants found from DNA sequencing. Specifically, the coding sequences of Ensembl genes were modified using all the homozygous exonic variants (unfiltered). 334065 MS/MS spectra were generated by the peptides out of which 165494 were identified, which represented 1410 proteins.

See Additional File 6 for the abundances of proteins detected.

| **Total number of proteins detected** | **1410** |
| --- | --- |
| Number of proteins detected using homozygous variants | 22 |
| Number of proteins detected using all variants | 45 |
| Number of variants validated by proteins detected | 109 |
| Number of SNVs validated by proteins detected | 104 |
| Number of indels validated by proteins detected | 4 |
| Number of block substitutions validated by proteins detected | 1 |

Table 21: Number of proteins detected and validation of genomic variants

# Validation of SNVs and short indels using proteomics

We extended the reference database of peptides by inserting homozygous short variants (SNVs and indels) into exon sequences of proteins. The additional peptides added to the database allowed the detection of 46 additional transcripts. When heterozygous short variants were also included, 35 more transcripts were detected.

Consequently, the extra transcripts detected validated 109 short variants – 104 SNVs and 4 indels and 1 block substitution.

# Genomic mutations and gene expression of SH-SY5Y across 247 conditions

|  | Genes never expressed in the cell line | Genes always expressed in the cell line |
| --- | --- | --- |
| **Total number of genes** | **9589** | **1858** |
| Number of corresponding with NCBI Refseq transcript IDs | 8619 | 2994* |
| Frequency of mutation in these genes (per 100 kbp region) | 165.363 | 131.306 |
| Average number of mutations per gene (normalized by length of gene) | 163.972 | 128.772 |
| Average copy number | 2.09 | 2.18 |

* The number of transcripts is greater than the number of genes (2994 > 1858) as the same gene can have multiple transcripts. ** The total number of genes probed was 26850.

Table 22: Number of mutations and copy number of genes that are always present or always absent in SH-SY5Y transcriptome across 267 samples. The threshold used to determine whether genes were always expressed in the GEO dataset was that the expression values of the corresponding transcripts were always greater than the median for all the transcripts in the microarray. Similarly, the threshold for genes never expressed in the GEO dataset was that the expression values were always less than the median for all the transcripts in each microarray experiment.

# Genetic copy number and gene expression across different conditions

Out of the 5776 genes with high copy number, the expressions of 3042 genes were probed in the GSE9169 dataset from the GEO database. Remaining 2734 genes did not map to an Entrez gene that was probed. For both higher and lower copy genes, we took the average across 86 conditions and then the average across all genes. The average here is not normalised by length of gene and each gene is weighted the same.

| **Group** | **Number of genes** | **Mean expression across conditions averaged across all genes** |
| --- | --- | --- |
| Copy > 2 | 3042 | 6.80 (std. deviation = 7.23) |
| Copy <= 2 | 19388 | 6.28 (std. deviation = 6.72) |

Table 23: Mean expression values for genes with a high copy number

We performed Welch’s t-test, which assumes unequal variance and unequal sample size, to test if the mean expression value of genes with copy number > 2 and genes with copy number <= 2 are the same (that is, the null hypothesis). The null hypothesis was rejected with a p-value of 2.2×10^-16^. Therefore, the mean expression values of genes with copy > 2 are significantly different from the remaining genes.

# Comparison of genetic copy number and RNA-Seq expression

| Copy number of genes | Number of genes |
| --- | --- |
| Not covered | 3532 |
| 1 | 175 |
| 2 | 18921 |
| 3 | 2908 |
| 4 | 21 |
| 5 | 36 |
| **Total** | **25593** |

Table 24: Distribution of genes by copy number.


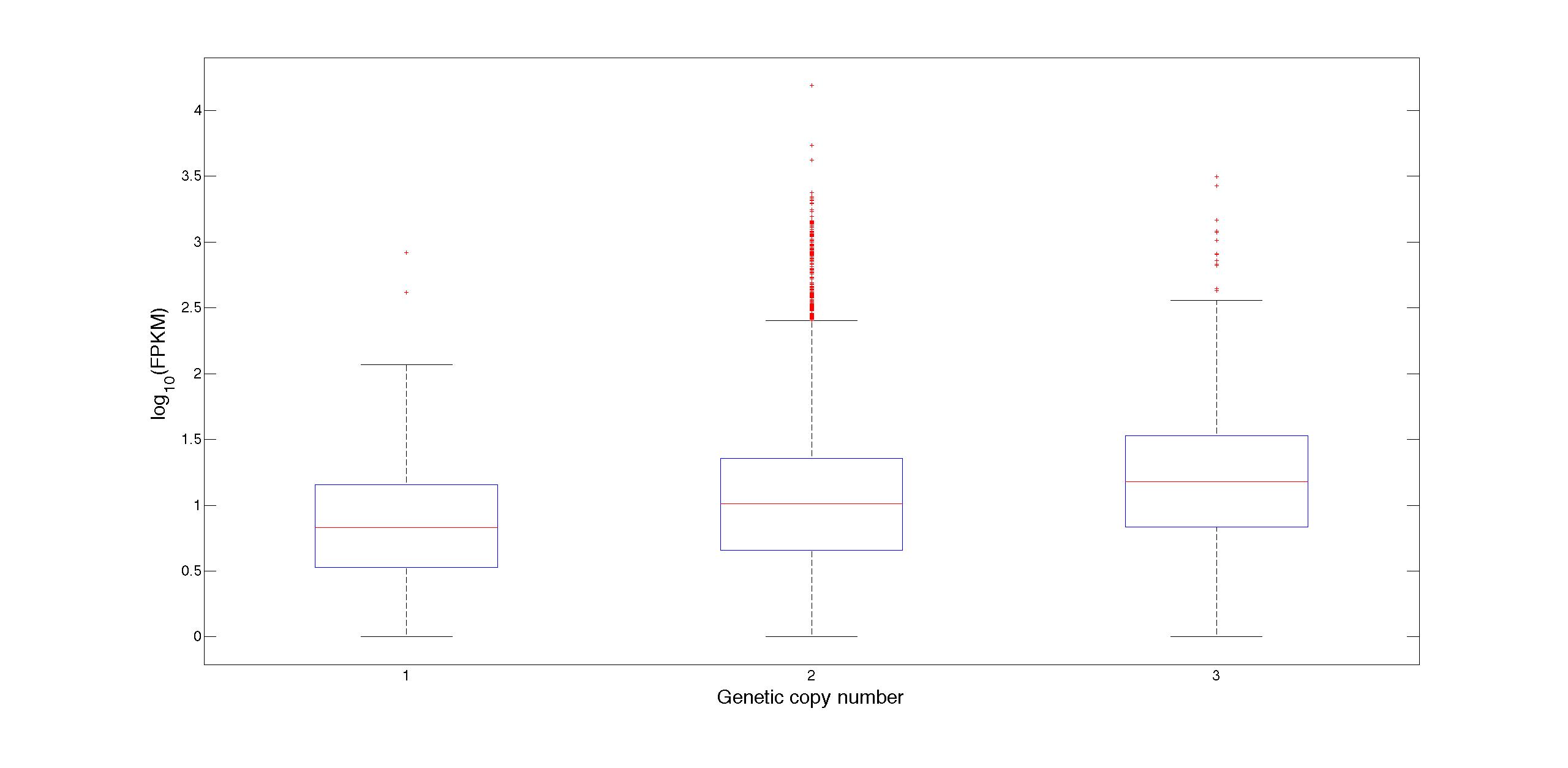


Figure 24: Transcript abundance (for genes with FPKM >= 1) is positive correlated with genetic copy number. There are very few genes with copy number greater than 3. (Table 24) Therefore, these were discarded when looking at the relationship between copy number and RNA expression.

# Comparison of RNA-seq expression and protein abundance of a gene

**1307 genes**

**103**

**genes**

**14773**

**genes**

Figure 25: Venn diagram of the number of expressed genes detected at the mRNA (red) and the protein level (blue)

| Number of genes whose expression values were measured | 56040 |
| --- | --- |
| Number of genes detected at the protein level | 1425 |
| Number of genes expressed in the mRNA (FPKM > 0) | 16498 |
| Number of genes detected at the protein level (iBAQ > 0) | 1410 |
| Genes expressed both in the mRNA and the protein level | 1307 |

Table 25: Number of genes expressed at the mRNA and the protein level

There is a weak correlation between the gene expression and protein abundance of a gene (correlation coefficient = 0.2784). (**Figure 26**). The list of genes that were expressed in the cell line were derived by obtaining a set union of genes found to be expressed using both Ensembl and NCBI Refseq annotation during RNA-seq read-mapping. Both the gene annotations were used because the use of only Ensembl annotation contributed to only 756 genes expressed in both mRNA and the proteins, whereas including the Refseq annotations resulted in 93 % of the proteins expressed having the corresponding mRNA expressed.

**Figure 26: FPKM vs. iBAQ intensities as a proxy for gene expression vs. protein abundance shows a weak positive correlation (Spearman’s correlation coefficient = 0.2784)**

# Suitability of the SH-SY5Y cell line for perturbation experiments in the context of modules in the Parkinson’s’ Disease map

The modules in Table 2 of the main manuscript and the genes corresponding to them were extracted from the Parkinson’s Disease (PD) map (Fujita et al.). We assessed the impact of the genes damage in SH-SY5Y in the context of the above biological modules in PD using a network analysis approach (see Methods section). The genes damaged in the cell line had a greater impact on pathways that had a high BC-score. Therefore, the mutated genes affected glycolysis, calcium signalling and mitochondria more than ROS metabolism and the Ubiquitin Protease System.

Genes were labelled as ‘damaged’ if they contained an exonic SNV, short indel or block substitution, were located in regions with copy number variation or structural rearrangements. Genes affected with either of synonymous, non-synonymous, frameshift or non-frameshift mutations were taken as ‘damaged’. Note that genes may have multiple working copies of a gene, such as they could be triploid, and be labelled as ‘damaged’ as they could show an unusually high expression than normal cells.

1. http://cgatools.sourceforge.net/ [↑](#endnote-ref-1)
2. http://support.illumina.com/sequencing/sequencing software/casava.ilmn [↑](#endnote-ref-2)
