# Supplementary figures and images for: Systems genomics evaluation of the SH-SY5Y neuroblastoma cell line as a model for Parkinson’s disease
Source: BMC Genomics. 2014 Dec 20;15(1):1154. doi: 10.1186/1471-2164-15-1154 (PMC4367834; doi:10.1186/1471-2164-15-1154)

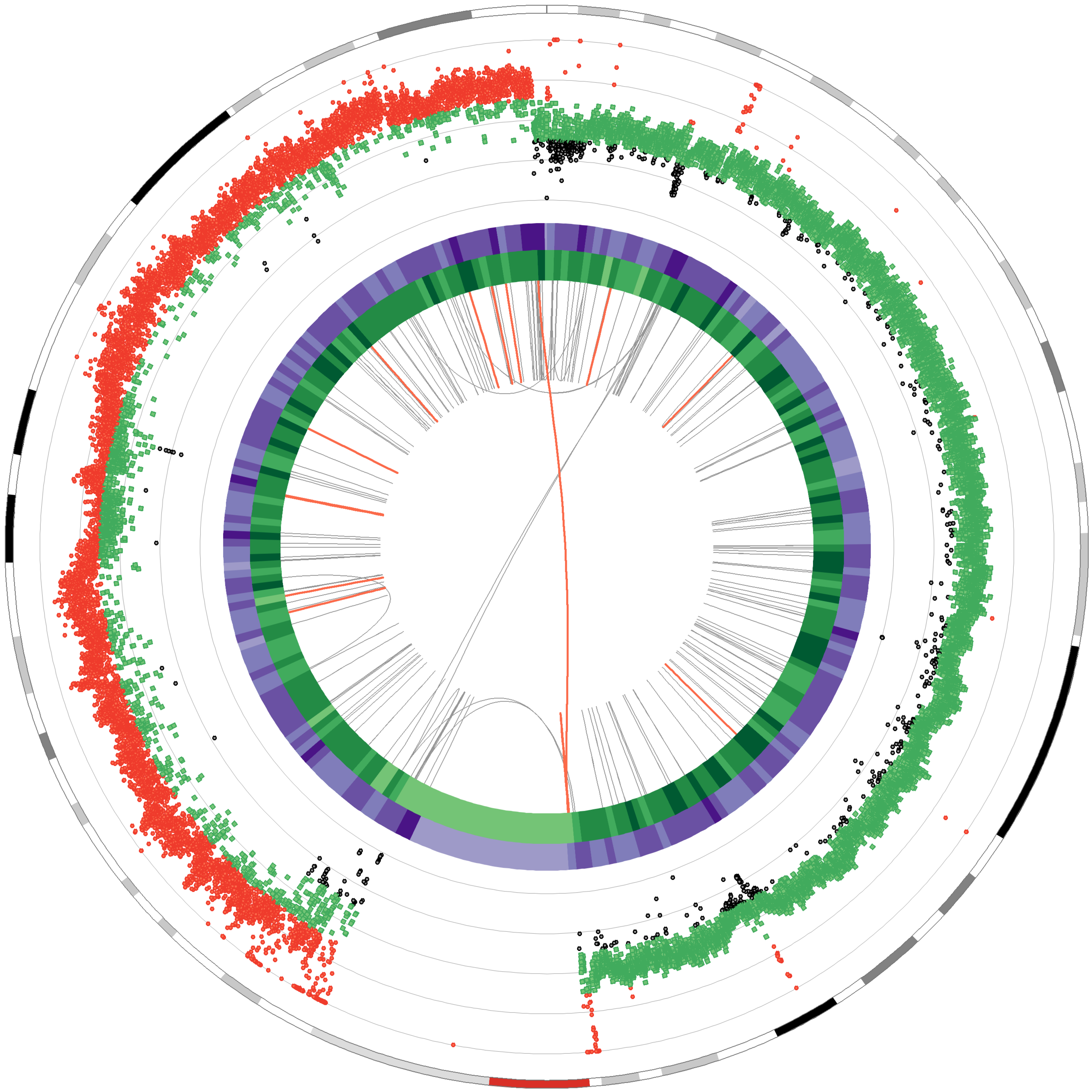

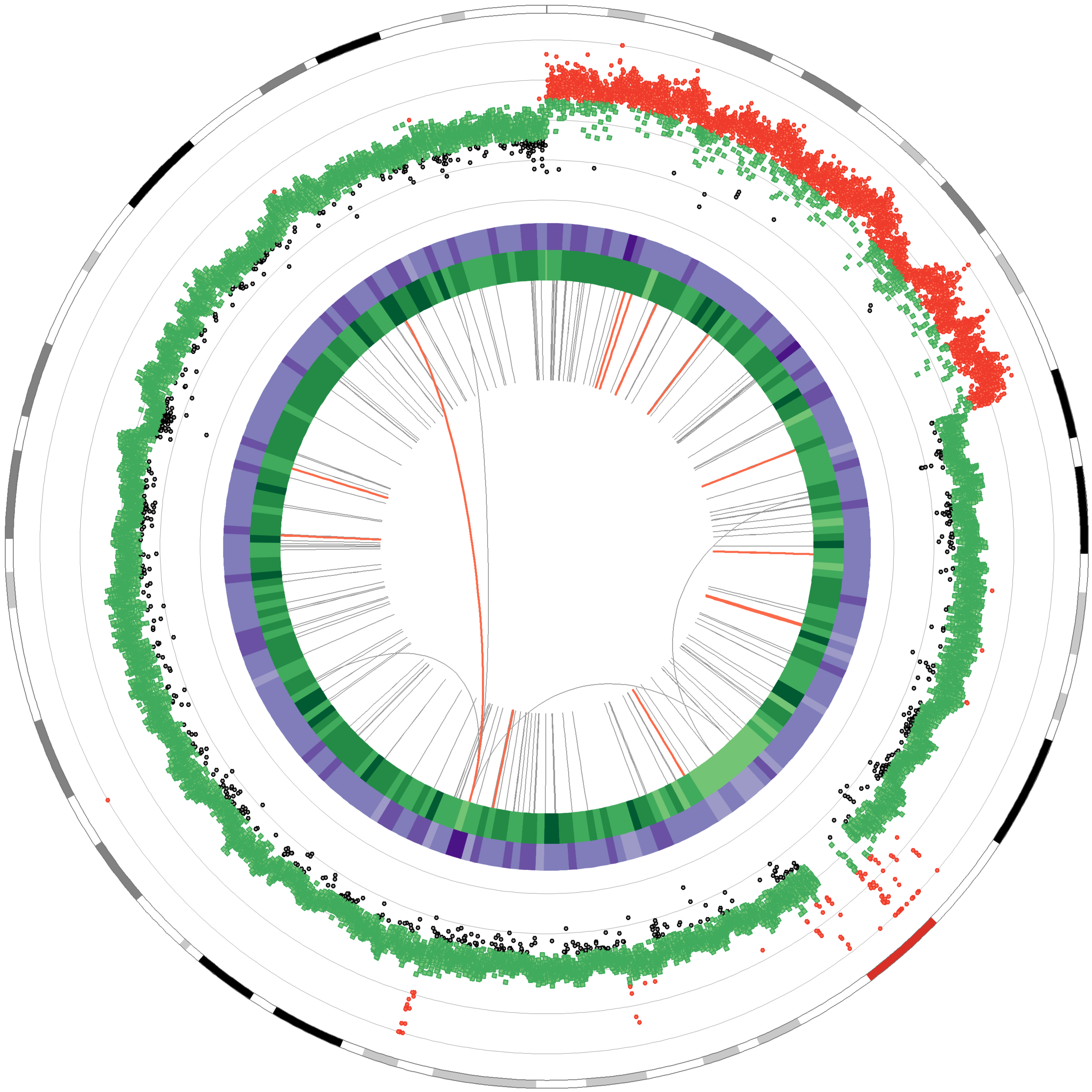

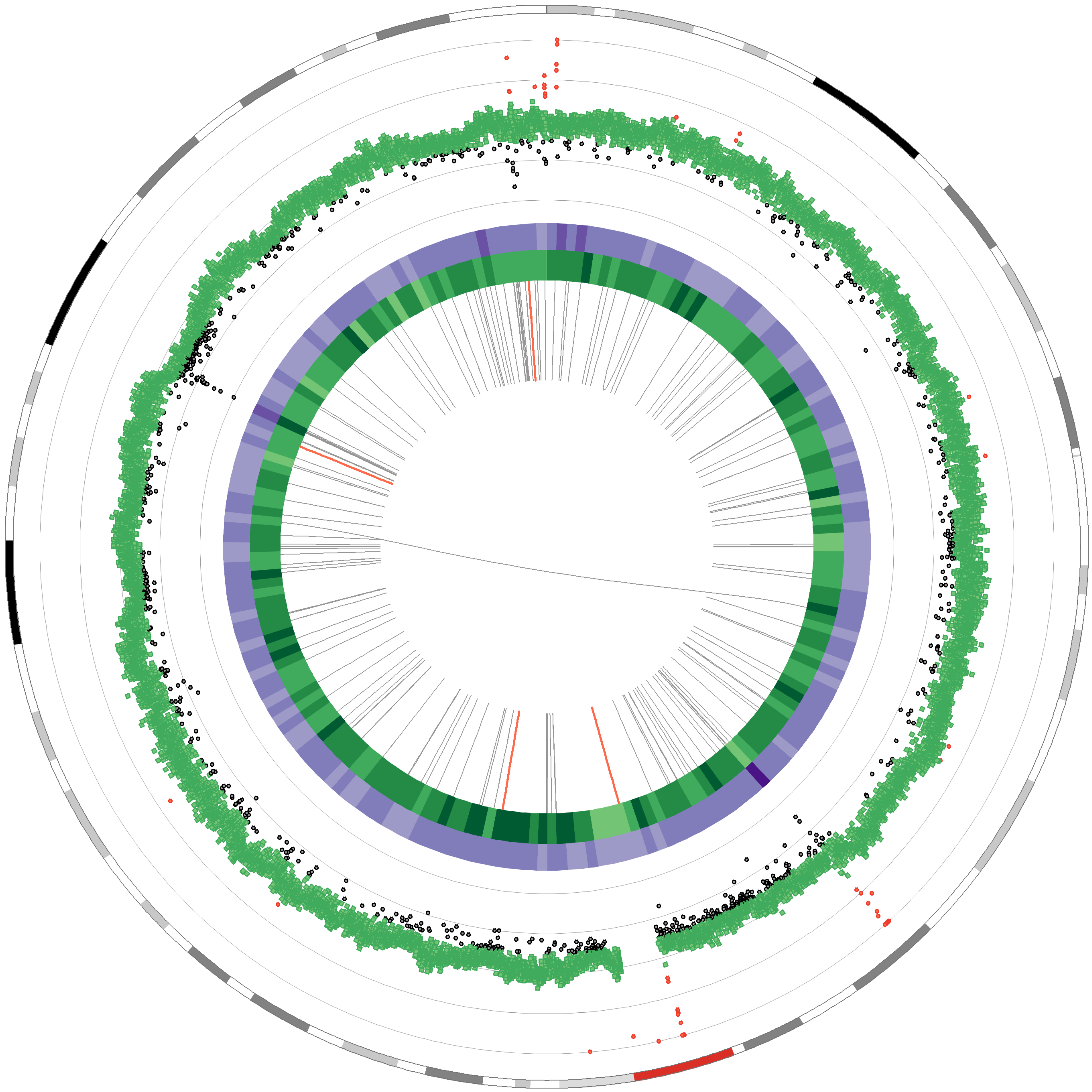

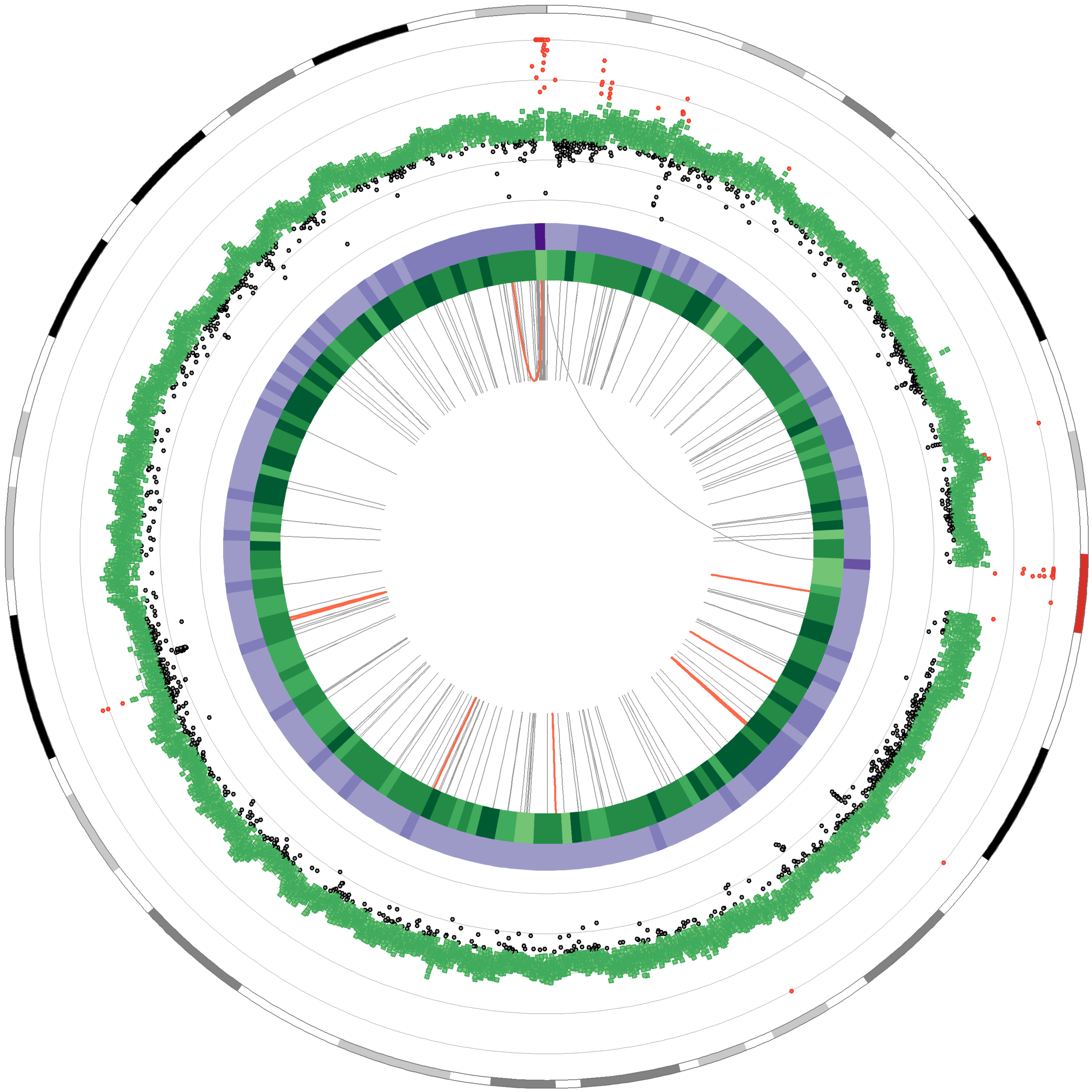

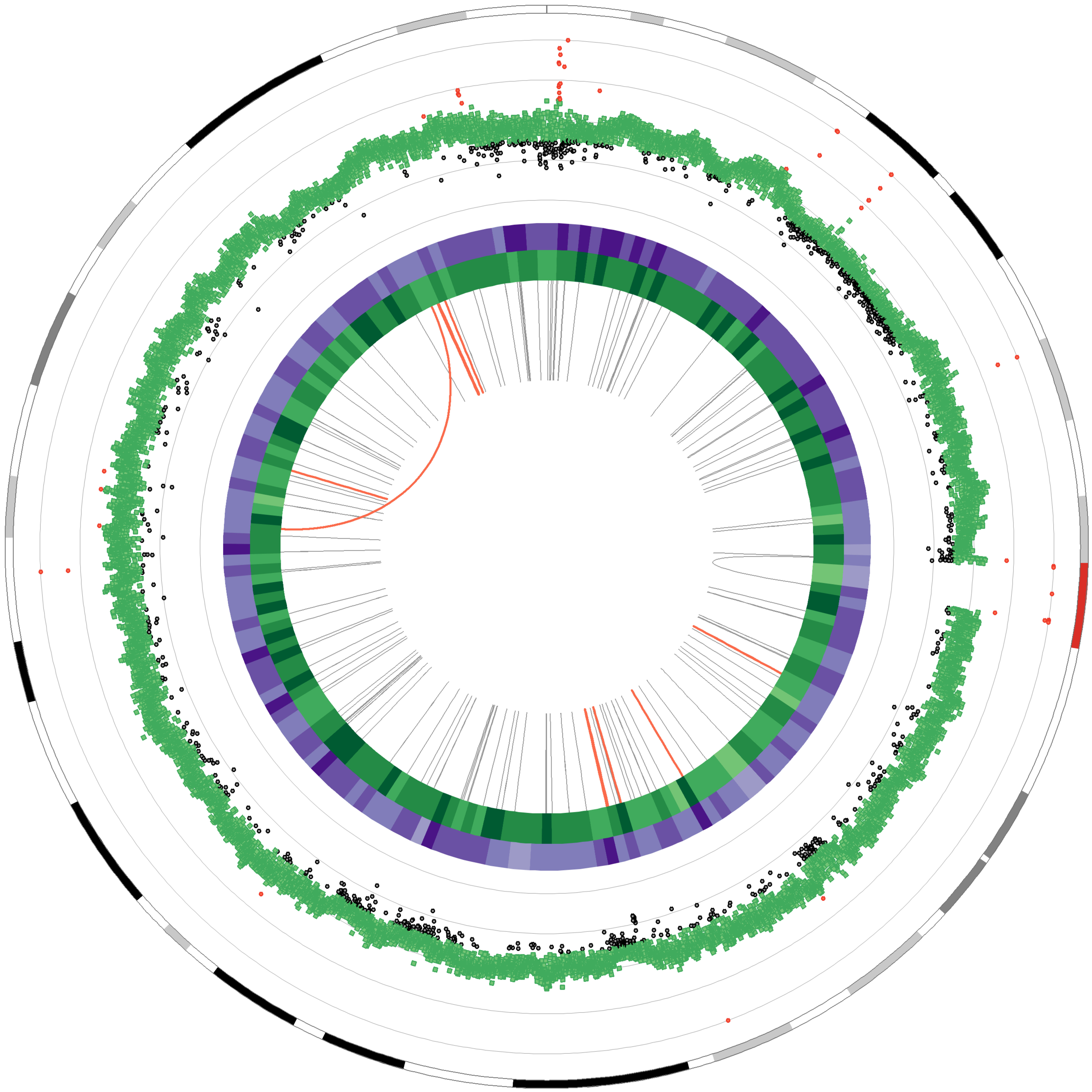

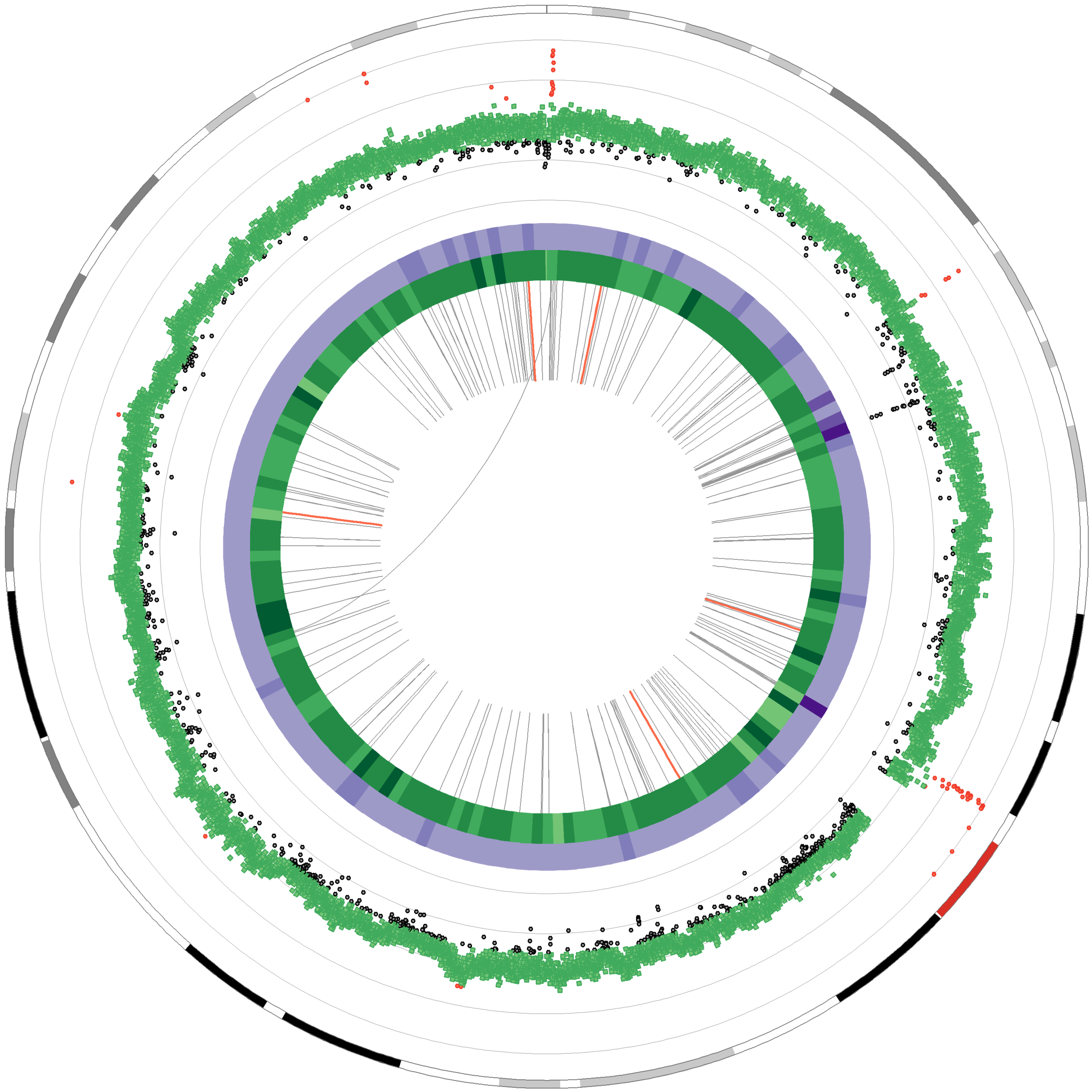

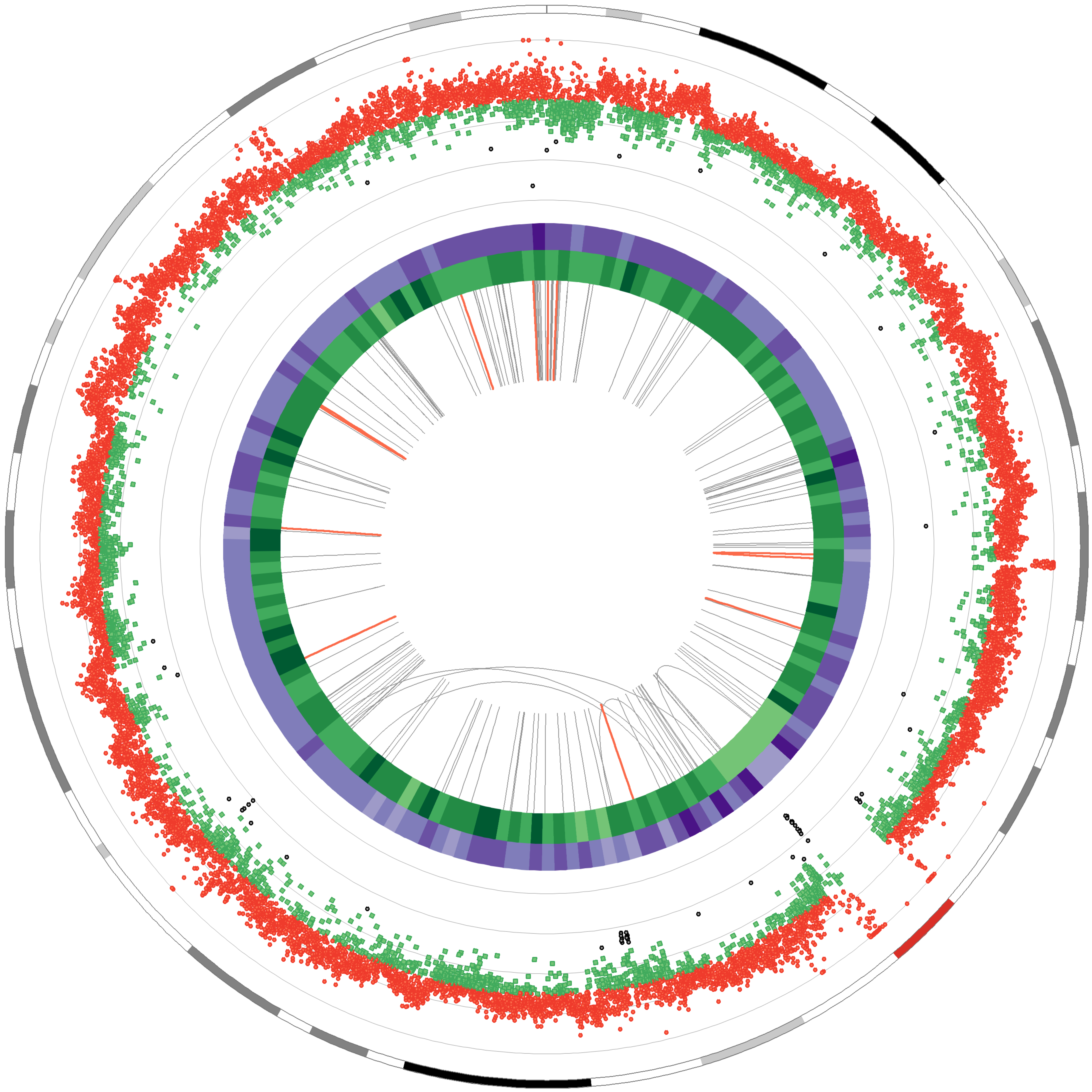

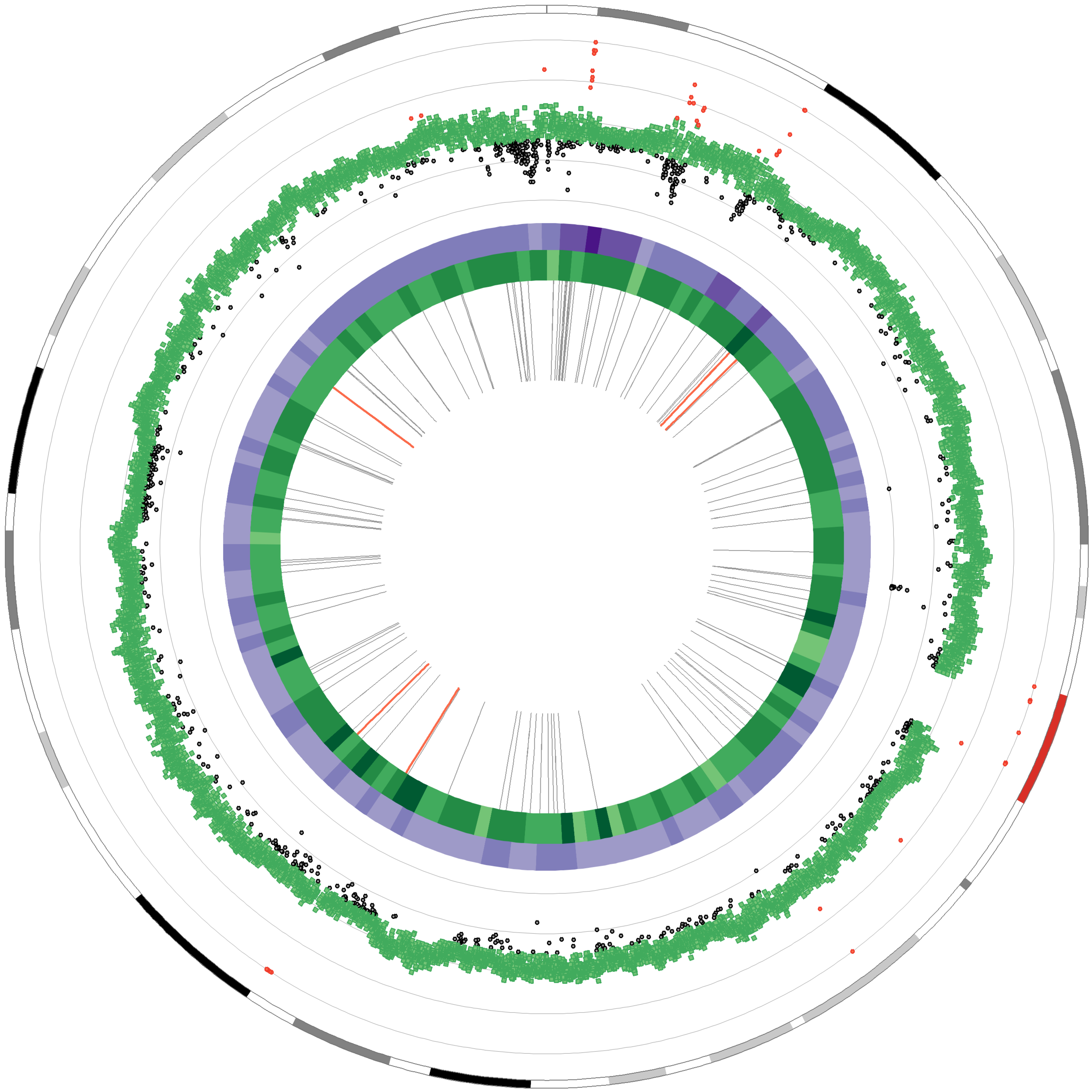

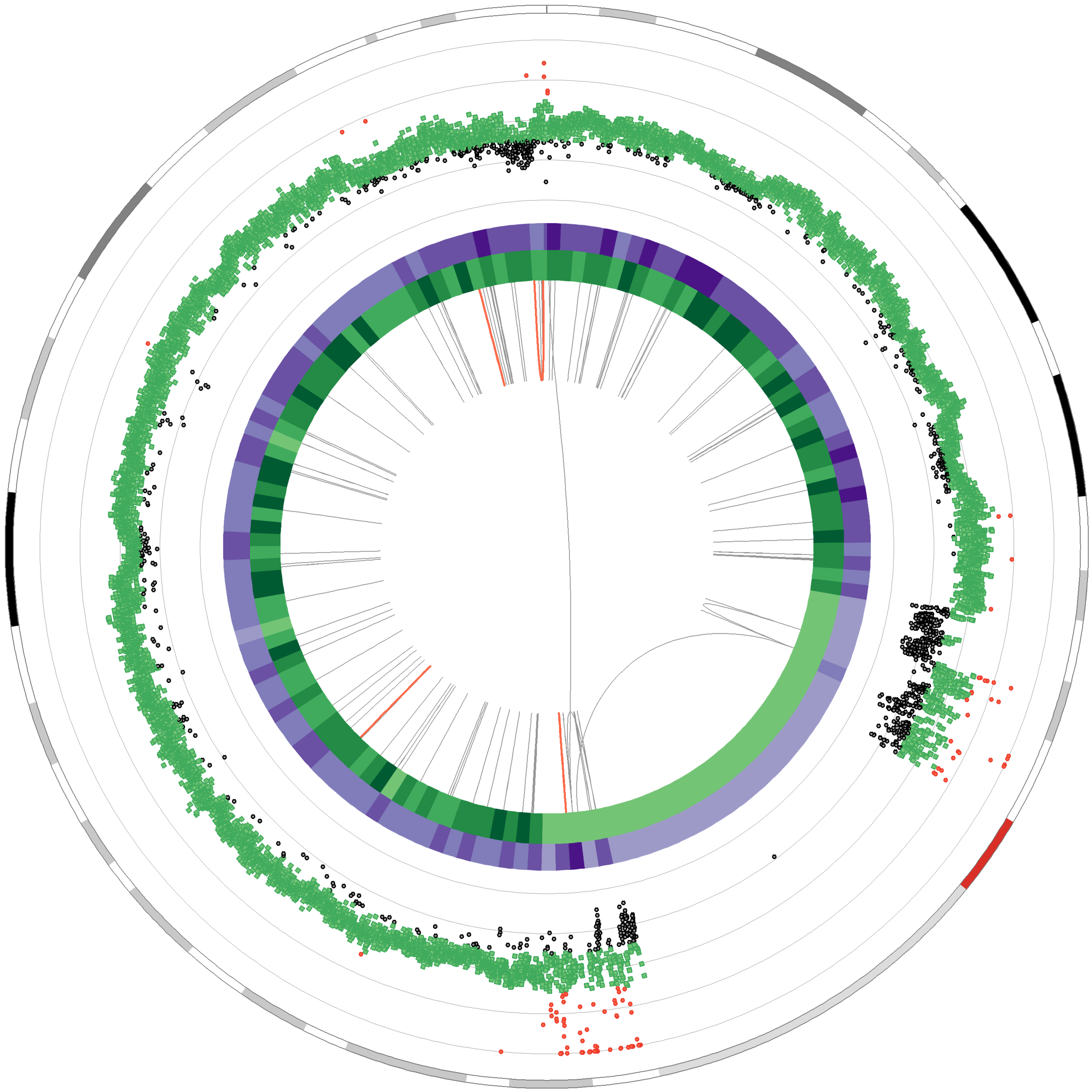

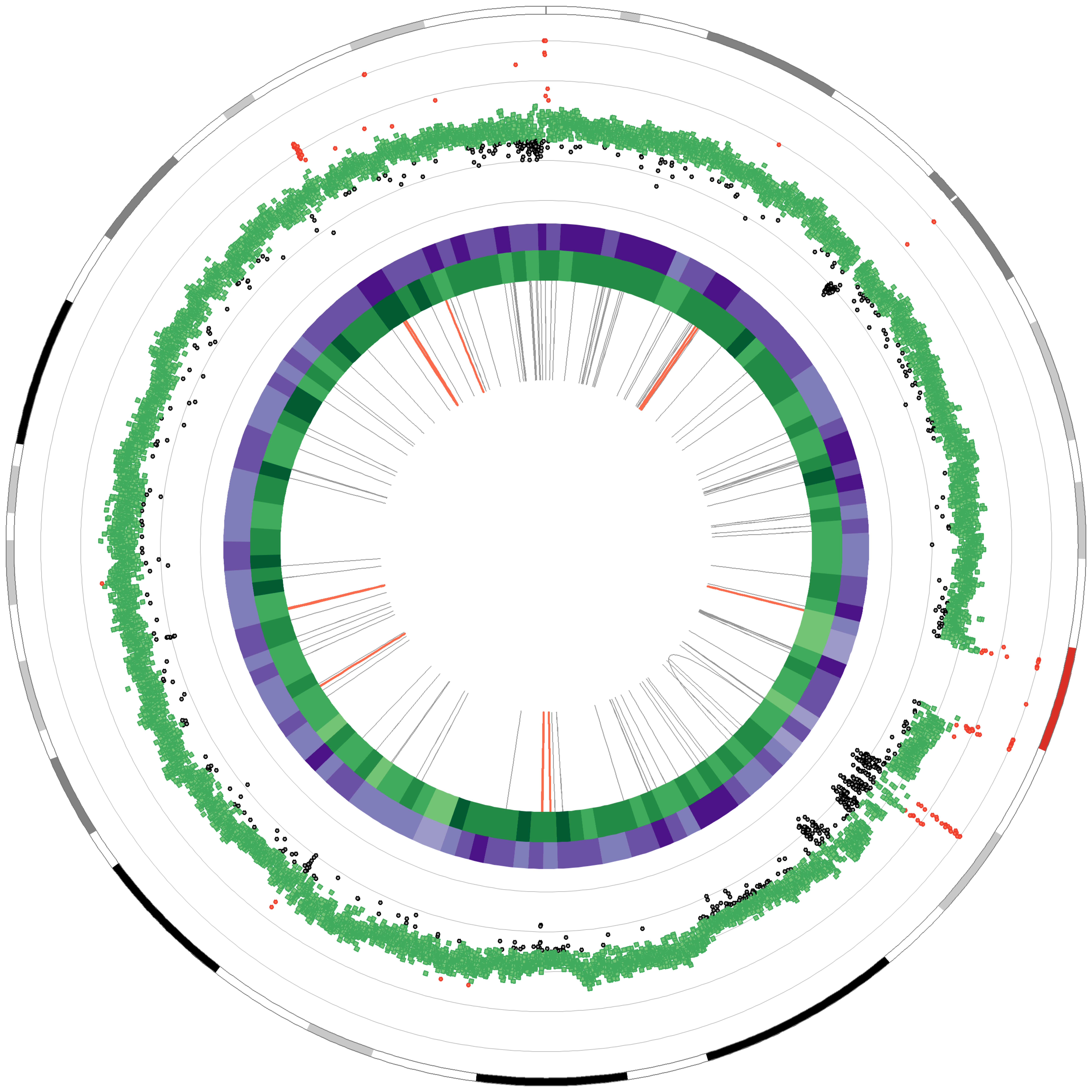

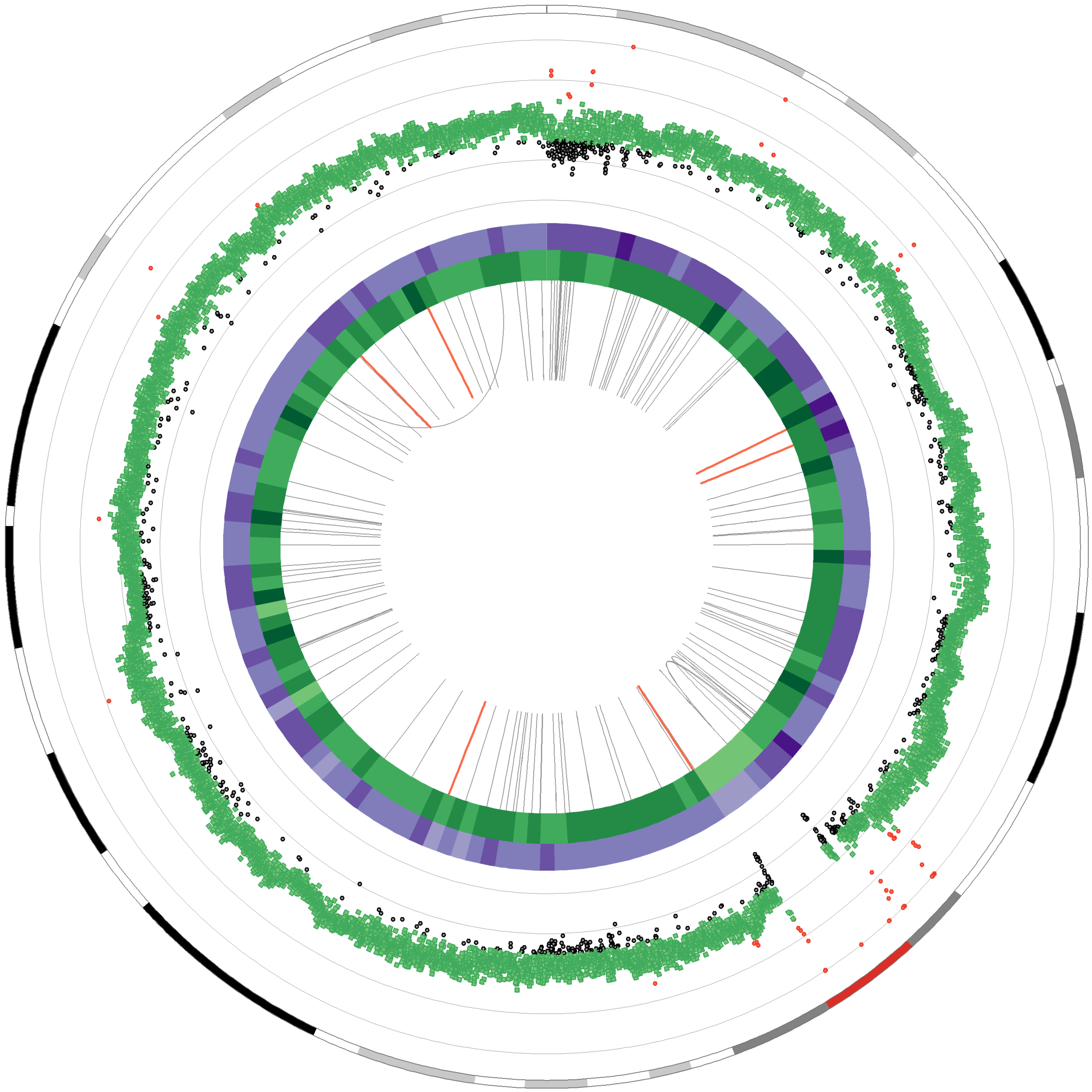

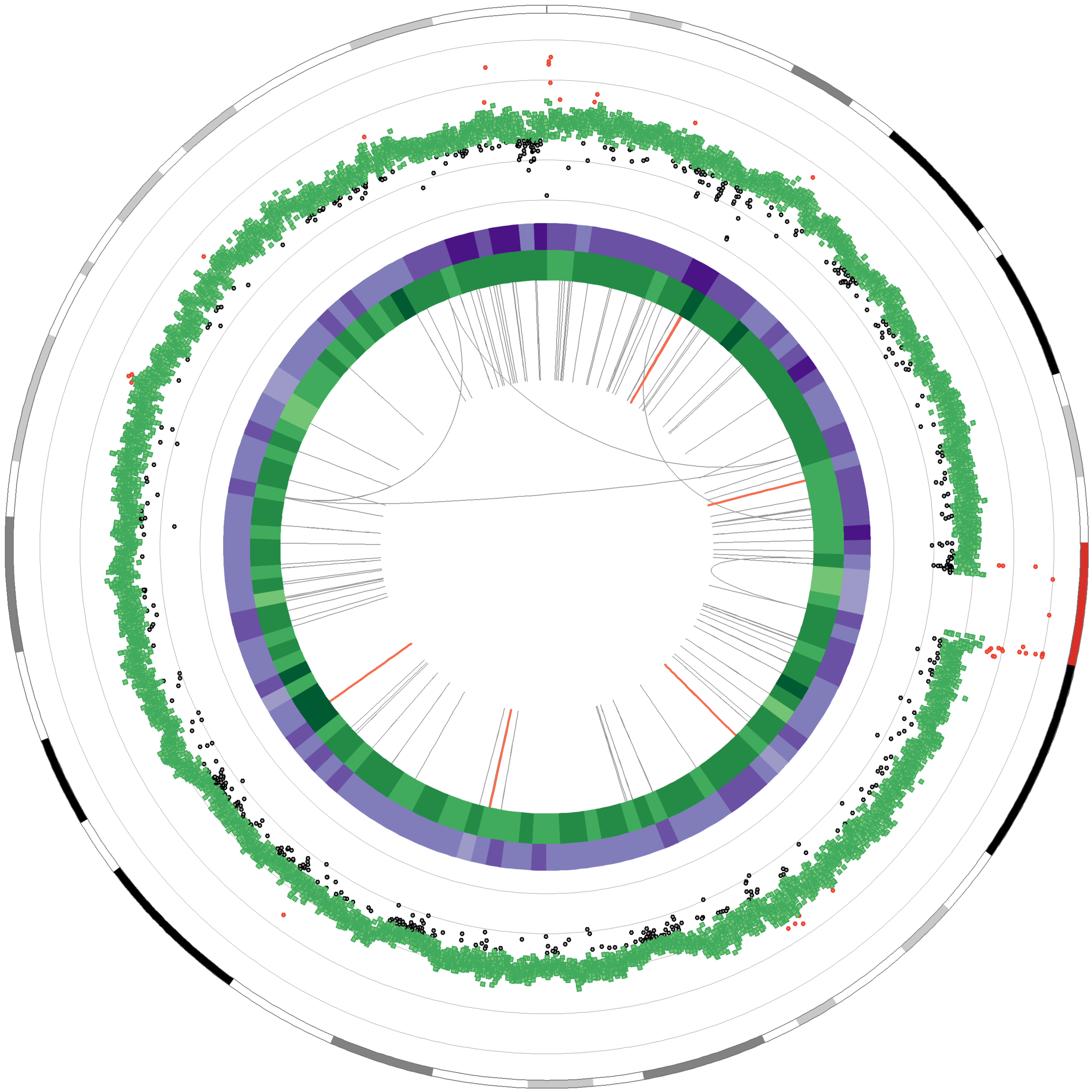

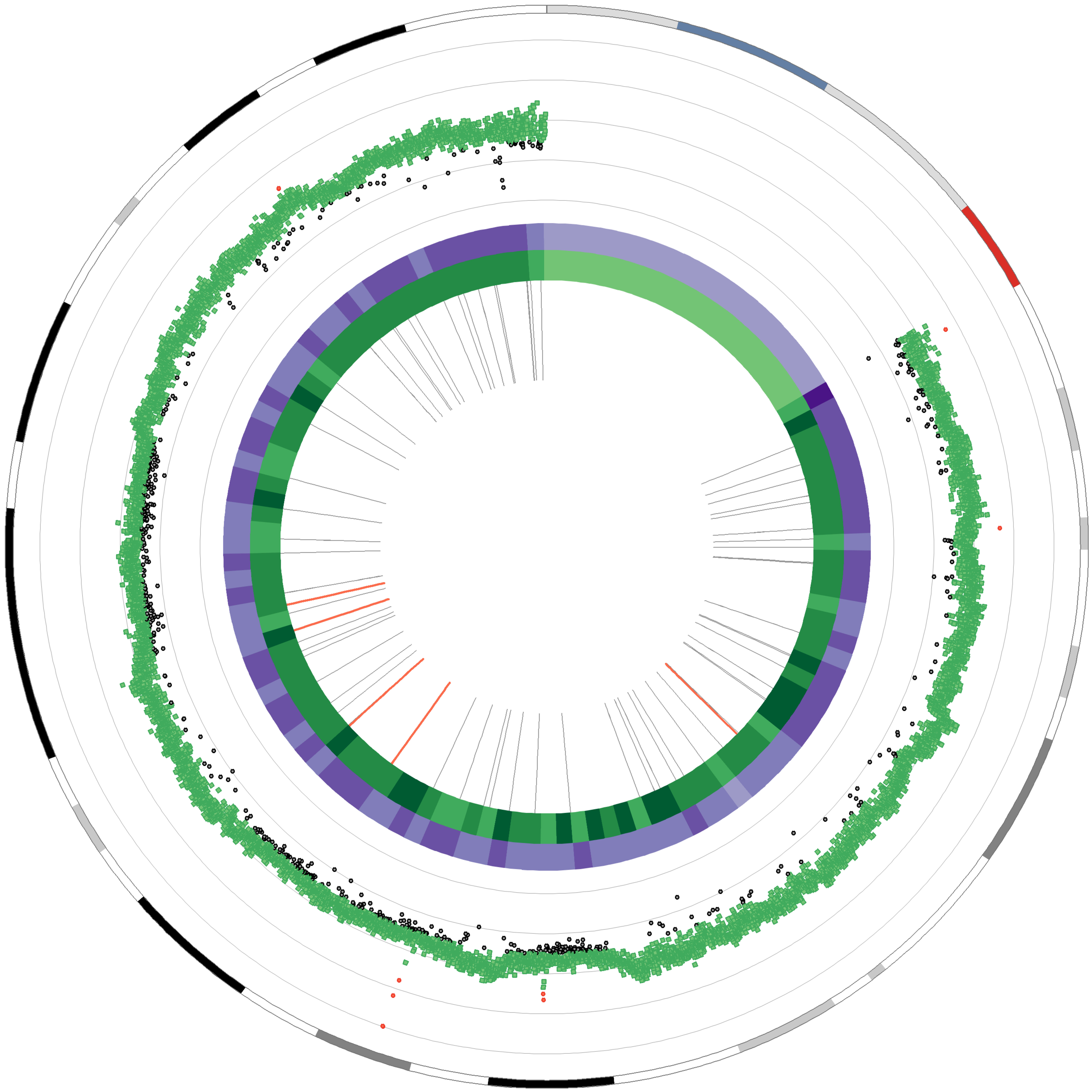

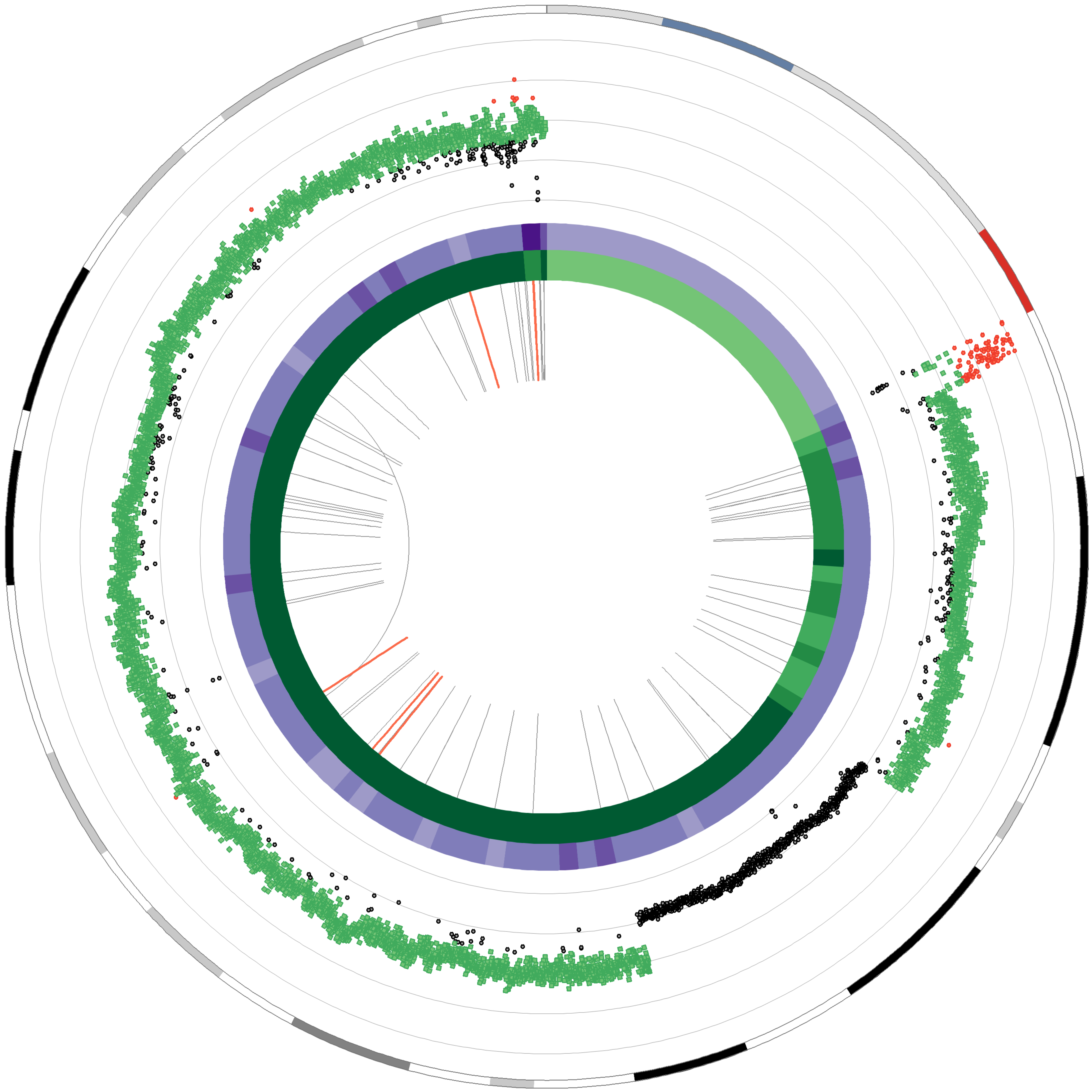

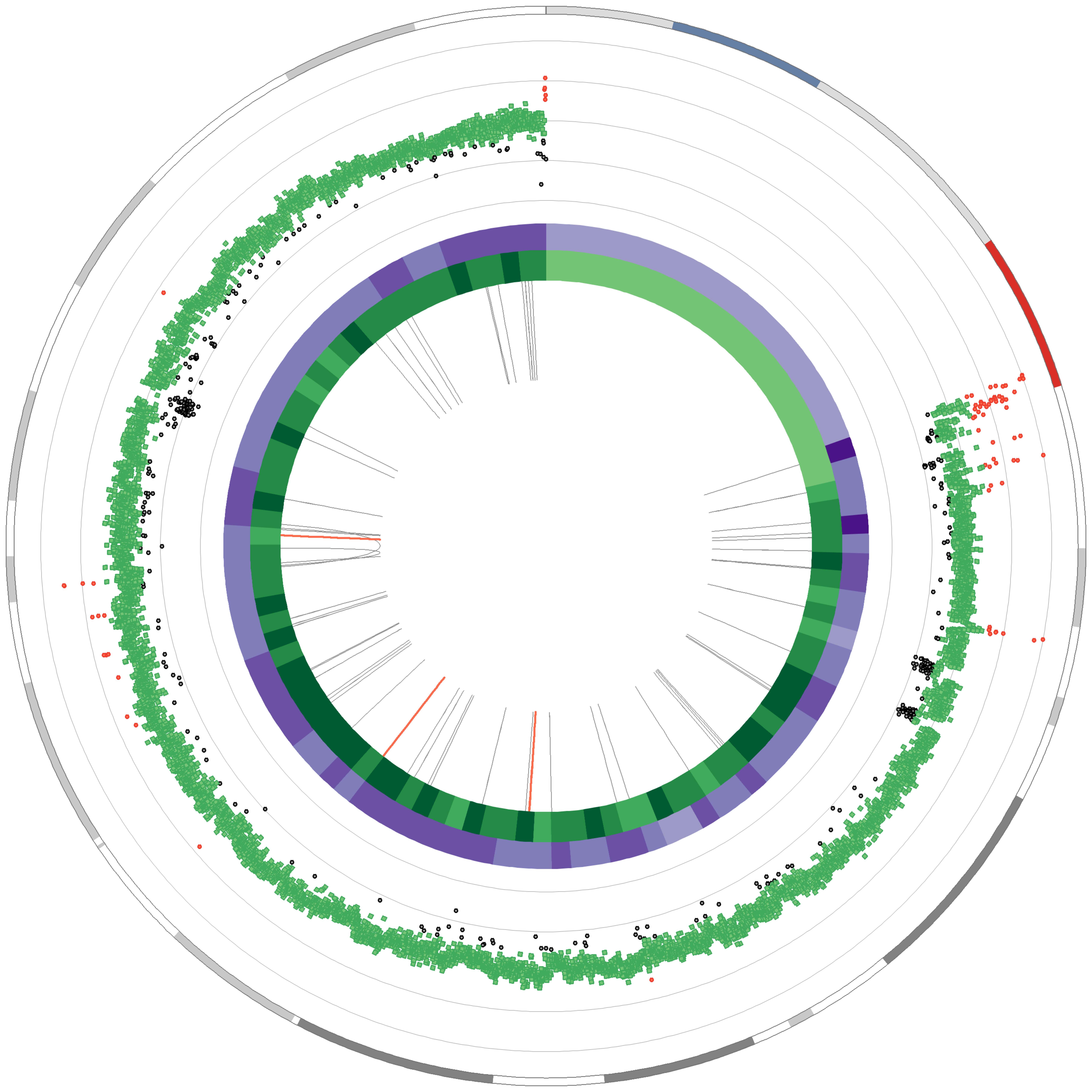

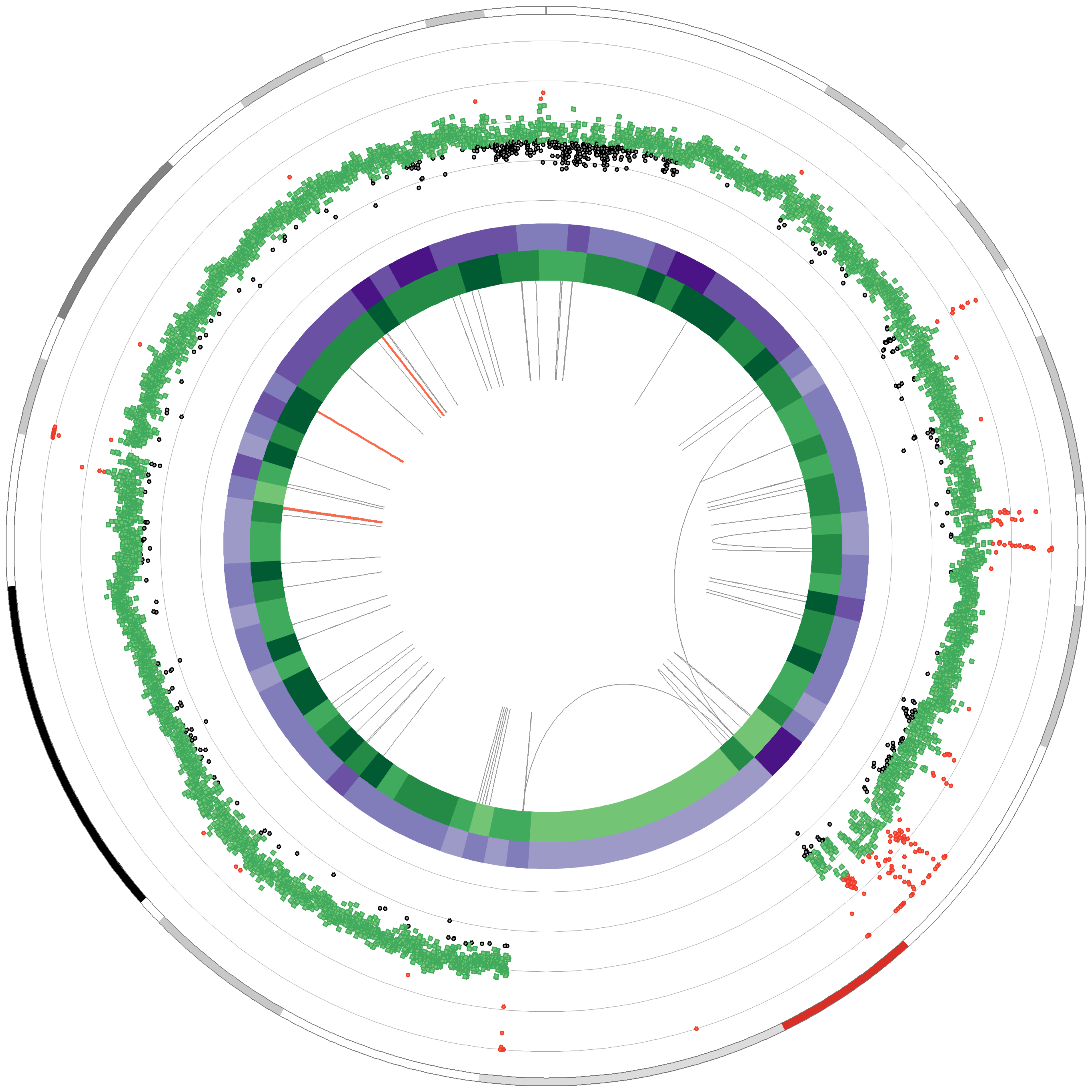

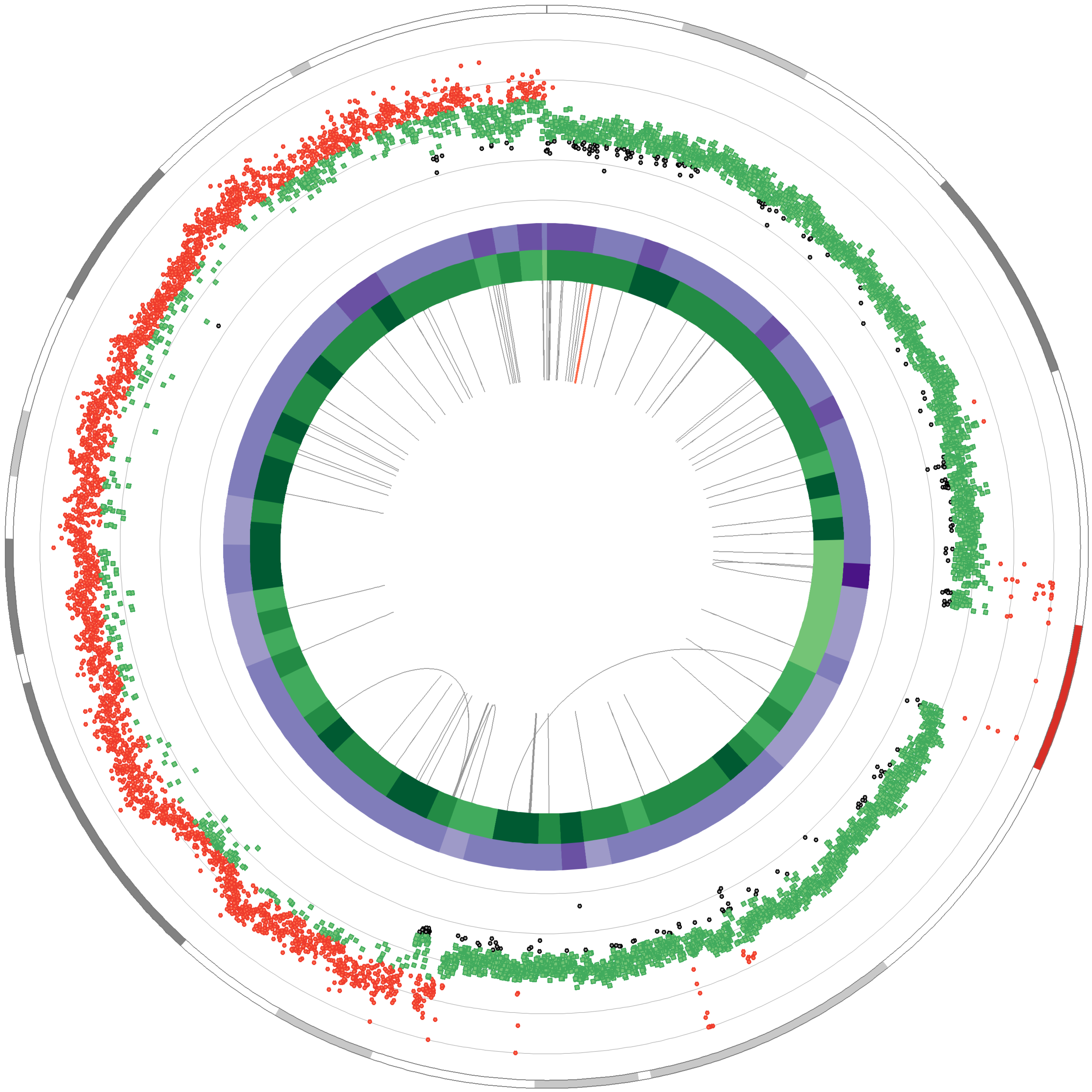

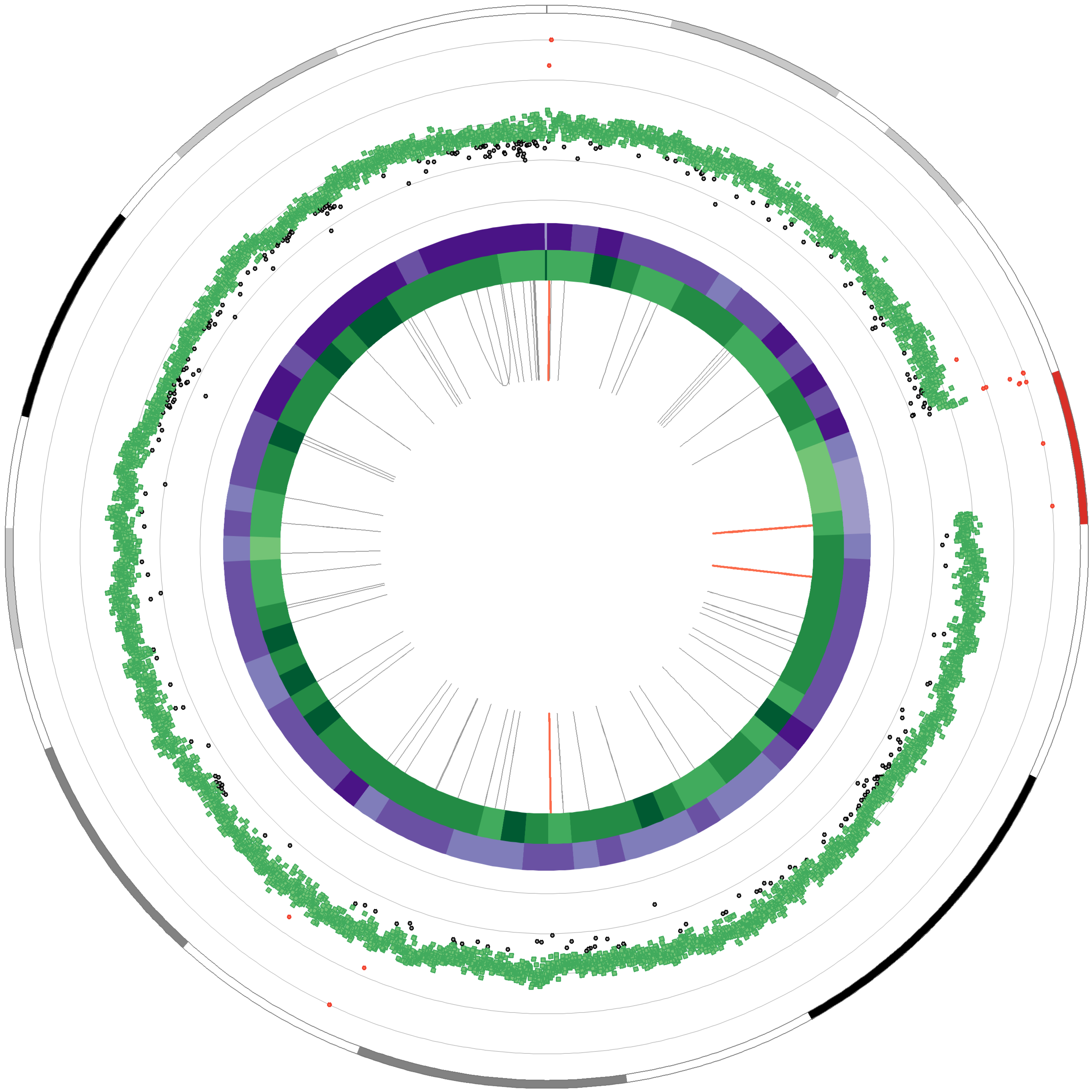

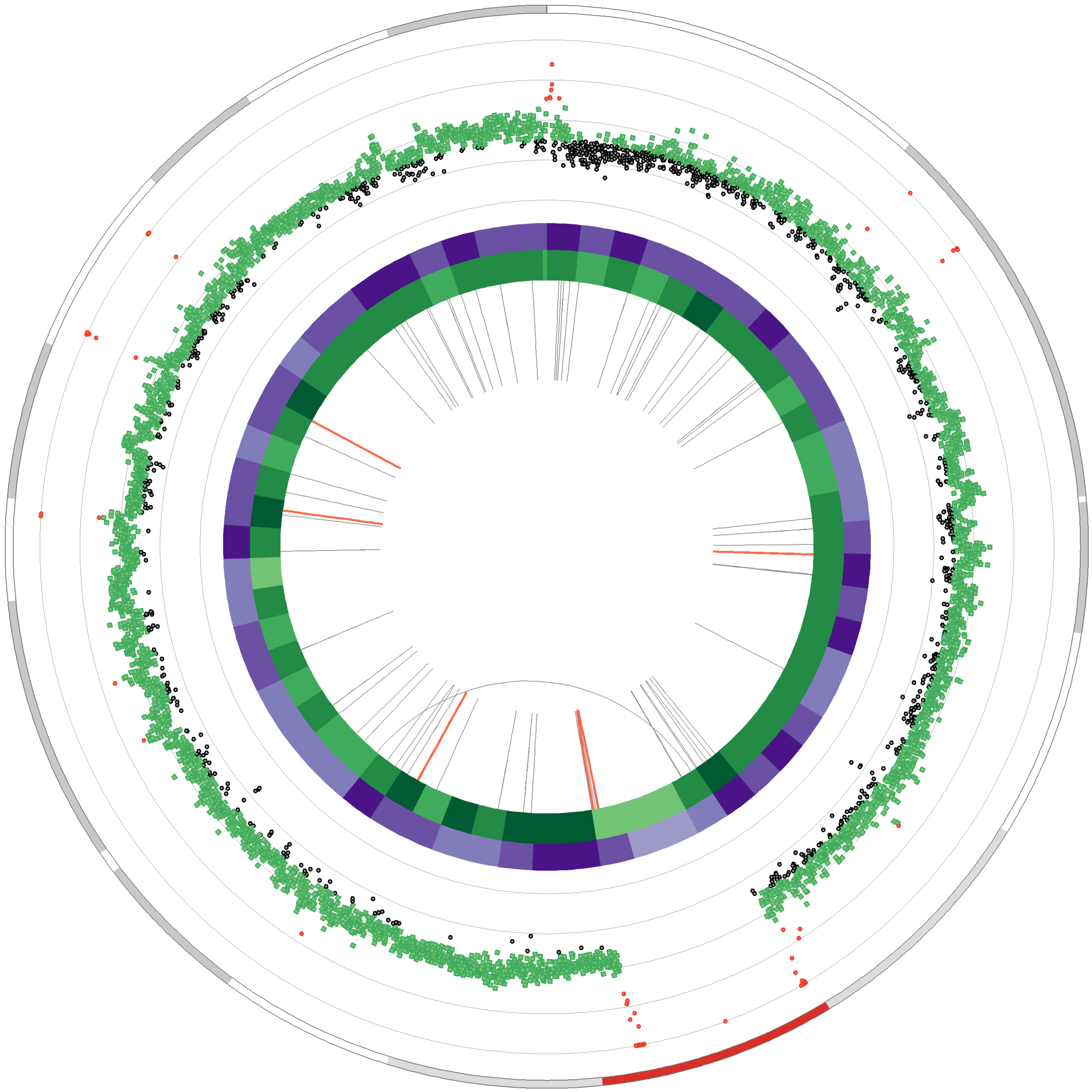

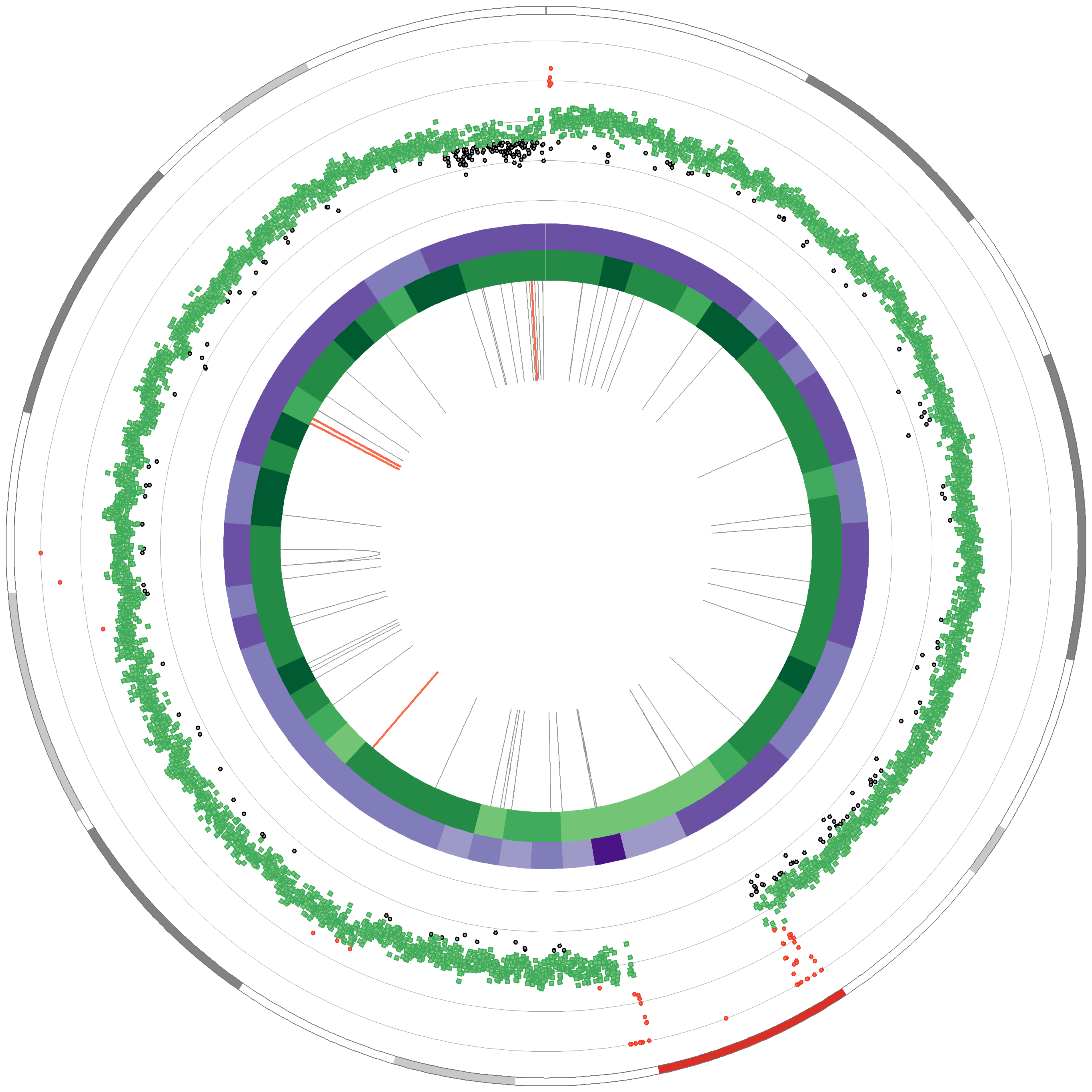

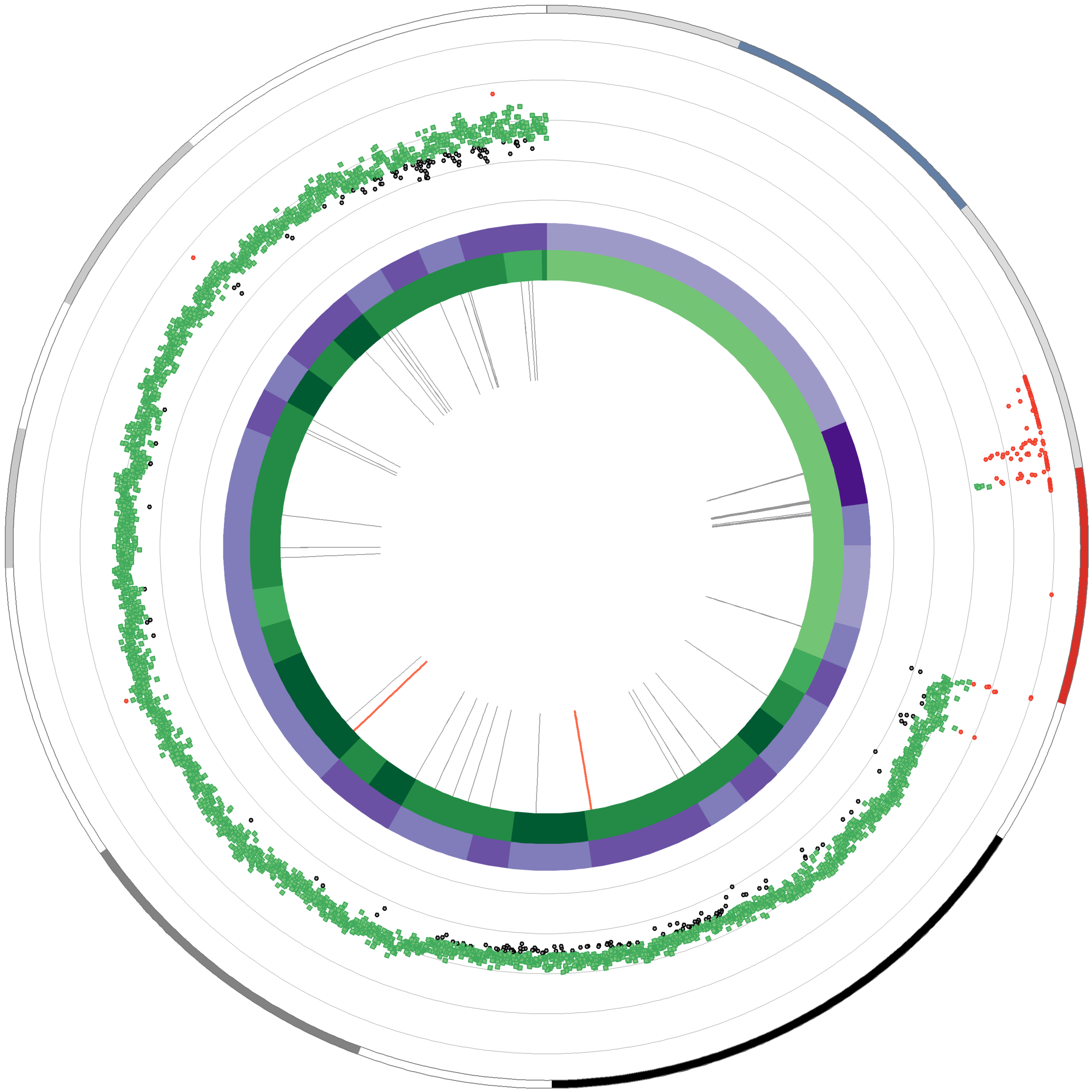

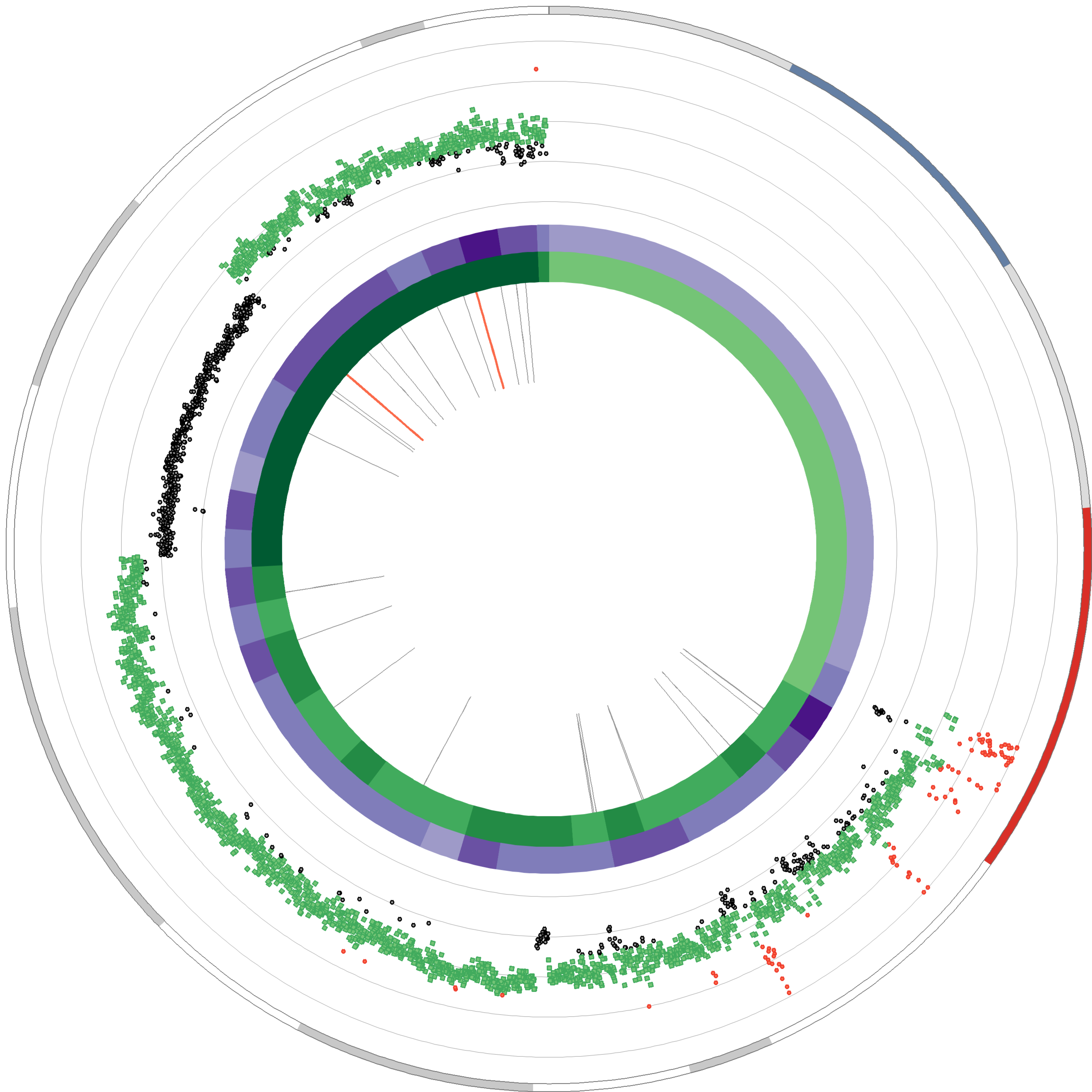

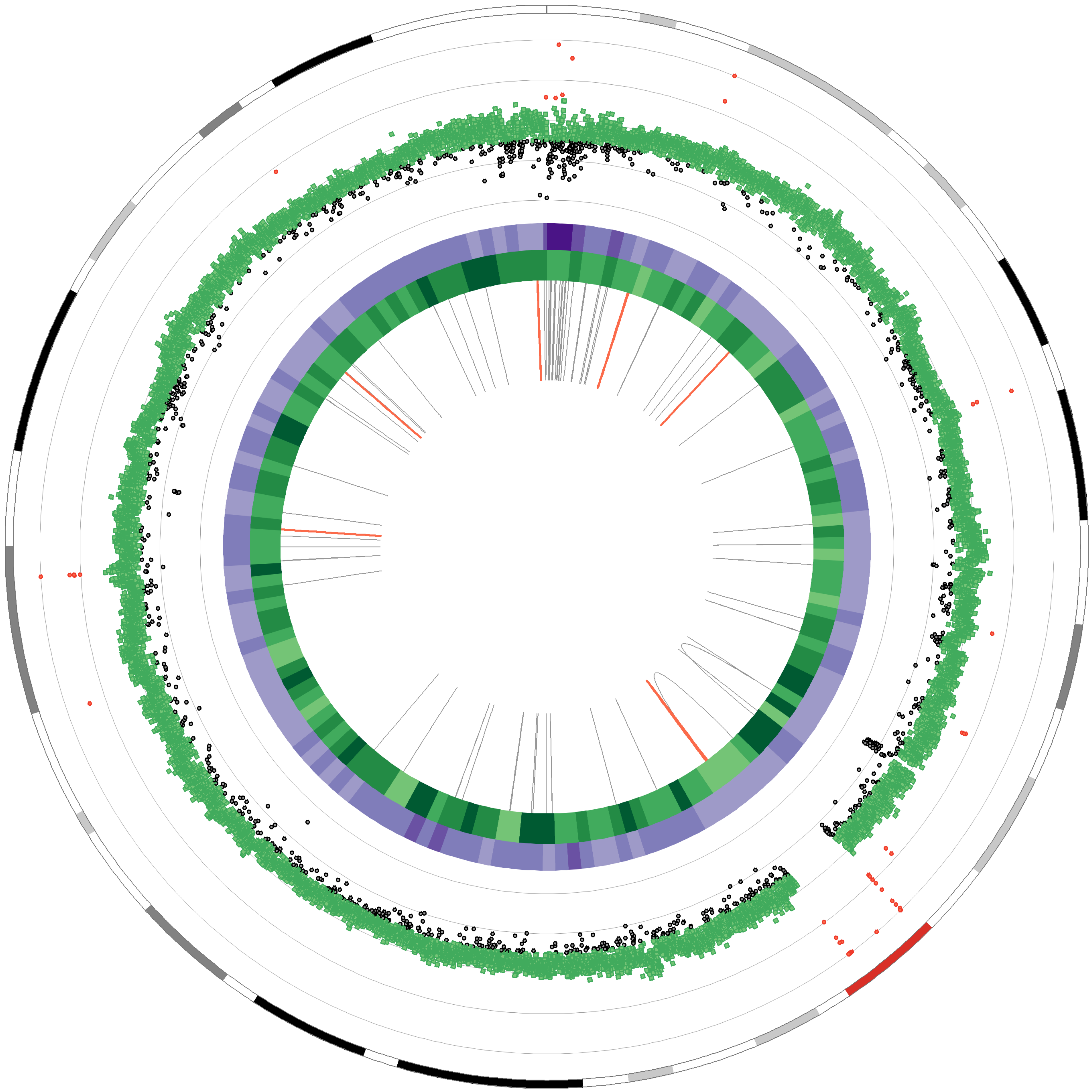

Supplement: Supplementary file 4 — Additional file 4: Circos plots for each chromosome of the SH-SY5Y genome. For each chromosome, tracks represent (from outside to inside) karyotype for each chromosome, copy number variation (red > 2, green = 2, black < 2), density of small variants (bin size = 1 Mb), homozygous small variant percentage (bin size = 1 Mb). Arcs represent chromosomal breakpoints (red = rare breakpoints not found in Complete Genomics Baseline dataset [32]). (PDF 9 MB) [file 12864_2014_6889_MOESM4_ESM.pdf]
