# Supplementary material for: Systems genomics evaluation of the SH-SY5Y neuroblastoma cell line as a model for Parkinson’s disease
Source: BMC Genomics. 2014 Dec 20;15(1):1154. doi: 10.1186/1471-2164-15-1154 (PMC4367834; doi:10.1186/1471-2164-15-1154)
Supplement: Supplementary file 8 — Additional file 8: Materials and methods for proteome analysis. (DOCX 694 KB) [file 12864_2014_6889_MOESM8_ESM.docx]

**Supporting Information - Proteome analysis**

**MATERIALS & METHODS**

***Cell Lysis***

A total of 1.1x10^7^ SH-SY5Y cells were lysed as described previously (Melchior, 2009). Briefly, cells were resuspended in 700 µl hypotonic 5 mM phosphate buffer (0.047% NaH_2_PO_4_; pH 7; 2x concentrated complete protease inhibitor; Roche, Basel, Switzerland), mixed thoroughly and incubated for 1 h at RT with gentle shaking (300 rpm) on an Eppendorf Thermomixer (Eppendorf, Hamburg, Germany), followed by sonication in a water bath on ice for 5 min. Proteins were extracted adding 700 µl trifluoroethanol (TFE) with an additional incubation for 2 h at 60°C and 300 rpm. This was followed by a further sonication in a water bath on ice for 2 min. Cell debris was pelleted by centrifugation at 10,000 g (Heraeus Fresco 21 Centrifuge, Thermo Fisher Scientific, Bremen, Germany) for 10 min. at 4ºC. Supernatant was collected and protein concentration determined using a BCA-assay (Thermo Fisher Scientific, Bremen, Germany).

***Protein separation using SDS-PAGE and in-Gel Protein Digestion***

A rapid SDS-PAGE protein pre-fractionation step was performed to reduce the sample complexity prior to the extended gradient LC-MS analysis. Unlike a traditional full protein separation with SDS-PAGE, this rapid pre-fractionation does not achieve a high protein resolution on the gel, yet suffices to reduce the complexity prior to LC-MS analysis. Approximately fifty µg total protein (determined by BCA-assay) obtained from cell lysis were mixed with 10 µL sample loading buffer (12.5% (v/v) glycerol, 1% (w/v) SDS, 0.025% (w/v) bromophenol blue, 2.5% (v/v) β-mercaptoethanol in 30 mM Tris-HCl, pH 6.8) and boiled for 10 min at 90ºC. The denatured and reduced proteins were briefly fractionated using SDS–PAGE employing a combination of a stacking gel (8% polyacrylamid (PA)) and a resolving gel (12% PA). Colloidal Coomassie staining (Neuhoff et al. (1988)) was used to visualize the separated proteins.

After protein staining, 19 bands were excised from the gel (see Figure 1, below in this file). Each excised band was washed with 100 µL NH_4_HCO_3_ (ABC, 100 mM), destained with 150 µL 50:50 (v/v) ACN/ddH_2_O for 15 min and dehydrated for 30 min at room temperature (RT) in a speed-vac (Concentrator Plus, Eppendorf, Hamburg, Germany). Disulfide bonds were reduced by addition of 80 µL reduction buffer (10mM dithiothreitol in 100 mM ABC) with incubation at 56°C for 30 min. Supernatant was discarded and cysteines were alkylated for 20 min in dark at RT with 80 µL alkylation buffer (55 mM iodoacetamide in 100 mM ABC, pH 8). Supernatant was removed and the gel pieces were washed again with 100 µL ABC (100 mM) for 15 min. To shrink and dehydrate the gel pieces, an equal amount of acetonitrile (ACN) was added and incubated for a further of 15 min. Supernatant was removed and the gel pieces were further dried in a speed-vac for 30 min. at RT.

For in-gel digestion, 2 µl of a freshly prepared trypsin solution (25 ng/µl in 50 mM acetic acid) were added and gel pieces were allowed to swell for 15 min. Afterwards, 50 µl digestion buffer (5 % (v/v) ACN in 50 mM ABC, pH 8) was added and proteins were digested overnight at 37°C. Released peptides in supernatant were transferred into new eppendorf tubes, and residual peptides in the gel were extracted twice using 100 µL of: (i) 0.5 % (v/v) trifluoroacetic acid (TFA) in 60:40 (v/v) ACN/ ddH_2_O and (ii) 100 % ACN. The three obtained supernatants of each gel piece were combined and completely dried at 45°C in a speed-vac. The dried extracted peptides of each gel piece were resuspended in 40µl HPLC loading buffer (0.1% (v/v) TFA, 3% (v/v) ACN in ddH_2_O).

***LC-ESI-MS/MS analysis***

In total, three technical replicates were performed. For each replicate, 10 µl were injected for the LC-ESI MS/MS analysis. Peptides were separated by reversed-phase (RP) liquid chromatography on an UltiMate 3000 nano-HPLC system (Dionex, Germering, Germany) and analyzed with an LTQ-Orbitrap Velos equipped with ETD mass spectrometer (Thermo Scientific, Bremen, Germany).

After injection, peptides were trapped on a PepMap C_18_ trap column (300 µm I.D x 5 mm; Acclaim PepMap 100 C_18_, 3 µm, 100 Å, Dionex) and desalted for 10 min by washing with HPLC loading buffer at a flow rate of 30 µl/min. Peptides were then eluted by back flushing and separated on an analytical column (75 µm I.D., 15 cm; Acclaim PepMap RSLC C_18_, 3 µm, 100 Å, Dionex) using a gradient flow rate of 300 nl/min. Two separations were performed using a linear gradient from minute 10 (5% B) to minute 126 (55% B), following column washing (minute 126 to 134 with a linear increase of eluent B to 95%) and LC column re-equilibration for 20 min. at 5% B; a third run was performed using a prolonged linear gradient: 10 to 180 min. (5% B to 55% B), washing (55% B to 95% B within 5 min, isocratic for 15 min.) and re-equilibration (5% B for 14 min.). Eluent A: 0.05% (v/v) formic acid (FA) in ddH_2_O , eluent B: (0.1 % (v/v) FA, 80 % (v/v) ACN in ddH_2_O ).

For MS analysis, two different methods were applied depending on the LC gradient used. For the short gradient, MS^1^ scans were acquired from minute 25 to 125 in the Orbitrap mass analyzer in the m/z range of 300-2000 at a resolution of 60,000 without lock mass correction. A top 5 MS/MS method was written using Xcalibur (Version 2.1) applying Collision Induced Dissociation (CID) and Higher Energy Collision induced Dissociation (HCD) in parallel. For both fragmentation types, wideband activation was enabled. A MS/MS event was triggered if an MS^1^ precursor exceeded a signal intensity of ≥ 500, a charge state ≥ 2. An m/z isolation window of 2 (CID) and 3 (HCD) was used. The default charge state for MS/MS settings was set to 2+. The CID spectra were acquired in the ion trap (LTQ Velos), whereas the HCD spectra were acquired in the Orbitrap with a mass resolution of 15,000 and a first mass value of 100. For CID peptide fragmentation, a normalized collision energy of 36% was applied with an activation Q-value of 0.25 for 10 ms. For HCD a normalized collision energy of 40% was used with an activation time of 0.1 ms. The elevated NCE value for HCD was used to induce a stronger fragmentation for charge states ≥ 3, using the benefit of both CID and HCD fragmentation.

Fragmented precursors were added automatically to an exclusion list for 60 seconds with a repeat count of 2 within 20 seconds (maximum size 500 precursors, isolation width m/z 1.5) to allow more distinct precursors to be selected for fragmentation.

For the longer LC-gradient (3rd technical replicate), the following changes were made: total MS^1^ resolution was lowered to 30,000 for faster data acquisition and CID MS/MS fragmentation NCE was set to 35%. For CID and HCD, the wideband activation was disabled. HCD MS/MS spectra were recorded in Orbitrap mass analyzer using a resolution of 7,500. Lock mass correction of m/z 371.101230 was enabled using cyclomethicone N5 ions generated from ambient air (Mari, Metabolomics 2013).

**Results (Proteome Analysis)**


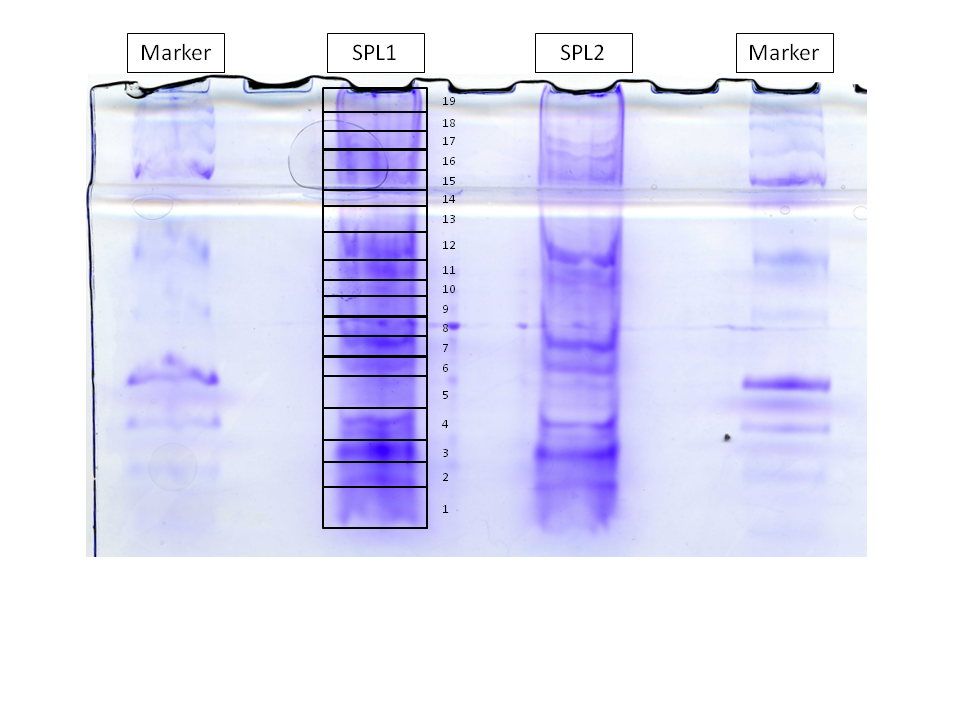


**Figure 1**: 1D-SDS gel separation of approx. 50 µg (determined by BCA-assay) total cell lysate of SH-SY5Y cells. The gel was Coomassie stained. The poor separation and resolution quality of the gel is caused by the short electrophoresis time.

**References (Proteomics)**

Neuhoff, V, Arold, N, Taube, D, and Ehrhardt, W. (1988). *Improved staining of proteins in polyacrylamide gels including isoelectric focusing gels with clear background at nanogram sensitivity using Coomassie Brilliant Blue G-250 and R-250*. Electrophoresis, **9**, 255-262.

Melchior K, Tholey A, Heisel S, Keller A, Lenhof HP, Meese E, Huber CG. (2009). *Proteomic study of human glioblastoma multiforme tissue employing complementary two-dimensional liquid chromatography- and mass spectrometry-based approaches.*  J Proteome Res, **8**, 4604–4614.

Mari A, Lyon D, Fragner L, Montoro P, Piacente S, Wienkoop S, Egelhofer V, Weckwerth W. (2013). *Phytochemical composition of Potentilla anserina L. analyzed by an integrative GC-MS and LC-MS metabolomics platform*. Metabolomics, **9**, 599–607.
