# Supplementary material for: Systems genomics evaluation of the SH-SY5Y neuroblastoma cell line as a model for Parkinson’s disease
Source: BMC Genomics. 2014 Dec 20;15(1):1154. doi: 10.1186/1471-2164-15-1154 (PMC4367834; doi:10.1186/1471-2164-15-1154)
Supplement: Supplementary file 7 — Additional file 7: Network visualization of mutated genes in pathways related to Parkinson’s disease. Each node is a gene and each edge indicates that there is an interaction between them (gene regulatory, protein-protein, metabolic, and signal transduction interactions). Genes annotated with a dark red colour have an exonic mutation, copy number variation or structural variation whereas genes annotated with a light red colour do not have such mutations. (PDF 18 MB) [file 12864_2014_6889_MOESM7_ESM.pdf]

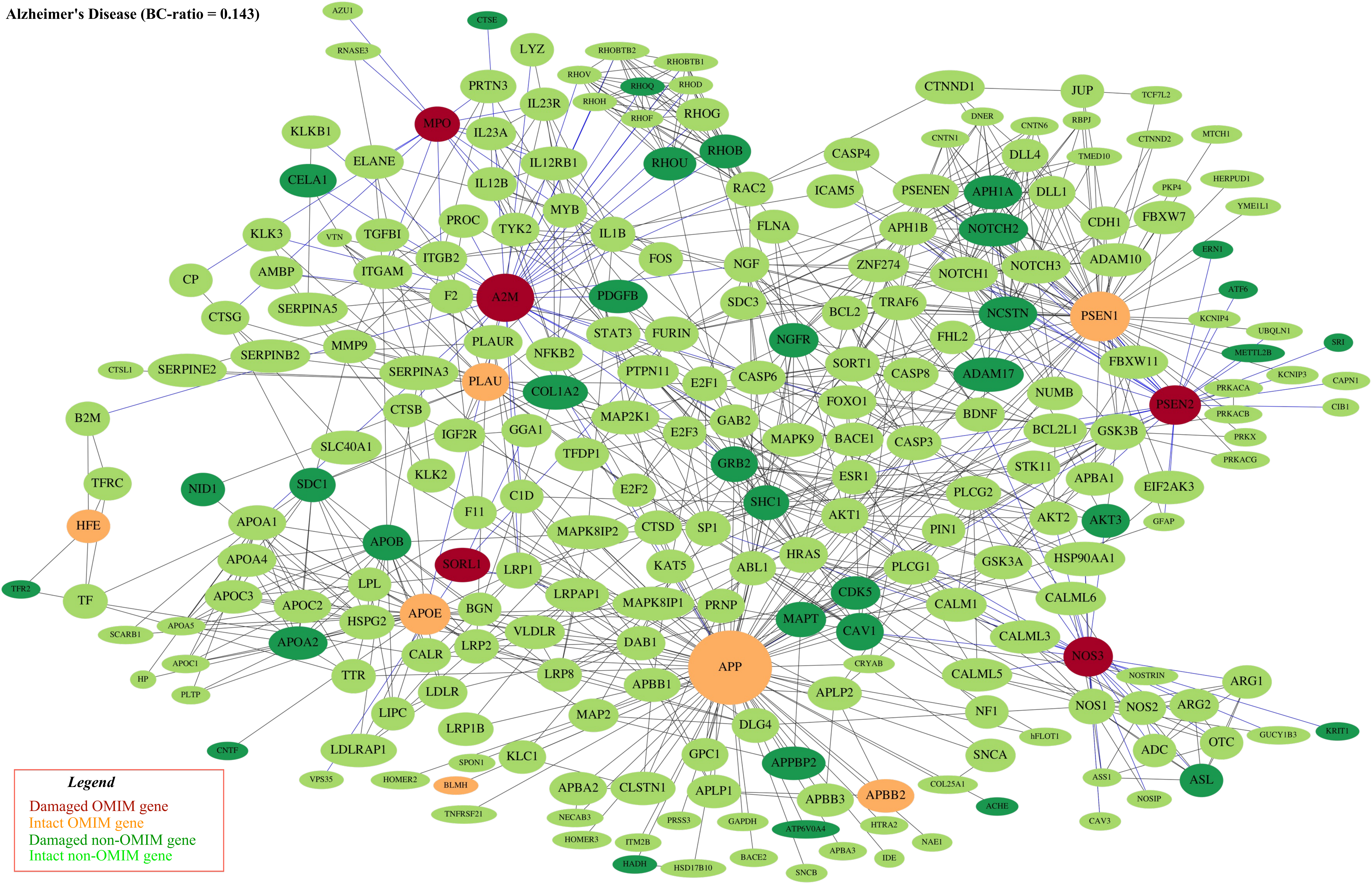

Alzheimer's Disease (BC-ratio = 0.143)
AZU1
CTSE
RNASE3
LYZ
RHOBTB2
RHOBTB1
PRTN3
RHOV
RHOQ
RHOD
CTNND1
JUP
TCF7L2
IL23R
RHOH
RHOG
MPO
RHOF
DNER
CNTN6
RBPJ
KLKB1
IL23A
CNTN1
CTNND2
MTCH1
CASP4
RHOB
DLL4
TMED10
ELANE
RHOU
IL12RB1
CELA1
IL12B
RAC2
ICAM5
PSENEN
APH1A
DLL1
PKP4
HERPUD1
MYB
YME1L1
PROC
FBXW7
CDH1
KLK3
VTN
TGFBI
TYK2
IL1B
FLNA
APH1B
NOTCH2
ERN1
ITGB2
FOS
NGF
ZNF274
NOTCH3
ADAM10
CP
AMBP
ITGAM
NOTCH1
A2M
PDGFB
F2
ATF6
SERPINA5
SDC3
BCL2
TRAF6
NCSTN
PSEN1
CTSG
KCNIP4
STAT3
FURIN
NGFR
FHL2
UBQLN1
MMP9
PLAUR
METTL2B
SRI
SERPINB2
NFKB2
SORT1
FBXW11
CTSL1
SERPINE2
SERPINA3
PTPN11
CASP6
CASP8
ADAM17
KCNIP3
CAPN1
PLAU
E2F1
NUMB
PRKACA
COL1A2
FOXO1
PSEN2
B2M
CTSB
MAP2K1
BDNF
PRKACB
CIB1
GAB2
GGA1
IGF2R
E2F3
BCL2L1
GSK3B
MAPK9
BACE1
CASP3
PRKX
SLC40A1
TFDP1
PRKACG
GRB2
KLK2
ESR1
STK11
TFRC
APBA1
EIF2AK3
NID1
SDC1
E2F2
PLCG2
C1D
SHC1
F11
AKT1
AKT2
AKT3
GFAP
HFE
APOA1
MAPK8IP2
CTSD
SP1
PIN1
APOB
HRAS
SORL1
APOA4
KAT5
LRP1
ABL1
GSK3A
HSP90AA1
PLCG1
TFR2
LPL
CDK5
LRPAP1
TF
APOC3
APOC2
MAPK8IP1
PRNP
CALM1
CALML6
BGN
HSPG2
APOE
MAPT
APOA5
VLDLR
CAV1
SCARB1
APOA2
CALML3
NOS3
LRP2
DAB1
CALR
APP
CRYAB
APOC1
TTR
LRP8
CALML5
NOSTRIN
ARG1
HP
PLTP
APBB1
APLP2
LDLR
NOS1
NOS2
ARG2
NF1
MAP2
LIPC
KRIT1
GUCY1B3
hFLOT1
LRP1B
DLG4
OTC
CNTF
LDLRAP1
ADC
SNCA
KLC1
GPC1
SPON1
APPBP2
ASL
ASS1
VPS35
HOMER2
BLMH
APBA2
CLSTN1
APLP1
COL25A1
APBB2
APBB3
NOSIP
ACHE
TNFRSF21
NECAB3
PRSS3
GAPDH
HTRA2
CAV3
ATP6V0A4
HOMER3
ITM2B
NAE1
APBA3
IDE
BACE2
HADH
HSD17B10
SNCB
Legend
Damaged OMIM gene
Intact OMIM gene
Damaged non-OMIM gene
Intact non-OMIM gene

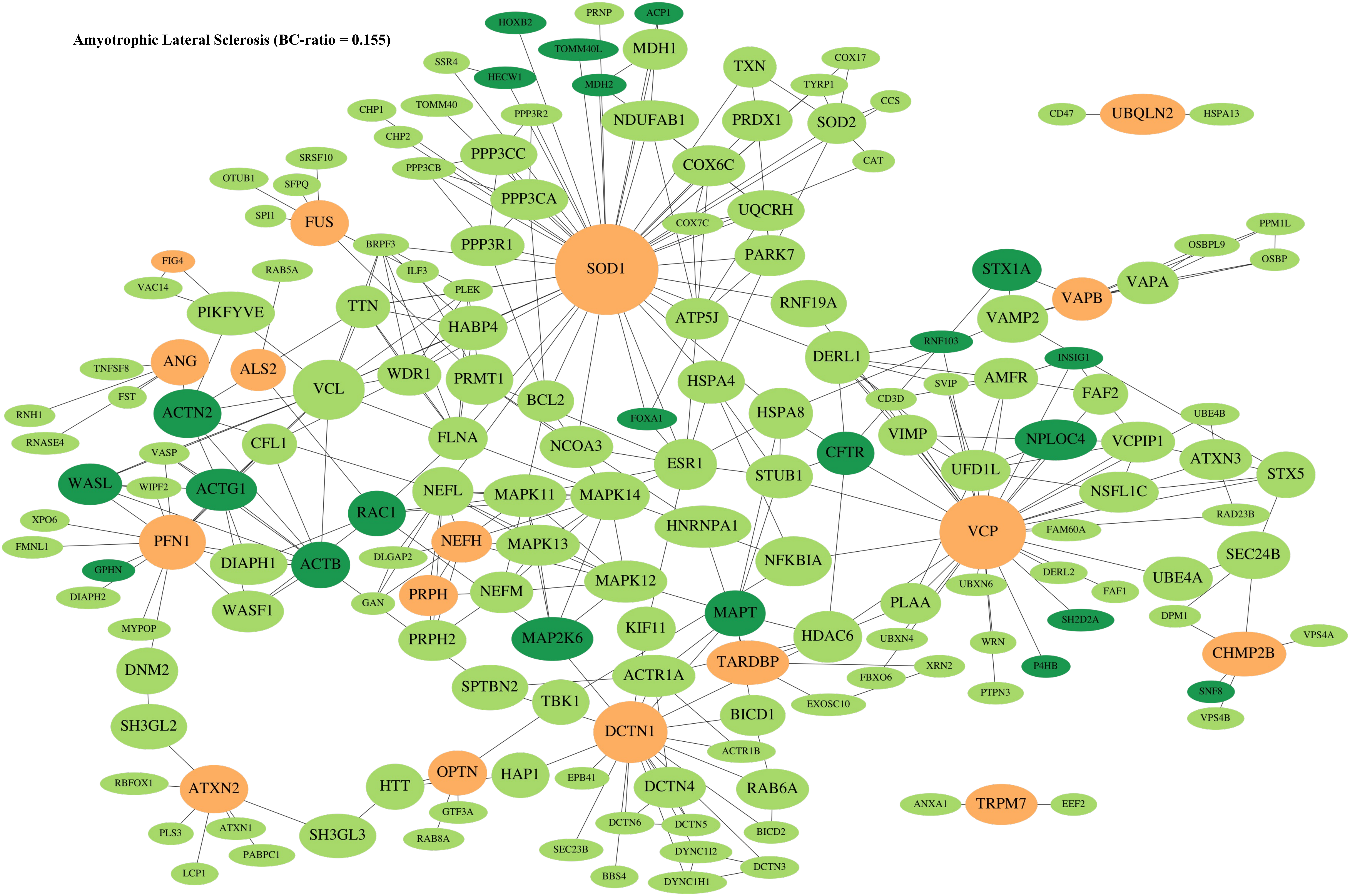
Amyotrophic Lateral Sclerosis (BC-ratio = 0.155)
SOD1
FUS
FIG4
ANG
ALS2
PFN1
NEFH
PRPH
TARDBP
DCTN1
OPTN
ATXN2
VCP
VAPB
CHMP2B
UBQLN2
TRPM7
CD47
HSPA13
ANXA1
EEF2
HOXB2
PRNP
ACP1
TOMM40L
MDH1
MDH2
SSR4
HECW1
TXN
COX17
TYRP1
CCS
SOD2
PRDX1
NDUFAB1
CHP1
TOMM40
PPP3R2
CHP2
PPP3CC
PPP3CB
COX6C
CAT
SRSF10
OTUB1
SFPQ
SPI1
PPP3CA
UQCRH
COX7C
PPP3R1
BRPF3
PARK7
ILF3
PLEK
RAB5A
VAC14
PIKFYVE
TTN
HABP4
ATP5J
RNF19A
STX1A
VAPA
OSBPL9
PPM1L
OSBP
VAMP2
RNF103
DERL1
INSIG1
TNFSF8
RNH1
FST
RNASE4
ACTN2
VCL
WDR1
PRMT1
BCL2
HSPA4
SVIP
AMFR
FAF2
CD3D
FOXA1
HSPA8
VIMP
UBE4B
CFL1
FLNA
NCOA3
ESR1
CFTR
NPLOC4
VCPIP1
ATXN3
STX5
VASP
WASL
WIPF2
ACTG1
NEFL
MAPK11
MAPK14
STUB1
UFD1L
NSFL1C
RAD23B
XPO6
FMNL1
RAC1
HNRNPA1
FAM60A
SEC24B
GPHN
DIAPH1
ACTB
DLGAP2
MAPK13
NFKBIA
DERL2
UBE4A
DIAPH2
GAN
NEFM
MAPK12
MAPT
PLAA
UBXN6
FAF1
DPM1
MYPOP
WASF1
MAP2K6
KIF11
HDAC6
UBXN4
SH2D2A
VPS4A
DNM2
PRPH2
WRN
P4HB
XRN2
SPTBN2
ACTR1A
FBXO6
SNF8
TBK1
EXOSC10
PTPN3
VPS4B
SH3GL2
BICD1
ACTR1B
RBFOX1
HAP1
EPB41
DCTN4
RAB6A
HTT
PLS3
ATXN1
SH3GL3
GTF3A
DCTN6
DCTN5
BICD2
LCP1
PABPC1
RAB8A
SEC23B
DYNC1I2
DCTN3
BBS4
DYNC1H1

Huntington's Disease (BC-ratio = 0.090)

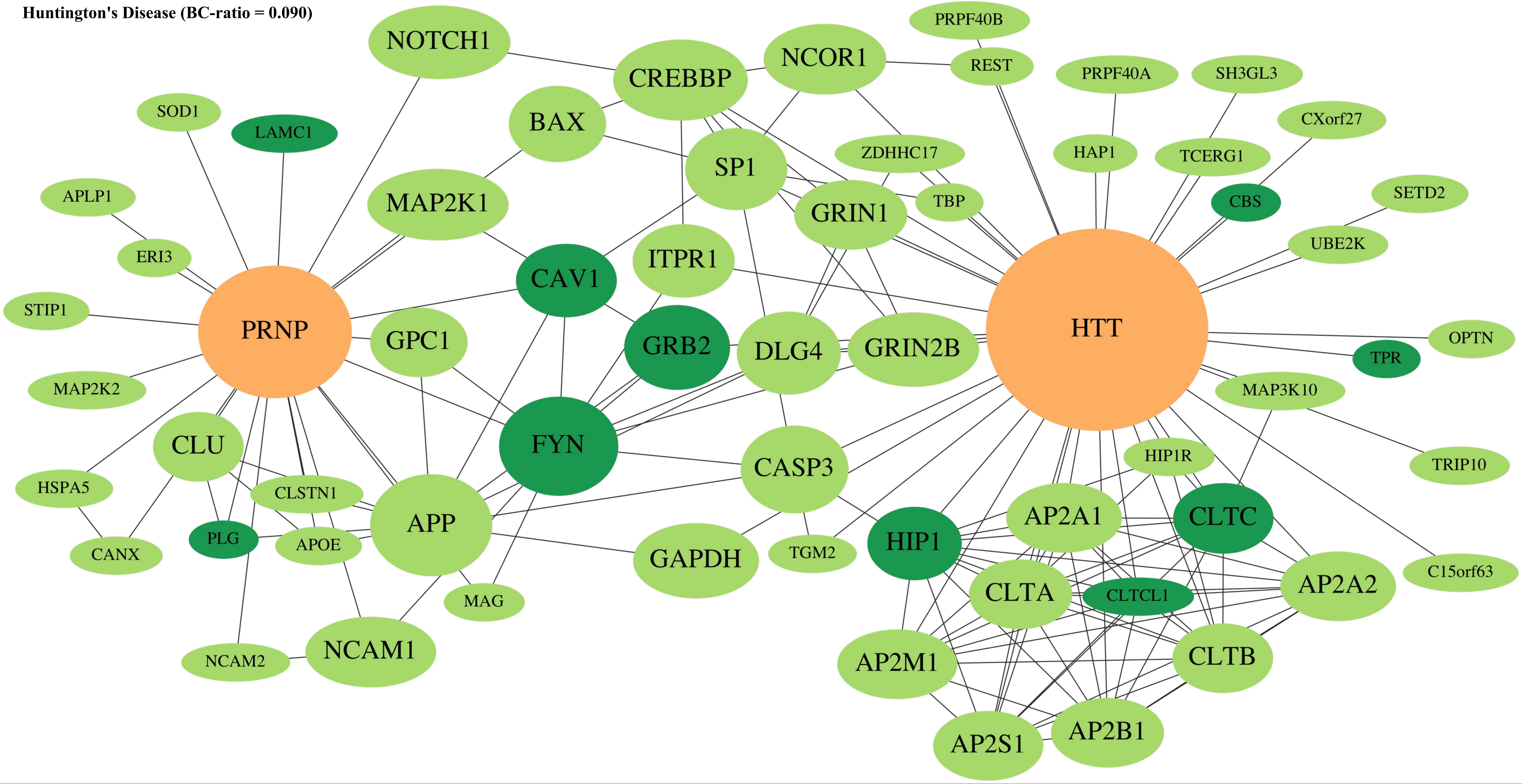

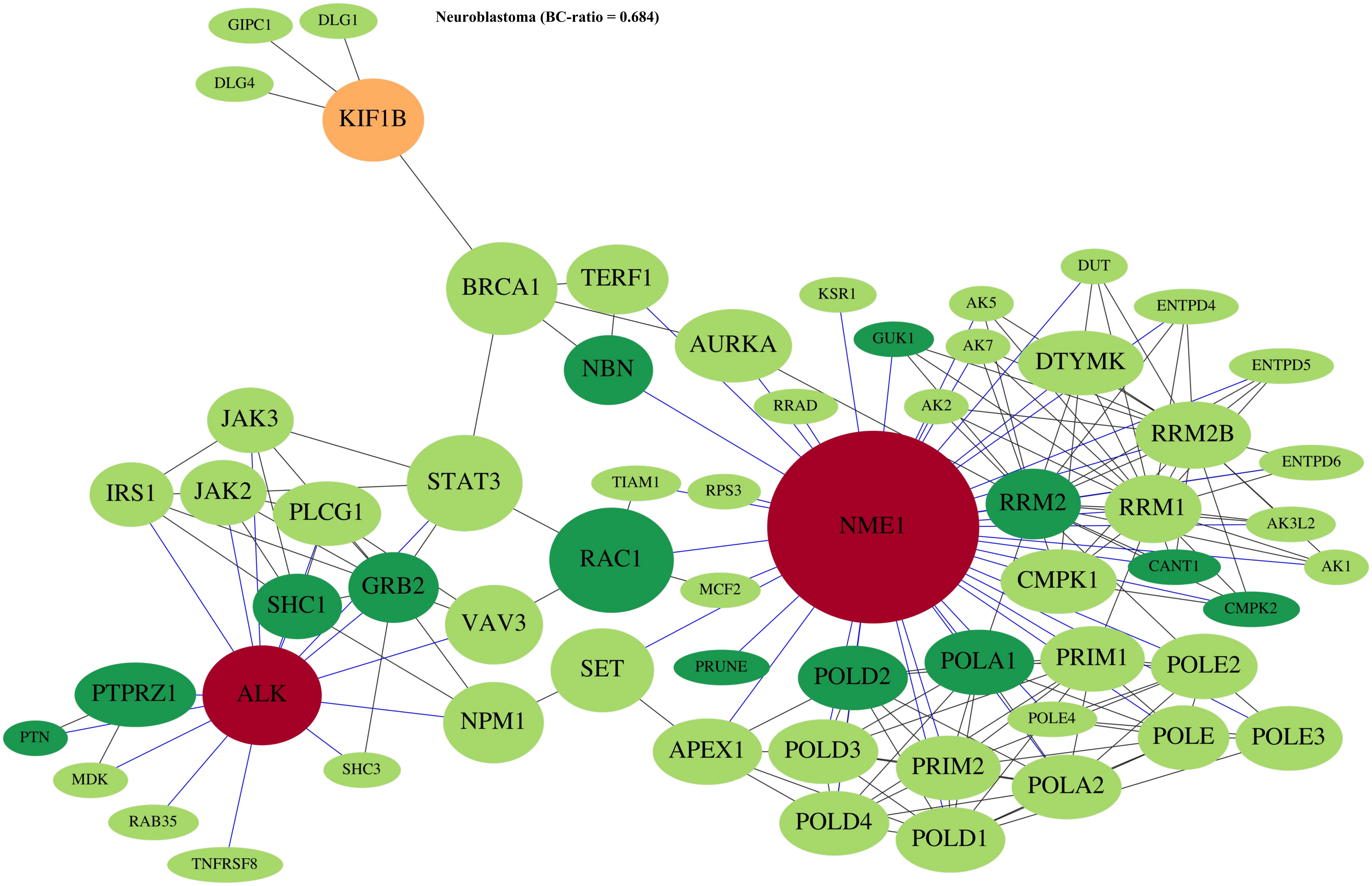

Neuroblastoma (BC-ratio = 0.684)
GIPC1
DLG1
DLG4
KIF1B
BRCA1
TERF1
NBN
AURKA
KSR1
GUK1
AK5
AK7
DUT
DTYMK
ENTPD4
ENTPD5
RRAD
AK2
RRM2B
ENTPD6
JAK3
STAT3
IRS1
JAK2
PLCG1
TIAM1
RPS3
NME1
RRM2
RRM1
AK3L2
RAC1
CANT1
AK1
GRB2
SHC1
VAV3
MCF2
CMPK1
CMPK2
SET
PRUNE
POLD2
POLA1
PRIM1
POLE2
PTPRZ1
ALK
NPM1
POLE4
POLE
POLE3
PTN
APEX1
POLD3
PRIM2
POLA2
MDK
SHC3
RAB35
POLD4
POLD1
TNFRSF8

Parkinson's Disease (BC-ratio = 0.125)

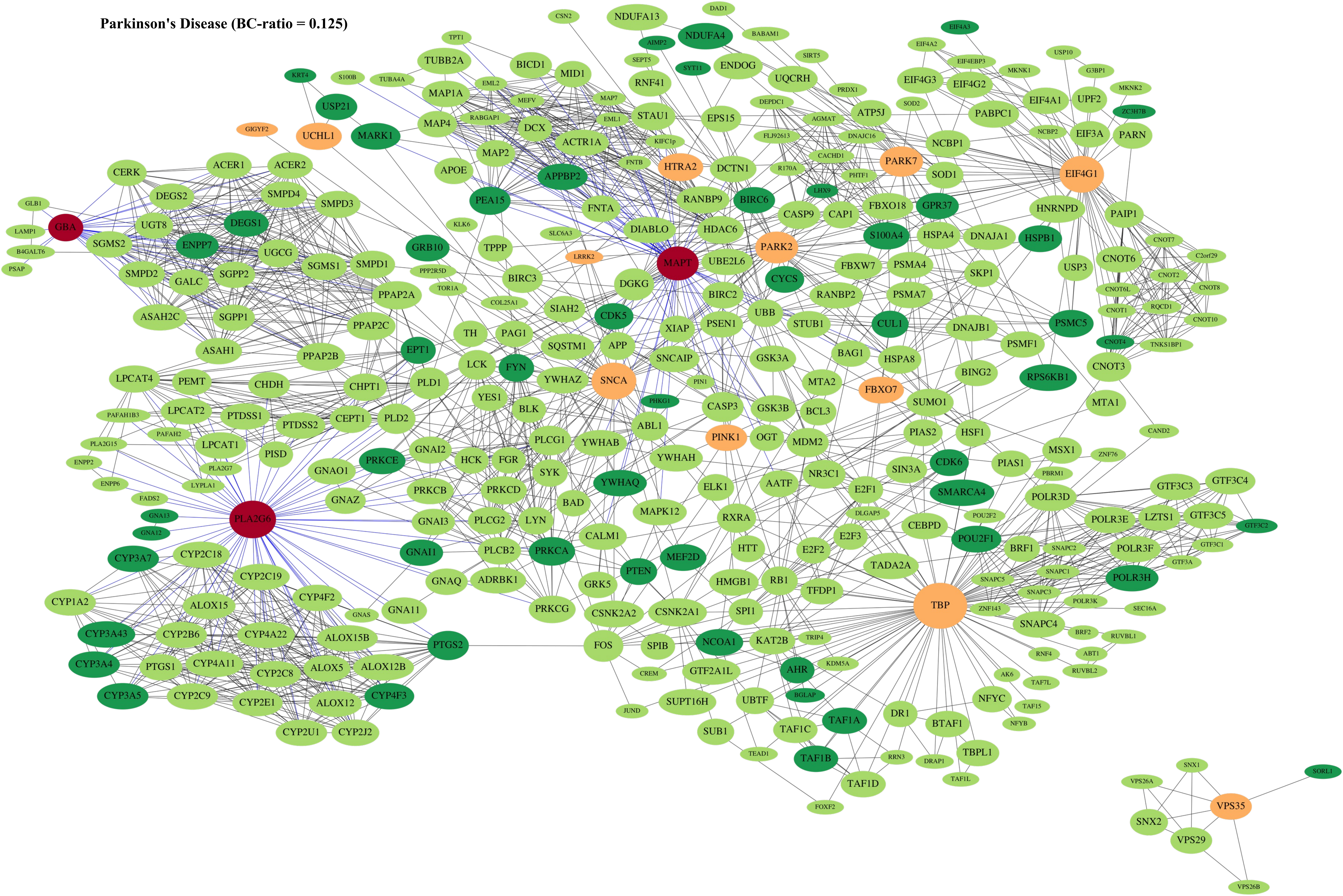

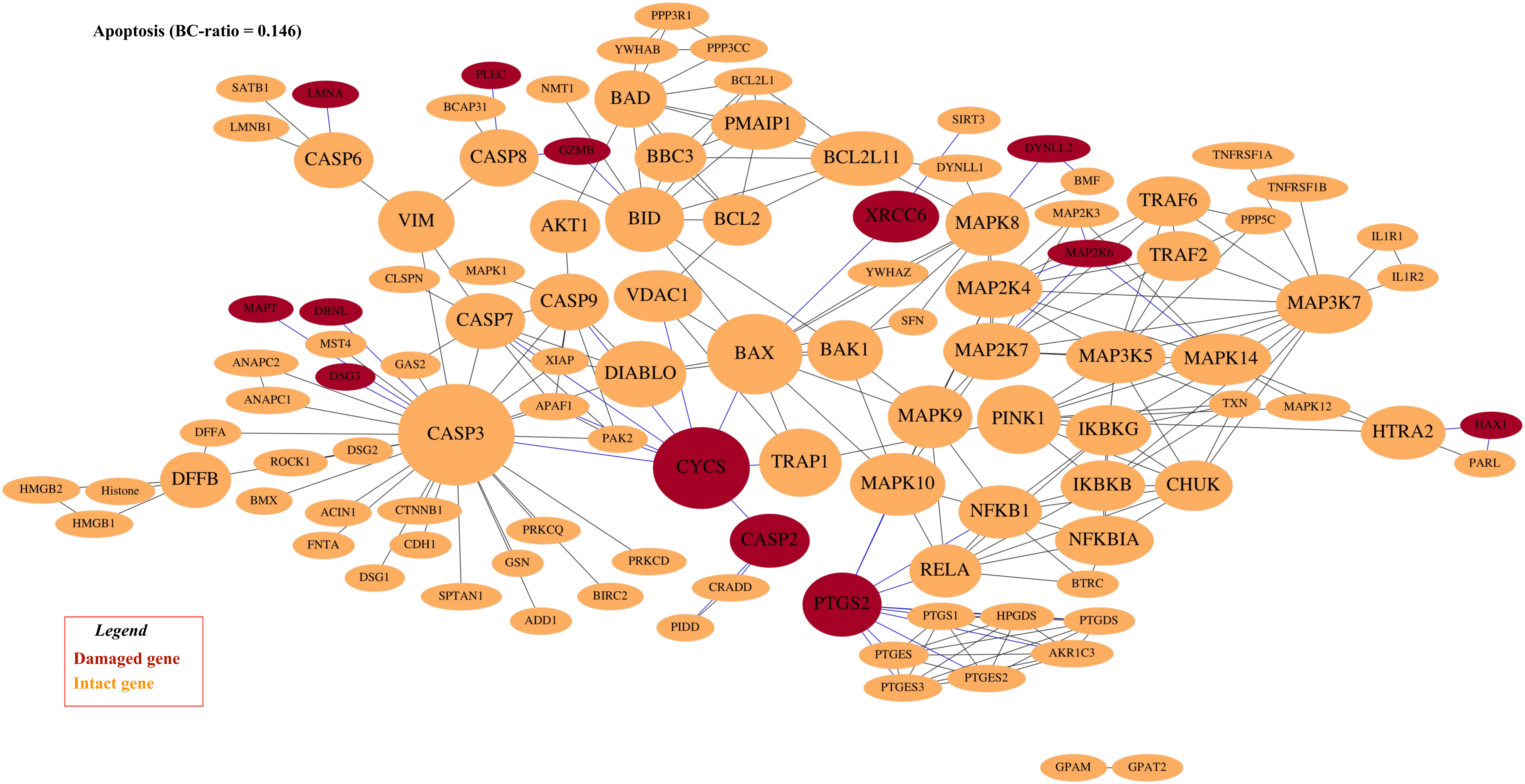
Apoptosis (BC-ratio = 0.146)
PPP3R1
YWHAB
PPP3CC
BCL2L1
BAD
PMAIP1
BBC3
BCL2L11
SIRT3
DYNLL2
DYNLL1
TNFRSF1A
TNFRSF1B
BMF
SATB1
LMNA
LMNB1
CASP6
PLEC
BCAP31
CASP8
NMT1
GZMB
VIM
AKT1
BID
BCL2
XRCC6
MAPK8
MAP2K3
TRAF6
PPP5C
MAP2K6
TRAF2
IL1R1
IL1R2
YWHAZ
MAP2K4
MAP3K7
CLSPN
MAPK1
CASP9
VDAC1
SFN
CASP7
MAPT
DBNL
MST4
GAS2
XIAP
DIABLO
BAX
BAK1
MAP2K7
MAP3K5
MAPK14
ANAPC2
DSG3
ANAPC1
APAF1
MAPK9
PINK1
TXN
MAPK12
IKBKG
HTRA2
HAX1
PARL
DFFA
CASP3
PAK2
CYCS
TRAP1
DSG2
ROCK1
DFFB
HMGB2
Histone
HMGB1
BMX
ACIN1
CTNNB1
MAPK10
IKBKB
CHUK
NFKB1
NFKBIA
PRKCQ
CASP2
FNTA
CDH1
DSG1
GSN
PRKCD
RELA
CRADD
BTRC
SPTAN1
BIRC2
ADD1
PIDD
PTGS2
PTGS1
HPGDS
PTGDS
PTGES
AKR1C3
PTGES2
PTGES3
GPAM
GPAT2
Legend
Damaged gene
Intact gene

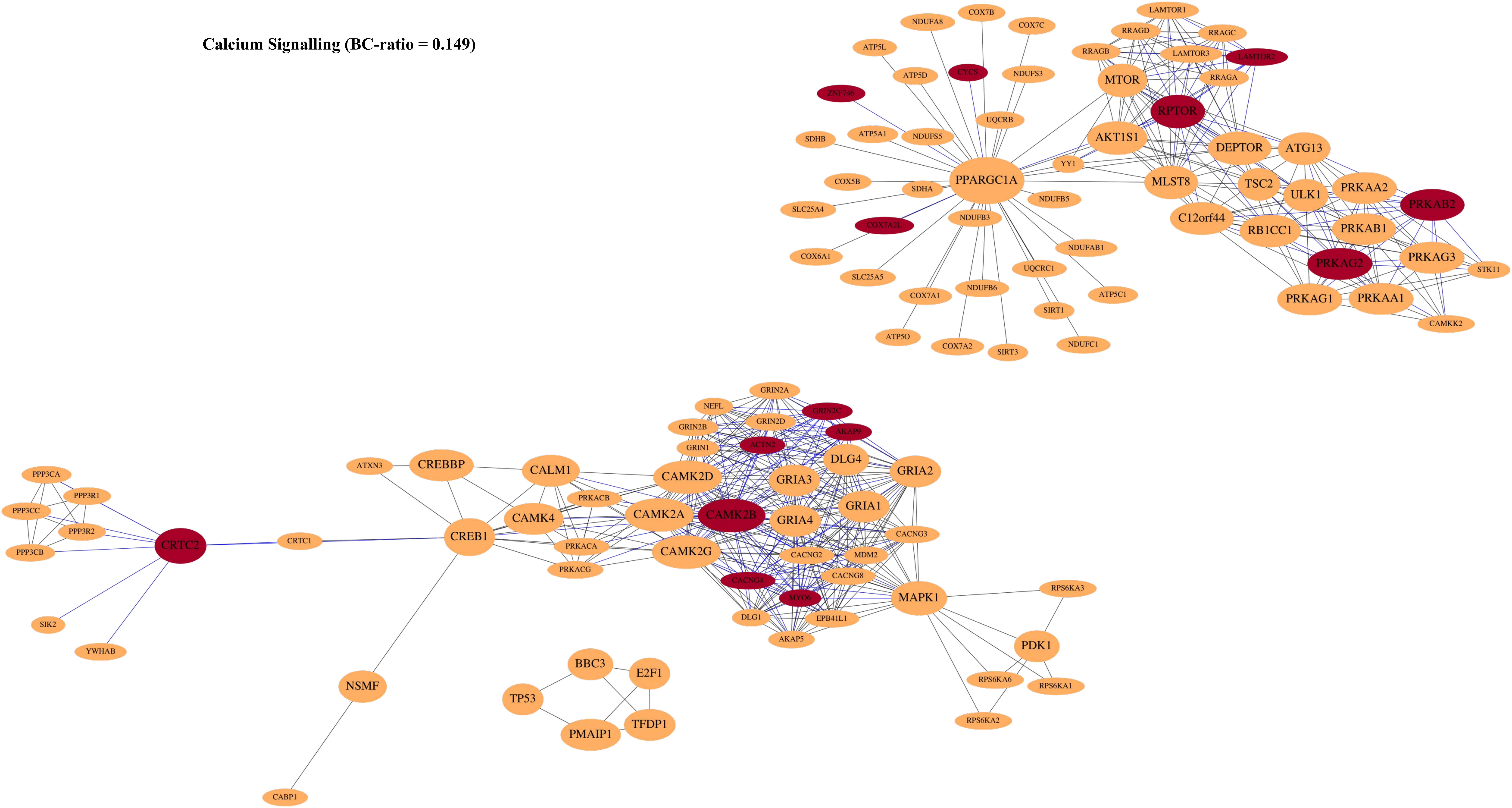
Calcium Signalling (BC-ratio = 0.149)
COX7B
LAMTOR1
NDUFA8
COX7C
RRAGD
RRAGC
ATP5L
RRAGB
LAMTOR3
LAMTOR2
ATP5D
CYCS
NDUFS3
MTOR
RRAGA
ZNF746
RPTOR
UQCRB
ATP5A1
NDUFS5
AKT1S1
SDHB
DEPTOR
ATG13
YY1
COX5B
PPARGC1A
MLST8
SDHA
TSC2
PRKAA2
ULK1
NDUFB5
PRKAB2
SLC25A4
C12orf44
NDUFB3
COX7A2L
RB1CC1
PRKAB1
NDUFAB1
COX6A1
PRKAG3
PRKAG2
UQCRC1
STK11
SLC25A5
ATP5C1
NDUFB6
COX7A1
PRKAG1
PRKAA1
SIRT1
CAMKK2
ATP5O
NDUFC1
COX7A2
SIRT3
GRIN2A
NEFL
GRIN2C
GRIN2D
GRIN2B
AKAP9
ACTN2
GRIN1
DLG4
ATXN3
CREBBP
CALM1
GRIA2
PPP3CA
CAMK2D
GRIA3
PRKACB
PPP3R1
GRIA1
PPP3CC
CAMK4
CAMK2A
CAMK2B
GRIA4
PPP3R2
CREB1
CACNG3
CRTC2
CRTC1
PPP3CB
PRKACA
CAMK2G
CACNG2
MDM2
PRKACG
CACNG8
CACNG4
RPS6KA3
MYO6
MAPK1
SIK2
DLG1
EPB41L1
AKAP5
YWHAB
PDK1
BBC3
E2F1
NSMF
TP53
RPS6KA6
RPS6KA1
TFDP1
PMAIP1
RPS6KA2
CABP1

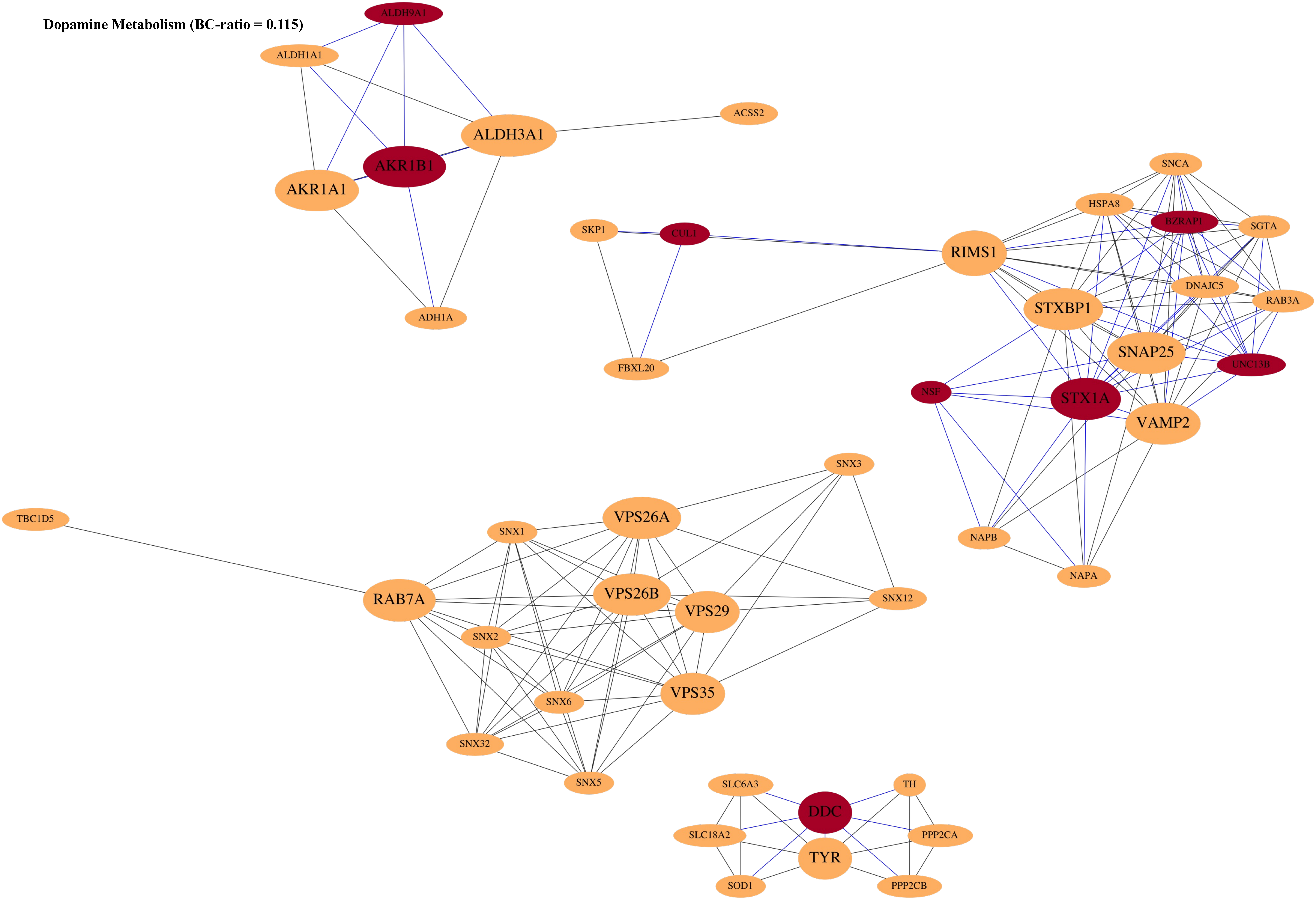
Dopamine Metabolism (BC-ratio = 0.115)
ALDH9A1
ALDH1A1
ACSS2
ALDH3A1
AKR1B1
AKR1A1
ADH1A
SKP1
CUL1
FBXL20
RIMS1
SNCA
HSPA8
BZRAP1
SGTA
DNAJC5
RAB3A
STXBP1
SNAP25
UNC13B
NSF
STX1A
VAMP2
NAPB
NAPA
SNX3
TBC1D5
SNX1
VPS26A
RAB7A
VPS26B
VPS29
SNX12
SNX2
SNX6
VPS35
SNX32
SNX5
SLC6A3
DDC
TH
SLC18A2
PPP2CA
TYR
SOD1
PPP2CB

Glycolysis (BC-ratio = 0.301)

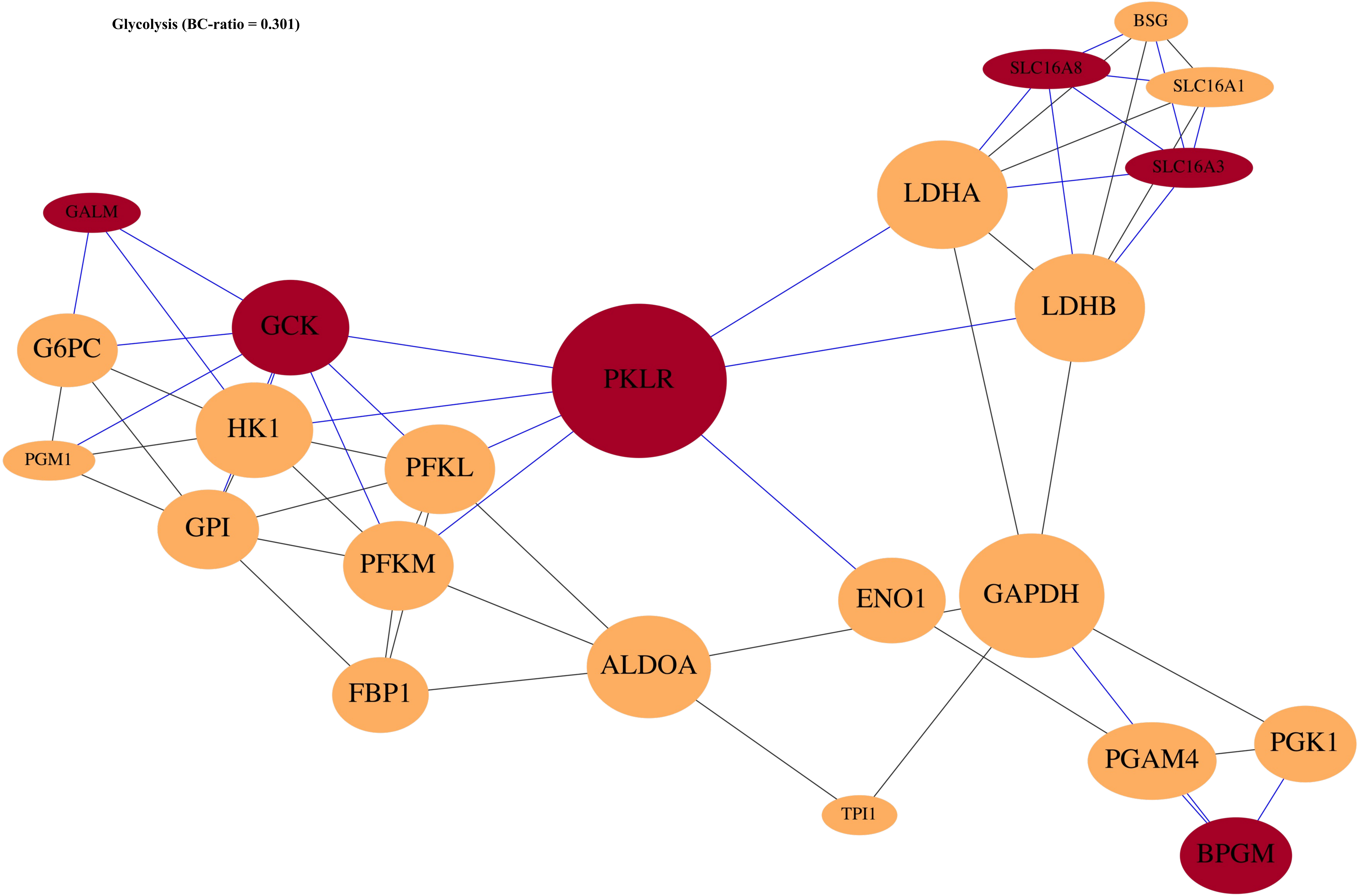

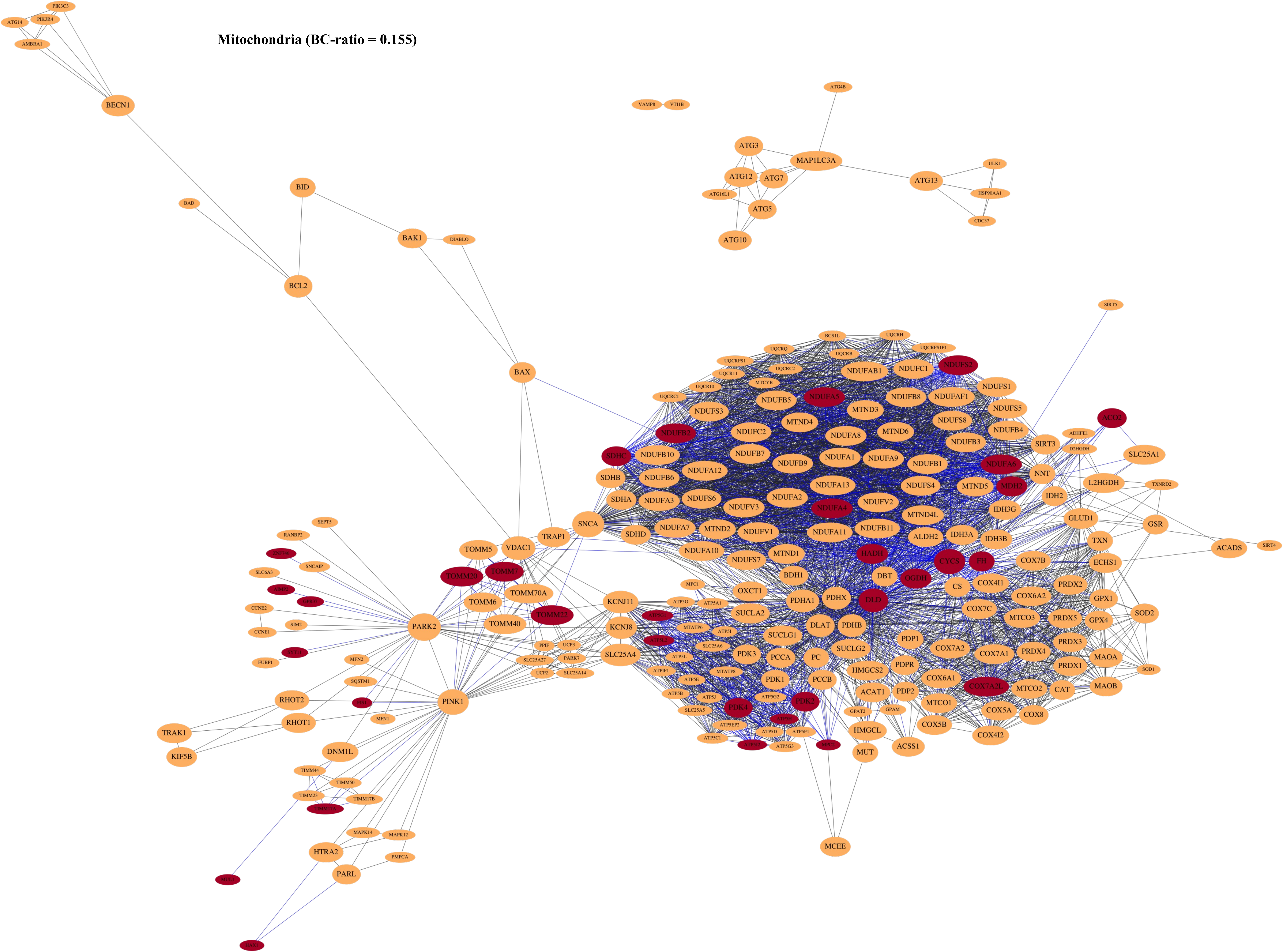
Mitochondria (BC-ratio = 0.155)
PIK3C3
ATG14
PIK3R4
AMBRA1
BECN1
BAD
BID
BCL2
BAK1
DIABLO
BAX
VAMP8
VTI1B
ATG4B
ATG3
MAP1LC3A
ATG12
ATG7
ATG16L1
ATG5
ATG10
ATG13
ULK1
HSP90AA1
CDC37
SIRT5
BCS1L
UQCRH
UQCRQ
UQCRB
UQCRFS1P1
UQCRFS1
UQCRC2
UQCR11
NDUFAB1
NDUFC1
NDUFS2
MTCYB
UQCR10
UQCRC1
NDUFB5
NDUFA5
NDUFB8
NDUFAF1
NDUFS1
NDUFS3
MTND3
NDUFS8
NDUFS5
MTND4
NDUFB2
NDUFC2
NDUFA8
MTND6
NDUFB4
ACO2
NDUFB3
ADHFE1
SIRT3
D2HGDH
SDHC
NDUFB10
NDUFB7
NDUFB9
NDUFA1
NDUFA9
NDUFB1
NDUFA6
SLC25A1
NNT
SDHB
NDUFB6
NDUFA12
NDUFA13
NDUFS4
MTND5
MDH2
L2HGDH
TXNRD2
SDHA
NDUFA3
NDUFS6
NDUFA2
NDUFV3
NDUFA4
NDUFV2
IDH2
IDH3G
MTND4L
GLUD1
SNCA
GSR
SEPT5
NDUFA7
MTND2
NDUFV1
NDUFA11
NDUFB11
ALDH2
IDH3A
IDH3B
RANBP2
TRAP1
SDHD
TXN
ACADS
SIRT4
ZNF746
VDAC1
TOMM5
NDUFA10
MTND1
HADH
CYCS
FH
COX7B
ECHS1
SNCAIP
NDUFS7
SLC6A3
TOMM7
TOMM20
BDH1
DBT
OGDH
AIMP2
MPC1
OXCT1
CS
COX4I1
PRDX2
TOMM70A
GPR37
TOMM6
KCNJ11
PDHA1
PDHX
DLD
COX6A2
GPX1
CCNE2
ATP5O
ATP5A1
SOD2
TOMM22
SUCLA2
COX7C
MTCO3
PRDX5
SIM2
PARK2
TOMM40
ATP5G1
MTATP6
KCNJ8
DLAT
PDHB
GPX4
CCNE1
ATP5I
SUCLG1
PDP1
PRDX3
ATP5L2
PPIF
UCP3
SLC25A6
SYT11
SLC25A27
PARK7
SLC25A4
ATP5L
PDK3
PCCA
PC
SUCLG2
COX7A2
COX7A1
PRDX4
MAOA
FUBP1
MFN2
ATPIF1
MTATP8
HMGCS2
PDPR
PRDX1
SOD1
UCP2
SLC25A14
SQSTM1
ATP5E
PDK1
PCCB
COX6A1
COX7A2L
MTCO2
CAT
MAOB
RHOT2
ATP5B
ATP5J
ATP5G2
ACAT1
PDP2
FIS1
PINK1
PDK4
PDK2
MTCO1
MFN1
SLC25A5
ATP5H
GPAT2
GPAM
COX5A
COX8
RHOT1
ATP5EP2
COX5B
TRAK1
ATP5D
ATP5F1
HMGCL
COX4I2
ATP5C1
ATP5G3
MPC2
DNM1L
ATP5J2
ACSS1
MUT
KIF5B
TIMM44
TIMM50
TIMM23
TIMM17B
TIMM17A
MAPK14
MAPK12
MCEE
HTRA2
PMPCA
PARL
MFN3
HAX1

ROS metabolism (BC-ratio = 0.000)

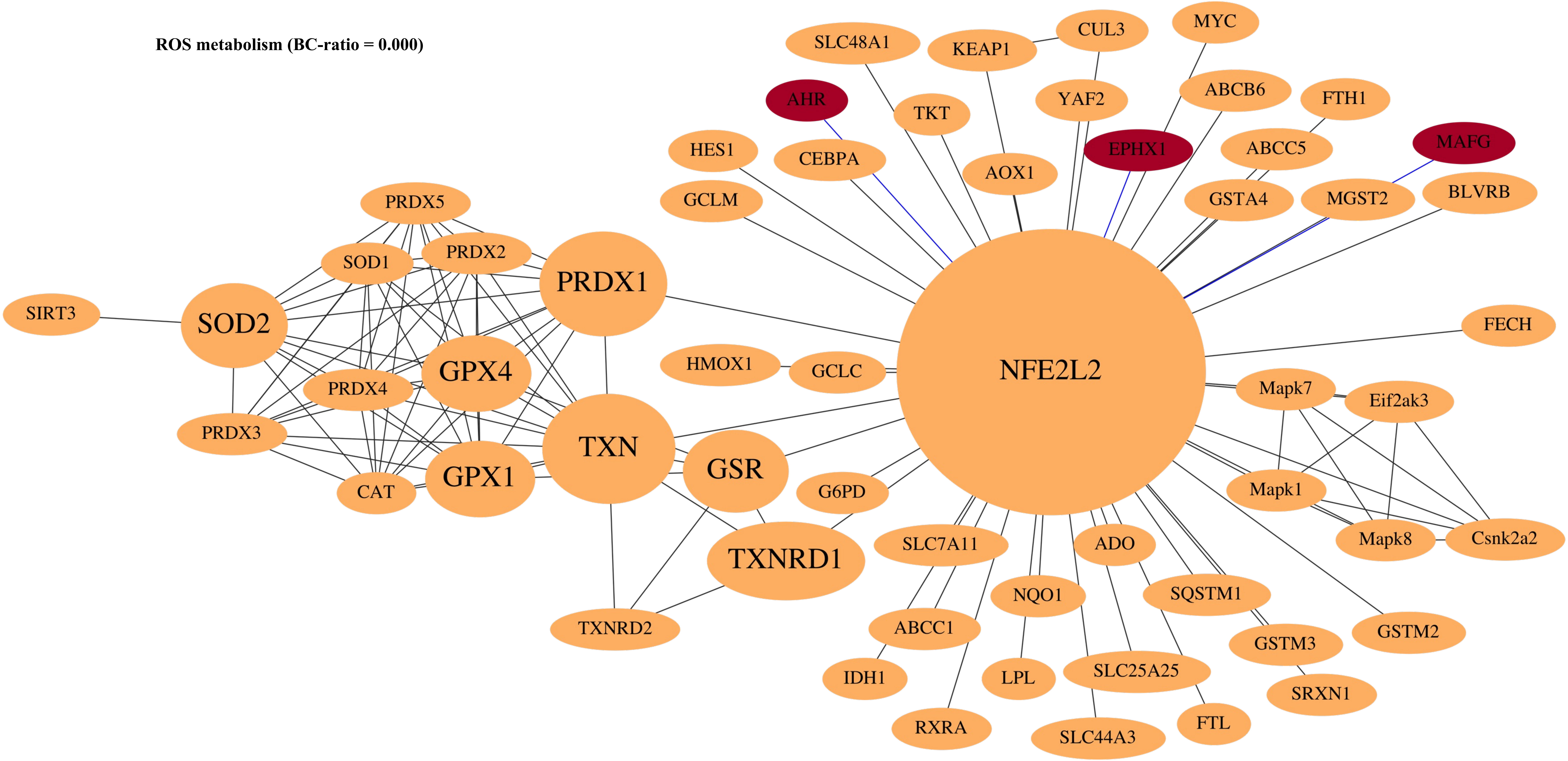

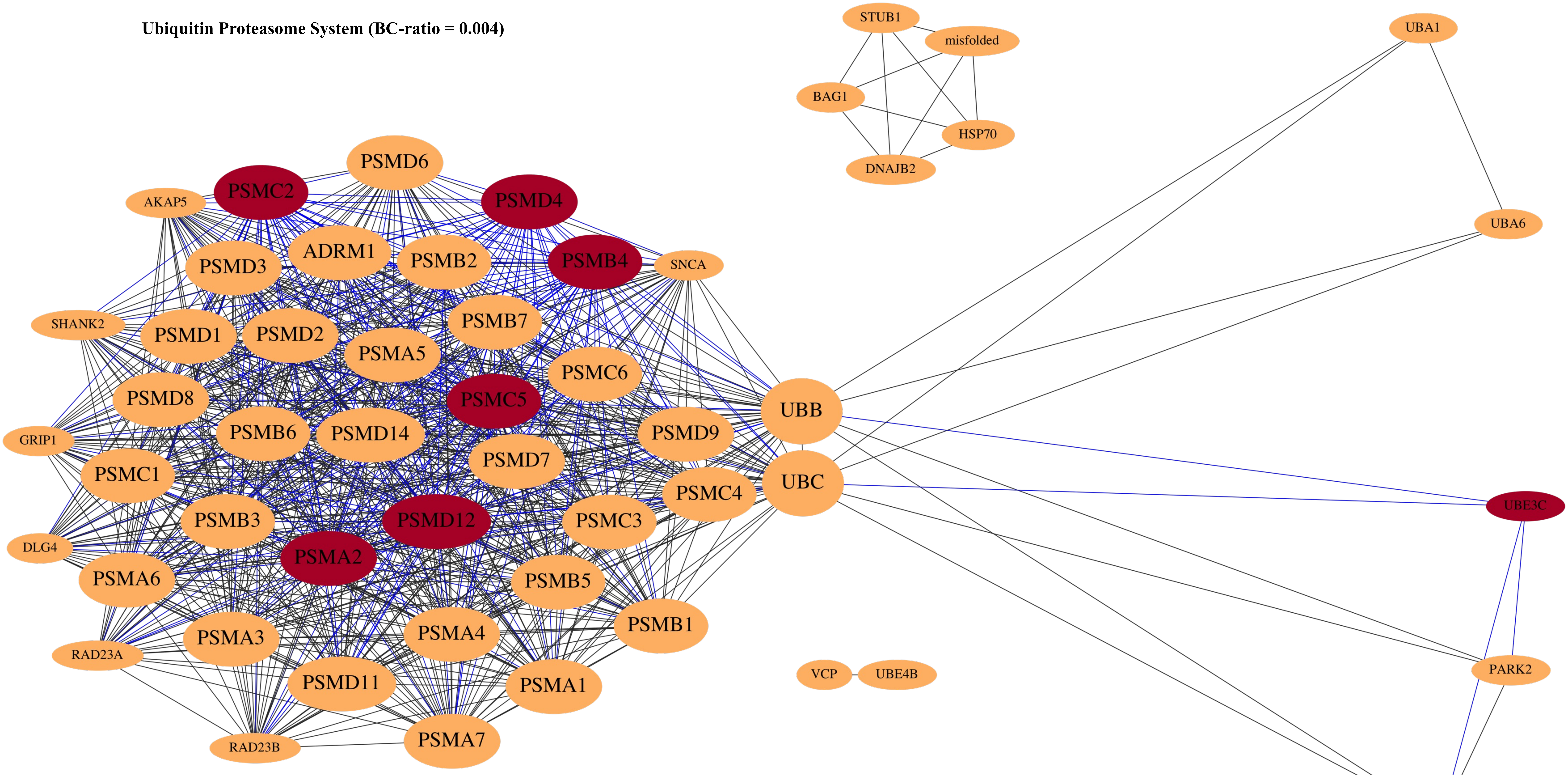
Ubiquitin Proteasome System (BC-ratio = 0.004)
STUB1
misfolded
BAG1
HSP70
DNAJB2
UBA1
UBA6
PSMD6
PSMC2
PSMD4
AKAP5
ADRM1
PSMD3
PSMB2
PSMB4
SNCA
SHANK2
PSMD1
PSMD2
PSMB7
PSMA5
PSMC6
PSMD8
PSMC5
UBB
GRIP1
PSMB6
PSMD14
PSMD9
PSMD7
PSMC1
UBC
PSMC4
UBE3C
PSMB3
PSMD12
PSMC3
DLG4
PSMA2
PSMA6
PSMB5
PSMB1
PSMA3
PSMA4
RAD23A
VCP
UBE4B
PARK2
PSMD11
PSMA1
PSMA7
RAD23B
UBE3A
